# Molecular profiling of stem cell-derived retinal pigment epithelial cell differentiation established for clinical translation

**DOI:** 10.1016/j.stemcr.2022.05.005

**Published:** 2022-06-14

**Authors:** Sandra Petrus-Reurer, Alex R. Lederer, Laura Baqué-Vidal, Iyadh Douagi, Belinda Pannagel, Irina Khven, Monica Aronsson, Hammurabi Bartuma, Magdalena Wagner, Andreas Wrona, Paschalis Efstathopoulos, Elham Jaberi, Hanni Willenbrock, Yutaka Shimizu, J. Carlos Villaescusa, Helder André, Erik Sundstrӧm, Aparna Bhaduri, Arnold Kriegstein, Anders Kvanta, Gioele La Manno, Fredrik Lanner

**Affiliations:** 1 Department of Clinical Sciences, Intervention and Technology, Karolinska Institutet, 17177 Stockholm, Sweden; 2 Gynecology and Reproductive Medicine, Karolinska Universitetssjukhuset, 14186 Stockholm, Sweden; 3 Department of Clinical Neuroscience, Division of Eye and Vision, St. Erik Eye Hospital, Karolinska Institutet, 11282 Stockholm, Sweden; 4 Laboratory of Neurodevelopmental Systems Biology, Brain Mind Institute, School of Life Sciences, École Polytechnique Fédérale de Lausanne (EPFL), 1015 Lausanne, Switzerland; 5 Center for Hematology and Regenerative Medicine, Department of Medicine, Karolinska Institutet, 17177 Stockholm, Sweden; 6 Cell Therapy R&D, Novo Nordisk A/S, Måløv 2760, Denmark; 7 Department of Neurobiology, Care Sciences and Society, Karolinska Institutet, 17177 Stockholm, Sweden; 8 Department of Neurology, University of California, San Francisco, CA, USA; 9 Eli and Edythe Broad Center for Regeneration Medicine and Stem Cell Research, University of California, San Francisco, CA, USA; 10 Ming Wai Lau Center for Reparative Medicine, Stockholm node, Karolinska Institutet, 17177 Stockholm, Sweden

**Keywords:** human embryonic stem cell-derived retinal pigment epithelial cell, Large-eyed model, single-cell RNA sequencing, differentiation protocol dynamics, cellular profiling and transcriptome, retinal progenitor cells, cellular therapy, clinical translation, age-related macular degeneration, subretinal injection

## Abstract

Human embryonic stem cell-derived retinal pigment epithelial cells (hESC-RPE) are a promising cell source to treat age-related macular degeneration (AMD). Despite several ongoing clinical studies, a detailed mapping of transient cellular states during *in vitro* differentiation has not been performed. Here, we conduct single-cell transcriptomic profiling of an hESC-RPE differentiation protocol that has been developed for clinical use. Differentiation progressed through a culture diversification recapitulating early embryonic development, whereby cells rapidly acquired a rostral embryo patterning signature before converging toward the RPE lineage. At intermediate steps, we identified and examined the potency of an NCAM1^+^ retinal progenitor population and showed the ability of the protocol to suppress non-RPE fates. We demonstrated that the method produces a pure RPE pool capable of maturing further after subretinal transplantation in a large-eyed animal model. Our evaluation of hESC-RPE differentiation supports the development of safe and efficient pluripotent stem cell-based therapies for AMD.

## Highlights

• Transcriptional analysis of hESC-RPE differentiation benchmarked to *in vivo* cells
• NCAM1 emerges as a cell-surface marker of multipotent neuroepithelial progenitors
• hESC-RPE cells are obtained through a divergence-convergence process 
• hESC-RPE further mature *in vivo* upon subretinal injection into the rabbit eye

## Teaser

Lanner, La Manno, and colleagues show that stem cell-derived retinal pigment epithelial cells (hESC-RPE) initially recapitulate human retinal embryonic stages, converge toward >98% RPE *in vitro*, and further mature *in vivo*. Transcriptomic analysis of multiple lines and protocols offer insights into different aspects of the heterogeneity dynamics during the differentiation process.

## Introduction

The eye, by virtue of its accessibility and isolated anatomical location, has emerged as a promising organ for gene- and cell-based therapies. A pathology that is particularly promising to tackle with these approaches is age-related macular degeneration (AMD), which causes severe vision loss and affects more than 180 million people globally (Gehrs et al., 2006). The dry form of the disease, for which no treatment is available, affects 80%–90% of advanced patients and is characterized by well-demarcated areas of retinal pigment epithelium (RPE) loss and retinal degeneration (Ambati et al., 2003; Sunness, 1999). Human pluripotent stem cell (hPSC)-derived RPE cells are thus of high interest for cell replacement treatment options, and currently are being tested in several clinical trials (Maeda et al., 2022).

Efforts have been made toward developing strategies to ensure high-purity RPE products, but focus on final product composition has overshadowed the characterization of intermediate stages appearing before a final steady state is reached (Choudhary and Whiting, 2016; Plaza Reyes et al., 2020a). Single-cell RNA sequencing (scRNA-seq) can systematically phenotype cell populations, and its genome-wide readout is crucial to explore *in vitro* differentiation (Kulkarni et al., 2019; Kumar et al., 2017; Lederer and La Manno, 2020). For example, scRNA-seq can determine whether cells follow developmental or non-canonical paths to maturation (Cuomo et al., 2020). Analyzing cell pools at intermediate stages might expose interesting relations between *in vitro* and *in vivo* processes and help to correctly identify potential risks for clinical translation (Begbie, 2013; La Manno et al., 2016). Comprehensive single-cell atlases of embryonic and postnatal neurodevelopment are also fundamental to assisting in the evaluation of gene expression profiles measured *in vitro* (La Manno et al., 2021; Zeisel et al., 2018). Recent work has sought to decompose cellular heterogeneity of the embryonic and postnatal eye with scRNA-seq, but the similarity between transient states arising in development and human pluripotent stem cell (hPSC)-derived intermediates en route to RPE lineage has not yet been evaluated (Hu et al., 2019; Lidgerwood et al., 2021; Lukowski et al., 2019; Voigt et al., 2019).

In this study, we performed scRNA-seq analyses during human embryonic stem cell RPE (hESC-RPE) differentiation using a protocol established for clinical translation (Plaza Reyes et al., 2020a, 2020b). We demonstrate that cells follow embryonic retinal specification, reaching a mature, pigmented RPE phenotype and even undergoing further maturation toward an adult-like state upon subretinal transplantation into the albino rabbit eye. These findings provide valuable insight into the developmental program of hESC-RPE differentiation and illustrate the required high quality of the derived cells to be used as a future certified clinical product.

## Results

### Human embryonic stem cells traverse gene expression space and sequentially mature into retinal pigment epithelium

**Figure 1:**
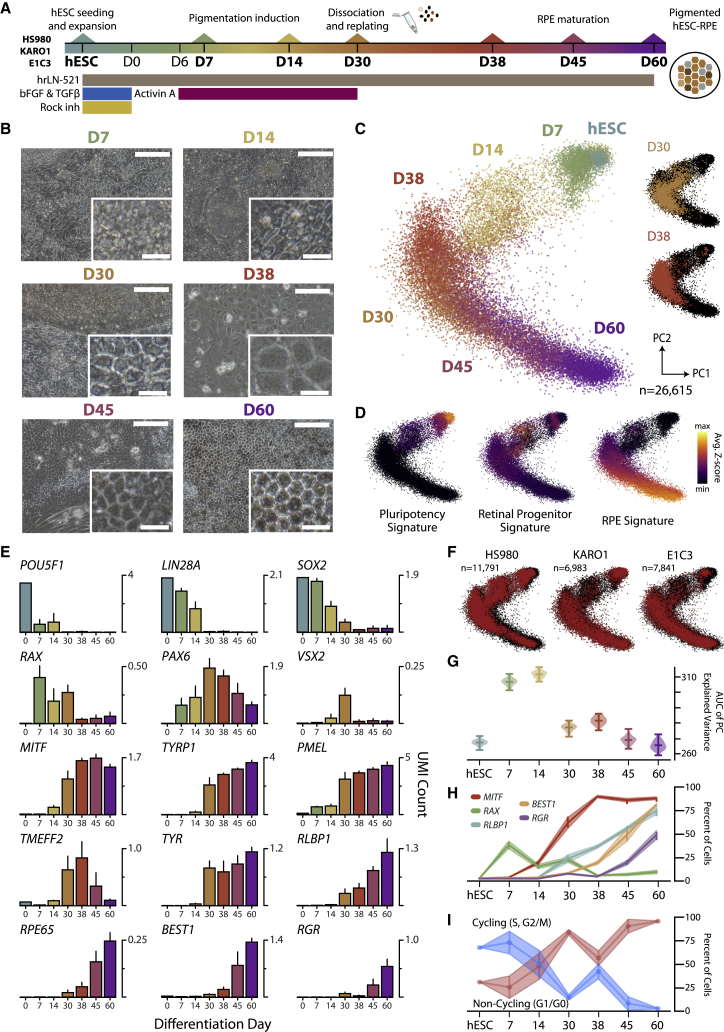
Global scRNA-seq characterization of hESC-RPE differentiation trajectory (A) Schematic of the hESC-RPE differentiation experimental protocol where scRNA-seq was performed at the seven time points (bolded; D, day) in three cell lines: HS980, KARO1, and E1C3. (B) Brightfield images during HS980 differentiation. Scale bars, 100 μm; inset scale bars, 20 μm. (C) Principal component (PC) representation of 26,615 single cells across three lines using 2,000 cv-mean enriched genes. (D) PC showing signature scores for pluripotency, retinal progenitors, and RPE cells. (E) Bar graphs showing average normalized gene expression of pluripotent, retinal progenitor, and RPE markers in scRNA-seq data. Error bars represent standard deviation of the mean across three lines, except for the hESC time point. (F) PC plot colored by cell line in red. (G) Plot showing cumulative explained variance curve for each time point and all lines, applied to estimate how much variance accumulates over sets of correlated genes (biological-driven variability), as opposed to uniformly across genes (white noise). (H) Line plots showing percentage of cells positive for retinal marker genes at each time point. (I) Line plots showing scRNA-seq-based cell-cycle phase assignment. Cycling: S and G_2_/M; non-cycling: G_1_/G_0_. Intervals in (H) and (I) represent the 95% confidence intervals. See also Figure S1.

To examine the process by which hESC-RPE are generated (Plaza Reyes et al., 2020a, 2020b), we performed scRNA-seq throughout differentiation (Figure 1A). We profiled differentiation of one research and two clinical grade cell lines (HS980, KARO1, and E1C3, respectively) at six time points (day 7 [D7], D14, D30, D38, D45, and D60; Table S1). Morphological evaluation using cobblestone junction scores confirmed that changes in cell shape and size followed differentiation as cells progressively assumed a tighter cobblestone monolayer of pigmented cells (Joshi et al., 2016) (Figures 1B, S1A, and S1B).

Assessment of 26,615 single-cell transcriptomes showed that cells traversed a reduced gene expression space from the pluripotent state toward a mature RPE identity (Figures 1C, S1C, and S1D). Gene signature scores detected an initial loss of the pluripotency signature, increased progenitor status at intermediate days, and a later rise of mature RPE (Figures 1D and S1E). Temporal assessment of gene expression confirmed distinct expression waves, with pluripotency genes (*POU5F1*, *LIN28A*, *SOX2*) leading and being downregulated in favor of progenitor genes (*RAX*, *PAX6*, *VSX2*), eventually trailed by early (*MITF*, *TYRP1*, *PMEL*, *TMEFF2*), intermediate (*TYR*, *RLBP1*), and late (*RPE65*, *BEST1*, *RGR*) RPE maturation genes (Figure 1E). Cells from all three lines were uniformly distributed along the global representation, demonstrating robustness and reproducibility of the protocol through a path consistent with the intended differentiation (Figure 1F).

### Heterogeneity analysis reveals changes in cell diversity during differentiation

Interestingly, we observed deviations from a uniform progression toward RPE. D30 cells appeared more morphologically differentiated toward RPE than D38 cells, likely a response to dissociating and replating (Figures 1C and S1A–S1E). A subset of intermediate cells did not exhibit a strong signature for any of the three global identities considered, suggesting a complex differentiation process and presence of additional cell types (Figure 1D).

To quantify the biological heterogeneity observed, we calculated the variance accumulated in correlated gene modules (see supplemental experimental procedures and Figure S1F). This revealed that initial (hESCs) and endpoint (D60) cells harbored a lower heterogeneity compared to intermediate days (Figure 1G). While a large decrease in heterogeneity was detected from D14 to D30, suggesting an initial convergence toward RPE fate, we observed a slight increase from D30 to D38, thus hinting at an effect of cell dissociation, replating, or Activin A removal on cell composition. A similar pattern was observed with cobblestone junction scores (Figure S1A). Initial and endpoint samples also had mutually exclusive and uniform expression of pluripotency and RPE genes (Figures 1H and S1G–S1I). This was consistent with proliferation trends: a decreased fraction of cycling cells from hESC to D30, followed by an increase from D30 to D38, and finally a second decline from D38 to D60 (Figures 1I and S1J).

### Early differentiation recapitulates cellular diversity of the rostral neural tube and optic vesicle

**Figure 2:**
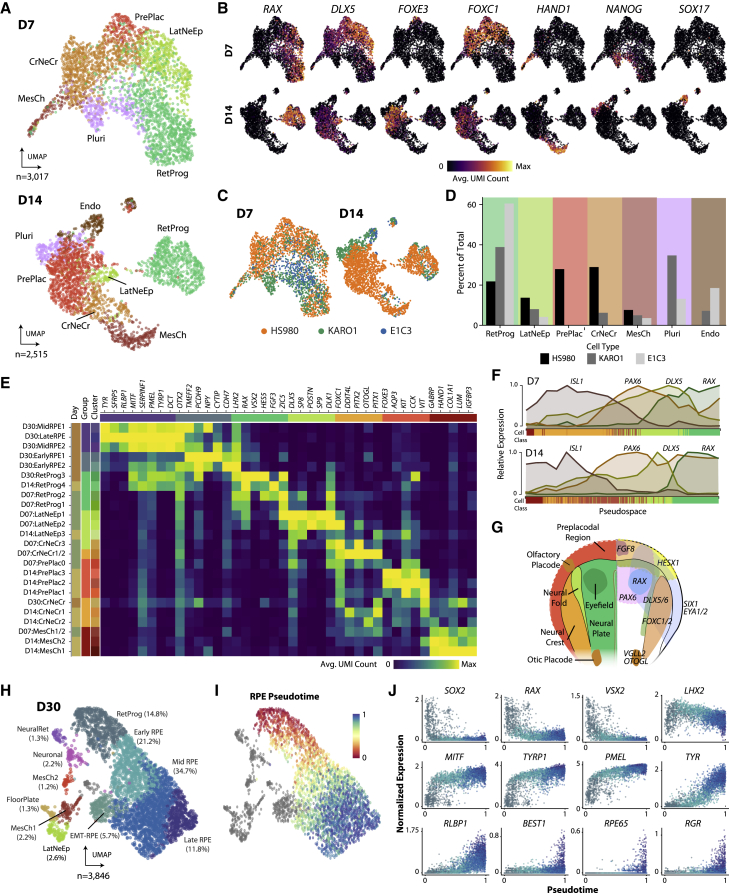
Evaluation of the diverse neuroepithelial cell type derivatives in early hESC-RPE differentiation (A) Uniform manifold approximation and projection (UMAP) at differentiation day 7 (D7) and D14 in three lines. Cells were grouped into retinal progenitor (RetProg), lateral neural fold-like (LatNeEp), pre-placodal-like (Pre-Plac), cranial neural crest-like (CrNeCr), mesenchyme (MesCh), pluripotent (Pluri), and endoderm-like (Endo) clusters. (B) UMAPs showing normalized gene expression of marker genes *RAX* (RetProg), *DLX5* (LatNeEp), *FOXE3* (Pre-Plac), *FOXC1* (CrNeCr), *HAND1* (MesCh), *NANOG* (Pluri), and *SOX17* (Endo). (C) UMAPs in (A) colored by cell line. (D) Bar graphs showing cell type composition in each line at D7 and D14. (E) Enriched gene expression heatmap for HS980 cell types. (F) Plots showing relative expression of neural tube patterning markers in D7 (top) and D14 (bottom) cells across pseudospace in HS980. (G) Schematic of the patterned anterior neural plate at the neurulation stage. Left: putative location of the cell types corresponding to identified clusters. Right: schematic of genes patterning the rostral embryo. (H) UMAP of hESC-RPE differentiation D30 in three lines, colored by cell type. (I) Pseudotime trajectory of all D30 RPE and RetProg cells (82.5% of total at D30). (J) Scatter plots showing progenitor (*SOX2*, *RAX*, *VSX2*, *LHX2*), early (*MITF*, *TYRP1*, *PMEL*), mid (*TYR*, *RLBP1*), and late (*RPE65*, *BEST1*, *TTR*) gene expression along pseudotime. See also Figure S2.

To identify populations during RPE induction, we obtained enriched genes by cluster and cross-referenced the literature, revealing a mixture of intermediate cell states resembling those described in rostral neural tube patterning and eye development (see supplemental experimental procedures). We identified seven groups (pluripotent-like, endodermal-like, lateral neural fold-like, pre-placodal epithelium-like, cranial neural crest-like, mesenchymal, and retinal progenitor) and confirmed these annotations using several literature-supported marker genes (Figures 2A, 2B, and S2A; Table S2; supplemental experimental procedures) (Begbie, 2013; Bosze et al., 2020). This highlighted differences between lines: KARO1 retained pluripotent-like cells at initial time points, HS980 generated more pre-placodal-like cells, and E1C3 initiated an endodermal-like population while also establishing the largest percentage of retinal progenitors (Figures 2C and 2D).

The surprising emergence of different cell types of the anterior ectoderm highlights the inter-relatedness of gene expression programs for the eye, neural crest, and other sensory tissues during early embryonic development. Some secondary clusters matched remarkably well with specific neural tube regions, expressing a combination of markers for eye field (*RAX*, *SIX6*, *LHX2*), telencephalic neural fold (*DLX5*, *DLX6*), lens placodes (*FOXE3*, *PAX6*, *ALDH1A1*), cranial neural crest (*FOXC2*, *VGLL2*, *PITX1*), inner ear placodes (*OTOGL*, *VGLL2*, *CYP26C1*), the anterior neural ridge organizer (*FGF8*, *SP8*, *FOXG1*), and mesenchyme (*GABRP*, *HAND1*, *COL1A1*) (Figure 2E and Table S2; supplemental experimental procedures).

The observation of mesenchyme is interesting because *in vivo* periocular mesenchyme expresses inductive signals that promote RPE fate, a role substituted by Activin A in the protocol. Progenitors (RetProg) detected across differentiation possessed distinct gene expression programs, suggestive of varying degrees of progression toward RPE. RetProg clusters expressed a repertoire of known markers, including *OTX2* and *LHX2*, which are jointly necessary for activation of the transcription factor *MITF*. At D14, these two genes were co-expressed in RetProg clusters alongside MITF-activated genes *PMEL*, *SERPINF1*, *TYRP1*, and *DCT* (Figures 2E and S2B–S2D). Consistent with their classification as progenitors, cells displayed a proliferative signature (S and G_2_/M phases) (Figure S2E). Canonical correlation analysis (CCA) further captured a “pseudospatial” axis of variation, with cells transitioning along a mediolateral molecular profile (Figures 2F and 2G; see supplemental experimental procedures).

To factor out time-dependent differences, we integrated D7 and D14 samples onto a shared feature space, where we observed an increase in cells assigned as pre-placodal and a decrease in both inner ear-like cranial neural crest and lateral neuroepithelial cells (Figures S2F and S2G). These analyses revealed that heterogeneity at both stages recapitulates the molecular profile of rostral embryonic territories patterned to specify sensory organs, such as lens, olfactory, and otic placodes (Begbie, 2013) (Figure 2G).

Conversely, between 79% and 96% of cells at D30, depending on the line, were categorized into retinal progenitor or RPE stages. Other observed cell types included lateral neural fold-like, neuronal, mesenchyme-like cluster, neural retina, and floor plate (Figures 2H and S2H). A pseudotemporal trajectory of retinal maturation largely characterized these cells, confirming a loss of progenitor status (*SOX2*, *RAX*, *VSX2*, *LHX2*), followed by an increase in RPE differentiation (*MITF*, *TYRP1*, *PMEL*) and, later, of advanced RPE maturation markers (*TYR*, *RLBP1*, *RPE65*, *BEST1*, *RGR*) (Figures 2I and 2J). Transcription factor network analysis of D30 HS980 cells with SCENIC confirmed the activity of regulons involving gene targets of *SOX2*, *RAX*, *VSX2*, *OTX2*, and *MITF* (Aibar et al., 2017) (Figure S2I). In summary, this data suggest a “divergence-convergence” model, with an initial expansion of cellular diversity, later dampened to favor the promotion of an RPE differentiation program (Figure S2J).

### 2D hESC-RPE monolayer differentiation is faster and more directed than 3D embryoid body differentiation

The molecular patterning of two-dimensional (2D) cultures during early RPE differentiation hints at an intriguing self-organization process. To clarify how the initial heterogeneity detected in our monolayer differentiation relates to a three-dimensional (3D) protocol that allows cells to organize spatially, we compared it with an embryoid body (EB, HS980 line) differentiation using scRNA-seq (Plaza Reyes et al., 2016).

**Figure 3:**
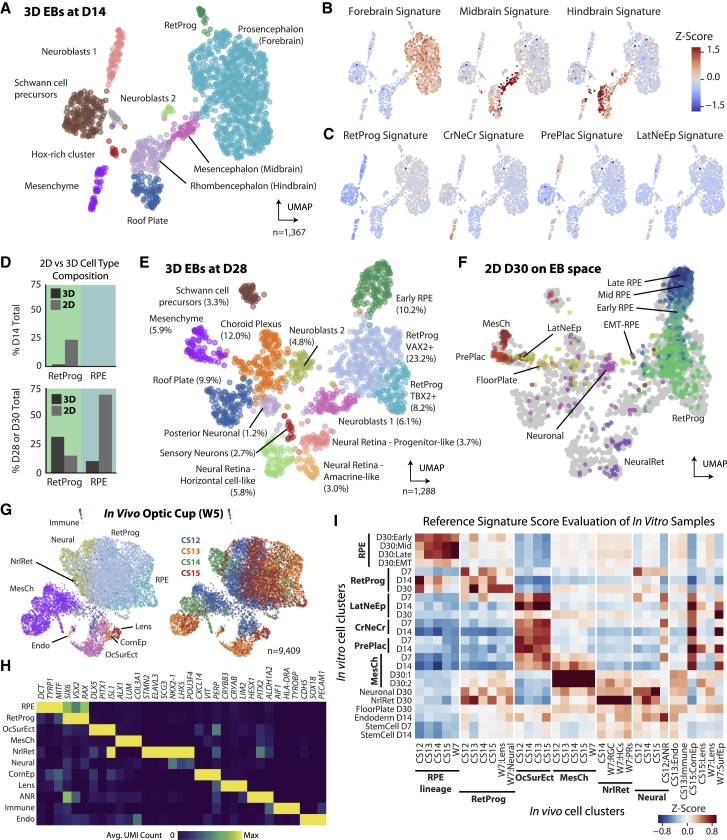
Comparative analysis of RPE induction between hESC-RPE, 3D EB differentiation, and human embryonic eye (A) UMAP of 3D EB cultures at D14 (HS980 line). (B and C) UMAPs showing signature scores for brain regions (B) and neural tube cell types (C) visualized on the EB D14 UMAP. (D) Bar plots comparing cell type compositions in 2D and 3D cultures at D14 (top) and D28/30 (bottom). (E) UMAP of 3D EB cultures at D28 colored by cell type. (F) Projection of 2D D30 cells from all three cell lines onto the UMAP from (E) using pairwise correlation distances, colored by annotated cell type (see supplemental experimental procedures, cf. Figure 2H). Cells in gray are those from (E). (G) UMAP of human embryonic optic cup cells at Carnegie stages 12, 13, 14, and 15 (week 5, W5), colored by cell type (left) or stage (right). (H) Heatmap of enriched gene expression by cell type across all samples in (G). (I) Heatmap showing signature scores of *in vitro* cell clusters at D7, D14, and D30 illustrating the correspondence to *in vivo* clusters from (G). Signature scores were obtained using the top 30 genes of the respective *in vivo* reference population. See also Figure S3.

Initially, EBs displayed uniform patterning with early progenitor and pluripotency markers, but later showed the emergence of clusters corresponding to fore-, mid-, and hindbrain (Figures 3A, 3B, S3A, and S3B). Interestingly, we detected no traces of the more specific mediolateral-patterning signatures observed in the 2D protocol (Figure 3C). Conversely, midbrain and hindbrain gene expression signatures were not detected in the 2D cultures, and a greater fraction of retinal progenitors were observed in the 2D context (24.7% 2D cells compared to 1.6% 3D cells at D14) (Figures 3D and S3C).

EB cultures also continued to harbor more diversity at D28: while 2D cultures were largely defined by various stages of RPE maturation, in the 3D setting other retinal and brain-related cell types were present, including neural retina, caudal neuroblasts, and glial cells (Figures 3D, 3E, and S3D) (Brodie-Kommit et al., 2021; La Manno et al., 2021). This evidence, in conjunction with the presence of distinct *TBX2*^+^ dorsal optic cup-like and *VAX2*^+^ ventral optic cup-/stalk-like progenitor populations, strongly suggests that a wider set of morphogenetic events are recapitulated during the EB protocol (Bosze et al., 2020).

When 2D-cultured cells were projected onto the EB D28 embedding, the majority of 2D cells mapped to RetProg and RPE clusters rather than non-retinal cell types, illustrating that RPE comprises a much larger fraction of the monolayer cultures (73.4% cells) than EB cultures (10.2% cells) (Figures 3E and 3F). 3D differentiation ultimately produces cells of a broader neural origin whereas 2D differentiation induces rostral identity, further funneled to an RPE fate, thus supporting a divergence-convergence model.

### *In vitro* differentiation and eye development exhibit similarities in cellular composition

We reasoned that embryonic references could validate our model and evaluate how faithfully *in vitro* phenotypes match their *in vivo* counterparts. Thus, we performed scRNA-seq of 12,151 cells from four human embryonic optic vesicles from Carnegie stage 12 (CS12), CS13, CS14, and CS15 (approximately 30, 32, 33, and 36 days/5 weeks post-conception) and two eyes at CS20 (7.5 weeks post-conception). Early stages contained patterned cell types corresponding to optic vesicle and surrounding tissues, including retinal progenitors and RPE (Figures 3G and 3H). Retinal tissues were more clearly differentiated into RPE, neural retinal, and optic stalk subpopulations at CS13 than in RPE-focused progenitors detected *in vitro* (Figure S3E, cf. Figures 2E and S2A; Table S3). CS20 samples captured a more diverse representation of cell types surrounding the eye, including proliferating progenitors, RPE, lens, and intermediate retinal ganglion cells (Figures S3F–S3I).

To evaluate the resemblance of hESC-RPE clusters to embryonic references, we extracted enriched genes from *in vivo* cell types at all stages and used them to compute signature scores for *in vitro* cell types at D7, D14, and D30 (Figure 3I). Scores for RPE clusters *in vitro* were highest using enriched genes from the *in vivo* RPEs, with signatures of later RPE populations *in vitro* scoring higher against later-stage embryonic RPEs. An inverse correspondence was observed between *in vivo* RPE signatures and scores for *in vitro* RetProg populations, relating to their gradual maturity. Signatures for *in vivo* ocular surface ectoderm scored highest on the *in vitro* LatNeEp, CrNeCr, and Pre-Plac clusters, which also had overlapping gene expression patterns for *DLX5*, *PITX1*, and *ISL1*, suggesting that these populations are similar to ectodermal tissue surrounding the optic cup (Figure 3H).

### Cell-surface marker NCAM1 defines retinal progenitor cells at D30 of hESC-RPE differentiation

**Figure 4:**
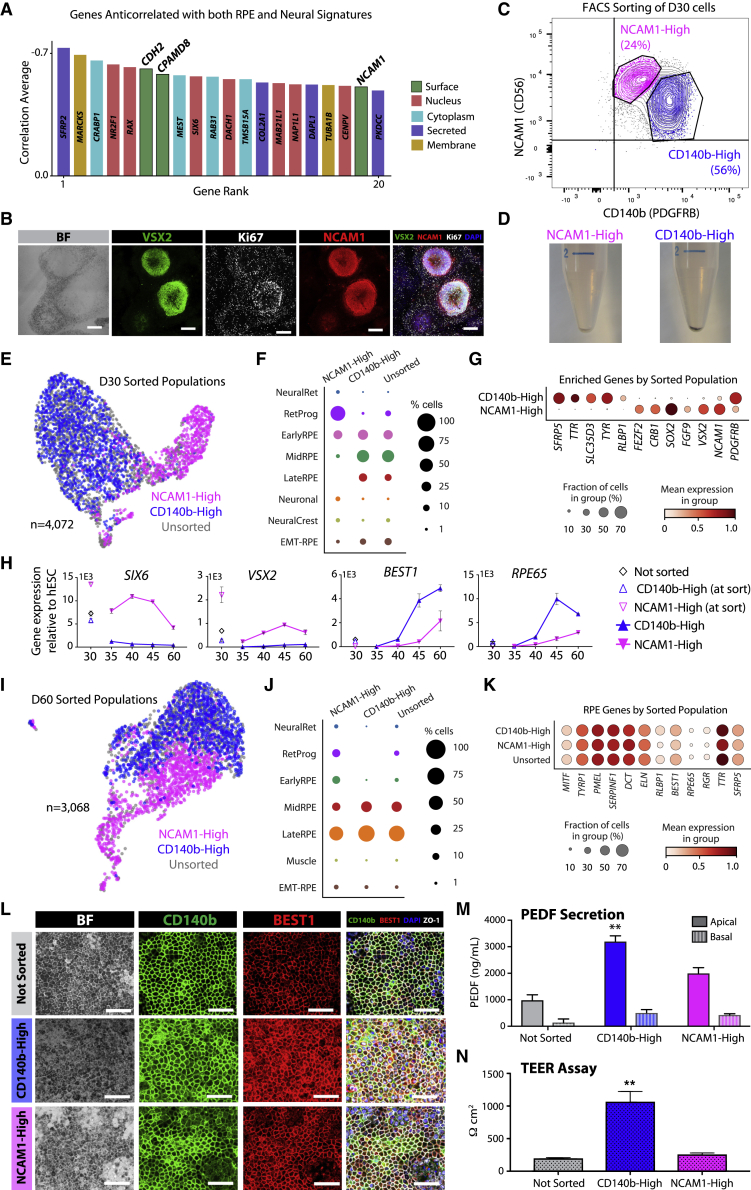
Characterization of the NCAM1-High sorted D30 hESC-RPE population (A) Bar graph of top genes from anticorrelation analysis at HS980 D30. Genes with a mean normalized expression <0.5 were excluded. (B) Brightfield and immunofluorescence stainings of D30 cells showing co-expression of VSX2, NCAM1, and Ki67 markers. Scale bars, 200 μm. (C) Representative FACS plot of NCAM1-CD140b sorting to distinguish distinct populations at D30. Negative gates were set based on fluorescence minus one (FMO) and hESC control samples. (D) Post-sort pellets of CD140b-High and NCAM1-High cells. (E) UMAP of NCAM1-High (pink), CD140b-High (blue), and unsorted (gray) D30 cells after CCA integration. (F) Dot plot illustrating the proportion of cells corresponding to each identified cell type in scRNA-seq samples from (E). (G) Dot plot of selected progenitor (*FEZF2*, *CRB1*, *SOX2*, *FGF9*, *VSX2*) and RPE (*SFRP5*, *TTR*, *SLC35D3*, *TYR*, *RLBP1*) genes enriched in the sorted samples. (H) Graphs showing qRT-PCR of retinal progenitor (*SIX6*, *VSX2*) and RPE (*BEST1*, *RPE65*) marker genes in populations from (E) at the moment of sort and at post-sort D30, D35, D40, D45, and D60. (I) UMAP of NCAM1-High (pink), CD140b-High (blue), and unsorted (gray) D60 cells. (J) Dot plot illustrating the proportion of cells corresponding to each identified cell type in scRNA-seq samples from (I). (K) Dot plots of early (*MITF*, *TYRP1*, *PMEL*, *SERPINF1*, *DCT*, *ELN*) and late (*RLBP1*, *BEST1*, *RPE65*, *RGR*, *TTR*, *SFRP5*) RPE genes in the LateRPE cell clusters from each sorted sample. (L) Brightfield and immunofluorescence stainings of unsorted, CD140b-High, and NCAM1-High populations 30 days after sorting (D60) showing co-expression of CD140b, BEST1, and ZO-1 markers. Scale bars, 100 μm. (M and N) Bar graphs showing PEDF secretion (M) and TEER measurements (N) of the unsorted, CD140b-High, and NCAM1-High populations at D60. ∗∗p < 0.0001 compared to Not Sorted and NCAM1-High. In (H), (M), and (N), error bars represent mean ± SEM from three independent experiments. See also Figure S3.

We next aimed to identify cell-surface markers that could distinguish retinal progenitors from RPE cells. We computed a Pearson’s correlation coefficient between highly expressed genes at D30 and RPE or neural tube markers (Figures S4A and S4B). Genes with strong anticorrelation to both signatures included transcription factors involved in retina development (*SFRP2*, *CRABP1*, *RAX*, *SIX6*) as well as genes implicated in neural tube (*CPAMD8*, *PKDCC*, *NR2F1*) and lens (*MARCKS*, *DACH1*, *MAB21L1*) development (Figure 4A; see supplemental experimental procedures).

Cell-surface markers *CDH2*, *CPAMD8*, and *NCAM1* were among the most prominent progenitor markers at D30. NCAM1 staining areas coincided with rosette structures lacking pigmentation, and both scRNA-seq and protein staining revealed that NCAM1 was co-expressed with progenitor (VSX2 and RAX) and proliferative (Ki67) markers at D30 (Figures 4B and S4C–S4E).

To functionally examine whether NCAM1-positive cells hold potential to generate RPE cells, we sorted using NCAM1 and CD140b (PDGFRB), an RPE cell marker (Plaza Reyes et al., 2020a) (Figure 4C). Consistently, pigmentation was evident in the CD140b-High population cell pellets, whereas the NCAM1-High population lacked pigmentation (Figure 4D). Transcriptionally, we found that NCAM1-High cells predominantly corresponded to RetProg (64.7%) and EarlyRPE (20.6%), whereas CD140b-High cells were mostly of EarlyRPE (28.0%), MidRPE (42.9%), and LateRPE (17.6%) profiles (Figures 4E–4G, S4F, and S4G).

We then differentiated sorted cells for 30 additional days. Morphological evaluation showed that CD140b-High cells already generated a homogeneous hESC-RPE monolayer at D45, while NCAM1-High cells only yielded a defined RPE morphology at D60 (with cobblestone scores of 6.77e−3 per μm^2^ for the CD140b-High population, and 5.29e−3 per μm^2^ for the NCAM1-High population at D60) (Figure S4H, cf. Figure S1A). NCAM1-High cells were positive for the proliferation marker Ki67 and retinal progenitor transcription factor VSX2 (Figures S4I and S4J). These patterns were further confirmed by qRT-PCR (Figures 4H and S4K).

Establishment of an RPE phenotype at D60 by both NCAM1-High and CD140b-High populations was confirmed by scRNA-seq and immunofluorescence (Figures 4I–4L, S4L, and -S4M). Functionally, we assessed pigment epithelium-derived factor (PEDF) secretion and transepithelial resistance (TEER) upon protocol completion (D60), finding that CD140b-High-derived cells secreted significantly higher apical levels of PEDF than the unsorted and NCAM1-High-derived cells (Figure 4M). TEER levels displayed by CD140b-High-derived cells were higher compared to unsorted and NCAM1-High populations (Figure 4N). These results show that NCAM1 captures an immature progenitor population with potential to mature into functional RPE cells.

### NCAM1-High cells can differentiate into alternative retinal cell types

**Figure 5:**
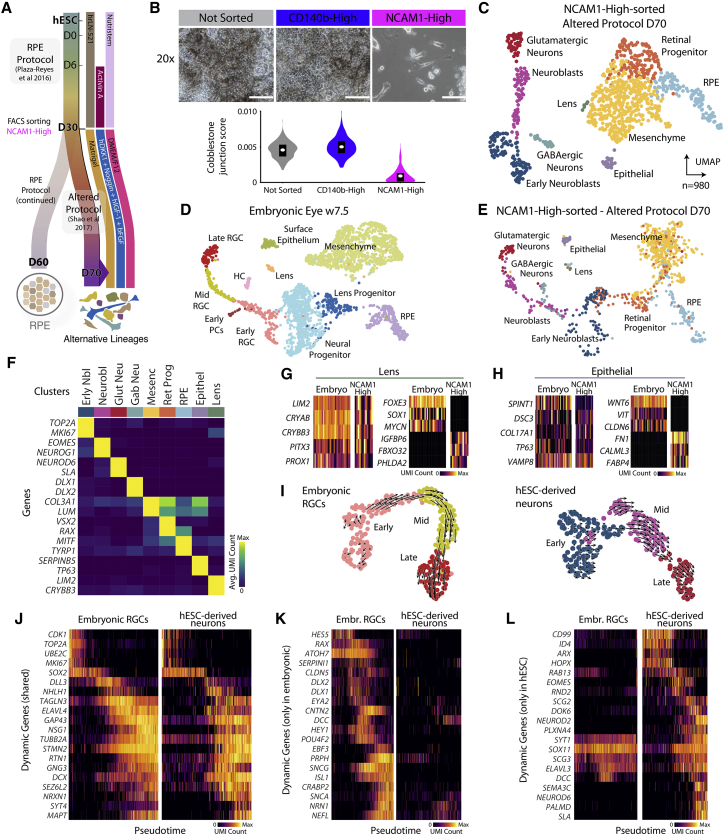
Neuroretinal progenitor differentiation of NCAM1-High-sorted hESC-RPE D30 cells (A) Schematic of the neuroretinal progenitor (altered) differentiation protocol (HS980 line). D30 NCAM1-High-sorted cells were sorted and replated on Matrigel containing DMEM/F12, hDKK1, Noggin, hIGF-1, and bFGF until scRNA-seq at D70. (B) Brightfield images and cobblestone junction scores of sorted and unsorted populations at D70. Scale bars, 100 μm. (C) UMAP of NCAM1-High sorted cells at D70. (D and E) CCA integration of scRNA-seq data from embryonic week 7.5 eye (D) and NCAM1-High-sorted cells subjected to the altered protocol (E). (F) Heatmap of enriched gene expression for cell types in (C). (G and H) Gene expression heatmaps of lens (G) and epithelial (H) cells identified in the reference and *in vitro*. Shared and differentially expressed genes are shown on the left and right plots, respectively. (I) RNA velocity of embryonic retinal ganglion cells (left) and hESC-derived neurons (right). (J–L) Heatmaps showing gene expression analysis of embryonic and hESC-derived neurons along their respective pseudotimes. RGC, retinal ganglion cell; PC, photoreceptor cell; HC, horizontal cell.

To further evaluate the differentiation potential of D30 NCAM1-High cells, we plated sorted cells in neuroretinal promoting conditions for 40 additional days (Shao et al., 2017) (HS980 line, Figure 5A). NCAM1-High cells gave rise to a heterogeneous culture with a significant portion of cells displaying a distinct non-RPE cell body morphology, unlike CD140b-High cells under the same conditions (Figure 5B). Transcriptional profiling showed that only 12% of the analyzed cells were RPE, suggesting that NCAM1-High cells at D30 represent an uncommitted progenitor with potential beyond RPE whereas CD140b-High captures lineage-committed RPE cells (Figure 5C).

To systematically compare NCAM1-High-derived cells to a developmental reference, we performed CCA with week 7.5/CS20 embryonic eyes. The shared low-dimensional space emphasized similarities between corresponding clusters, including RPE, progenitor, lens, surface epithelial, and neuronal populations (Figures 5D–5H). Moreover, there was an overlap between week 7.5 retinal ganglion and NCAM1-High-derived neurons (Figures 5D and 5E, cf. Figure S3). To compare gene expression dynamics, we computed RNA velocity on each neuronal population, revealing progression toward a more mature state (La Manno et al., 2018) (Figure 5I). Pseudotemporal gene expression confirmed a common profile of expression waves, with gradual downregulation of proliferation markers (*TOP2A*, *MKI67*) followed by upregulation of a neuronal differentiation program (*STMN2*, *TUBB2A*, *DCX*) (Figure 5J). However, critical markers of retinal ganglion development, such as transcription factor *ATOH7* and its downstream targets *POU4F2* and *ISL1*, were only expressed in the week 7.5/CS20 cells (Gao et al., 2014) (Figure 5K). Other neuronal markers (*EOMES*, *NEUROD2*, *SLA*) were unique to NCAM1-High-derived neurons, implying that NCAM1-High-derived cells are another type of telencephalic neuron (Figure 5L). NCAM1-High cells are thus either a mixed pool of retinal and neuroepithelial progenitors capable of forming both cell types and other related retinal lineages or cells with the capacity to establish all of these lineages.

### Late differentiation is characterized by the selection and maturation of RPE populations

**Figure 6:**
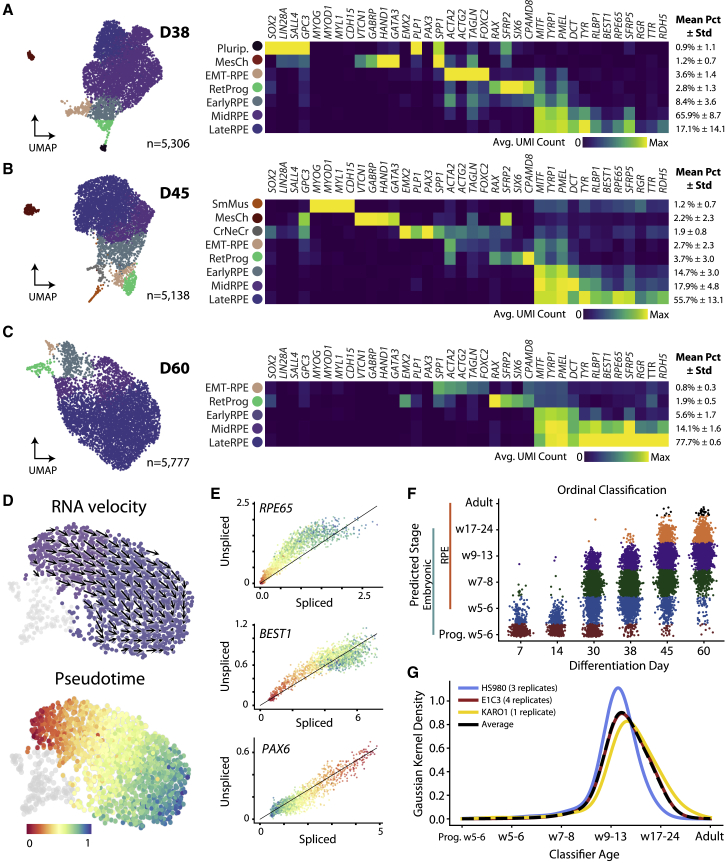
Late hESC-RPE differentiation profiling (A–C) UMAPs and enriched gene expression heatmaps of hESC-RPE scRNA-seq data at D38 (A), D45 (B), and D60 (C) in all three lines. (D) RNA velocity and pseudotime analysis of HS980 RPE at D60. (E) Phase portraits of upregulated RPE marker genes *RPE65* and *BEST1* as well as a downregulated progenitor marker *PAX6*. The diagonal line represents the estimated steady state of gene expression, with cells above the steady state experiencing gene upregulation and those below gene downregulation. (F) Plot showing ordinal classification of 20,682 single hESC-derived retinal progenitor and RPE cells at six differentiation time points along embryonic stages. (G) Graph representing classification distribution for seven hESC-RPE differentiation D60 biological replicates (HS980: 3,655 cells; E1C3: 61,479 cells; KARO1: 1,236 cells). See also Figure S6.

Unlike initial stages, scRNA-seq of three time points after replating revealed that most cells were of an RPE state: the proportion of LateRPE cells was 17.1% at D38, 55.7% at D45, and 77.7% at D60. By D60, approximately 98.2% of cells were of some RPE identity, with the remaining fraction consisting of retinal progenitors. At intermediate D38 and D45, small fractions of non-retinal mesenchyme and smooth muscle contaminants were detected. However, these populations were no longer present in culture at D60 (Figures 6A–6C and Table S5). We also detected a distinct cluster of lingering pluripotent cells in the HS980 D38 sample (0.9% of cells) expressing pluripotency markers *SOX2*, *LIN28A*, *SALL4*, and *GPC3* (Figure 6A). As such cells must be eliminated from the final cell product, we extended our analysis to include eight independent D60 samples containing 63,370 cells across all three lines. Encouragingly, not a single cell with a pluripotent signature was detected in the D60 samples (Figures S5A, S5B, S1H, and S1I).

D38 cells also displayed an increased heterogeneity and on average showed a less distinct RPE cobblestone morphology than D30 (Figures 1C and S1A). Furthermore, from D30 a cell population co-expressed *MITF* and markers associated with the epithelial-to-mesenchymal (EMT) transition process, particularly *ACTA2*. The dissociation of RPE cells at D30 likely induced a mesenchymal-like morphology of the RPE (Figure 1B). This observation led to characterization of two early RPE clusters from D30 onward, one *MITF*^+^*ACTA2*^−^ (EarlyRPE) and one *MITF*^+^*ACTA2*^+^ (EMT-RPE) (Figure S5C). EMT-RPE expressed some but not all RPE markers while co-expressing EMT genes. The fraction of EMT-RPE increased during replating from D30 to D38, followed by a steady decrease to low levels (0.8% of cells) by D60 (HS980). Moreover, the representation of RPE from later time points along a phenotype variation axis confirmed the presence of shared EMT and RPE differentiation properties (Figures S5C–S5F).

Nonetheless, after D30 we observed the persistence of RPE and loss of other cell types; pseudotime inference and RNA velocity showed a trajectory of less mature populations in gene expression space toward the most mature RPE (HS980) (Figure 6D). Phase portrait analysis comparing the steady-state expectations for spliced and unspliced RNA levels further confirmed the upregulation of *RPE65* and *BEST1* as well as the downregulation of progenitor marker *PAX6* (Figure 6E; see supplemental experimental procedures).

### Replating affects cell population composition and promotes a purer and more mature cell product

We previously showed that replating D30 monolayer cultures facilitates the expansion of final cell numbers (Plaza Reyes et al., 2020a), but we had not explored how replating affects maturation and purity. We therefore repeated our differentiation protocol without the replating step and performed scRNA-seq, revealing significant contamination with cells resembling neural retina, neuronal, lens, mesenchyme, neural crest, and mesoendoderm (Figures S5G and S5H). The presence of contaminant types was confirmed by flow cytometry for lack of the RPE marker CD140b (Figure S5I). Non-replated cultures at D60 maintained more RetProg cells and fewer LateRPE cells (Figures S5H and 6C). We determined that replating selects against retinal progenitors and arrests the expansion of alternative lineages. This was paralleled by increased RPE cobblestone morphology in D60 replated cultures compared to non-replated counterparts (Figure S5J).

To assess overall progression of hESC-RPE and assign cells to developmental stages, we constructed an ordinal classifier using transcriptomes of 783 embryonic eye cells from week 5 to week 24 and 127 adult RPE cells (Hu et al., 2019; Voigt et al., 2019) (Figures S6Aand S6B). As proof of principle, we applied the classifier to CS13 and CS20 embryonic references and to an independent set of 49 adult RPEs, confirming their appropriate assignment (Quake and Sapiens Consortium, 2021) (Figures S6C). Evaluation of the developmental maturity confirmed that replating leads to a more mature output (Figure S5K). Furthermore, classification of the maturation status for all *in vitro* retinal progenitor and RPE cells confirmed a gradual progression throughout differentiation corresponding to embryonic RPE development; the classifier also assigned a consistent maturation level among all D60 lines and replicates (Figures 6F, 6G, and S6D).

Lastly, we compared the maturation of RPE cells from our D60 monolayer protocol to RPE cells generated through 3D EB differentiation and to those from other protocols with a longer differentiation. D60 RPE cells in either 3D or 2D differentiation showed similar maturation statuses (Figure S6H). In addition, reanalysis and classification of scRNA-seq data in which differentiation was performed for 95 or 432 days using another 2D monolayer protocol showed that our D60 cells are similar in maturation status to the D95 cells, but that further maturation can be achieved through extensive *in vitro* culturing (Lidgerwood et al., 2021) (Figures S6E–S6H). Interestingly, while 95- and 432-day time points contained highly mature RPE, both samples also included fractions of retinal progenitors and EMT-RPE, as also observed in D60 samples (Figures S6G and S6H).

### Subretinal transplantation of hESC-RPE facilitates a more advanced RPE state

**Figure 7:**
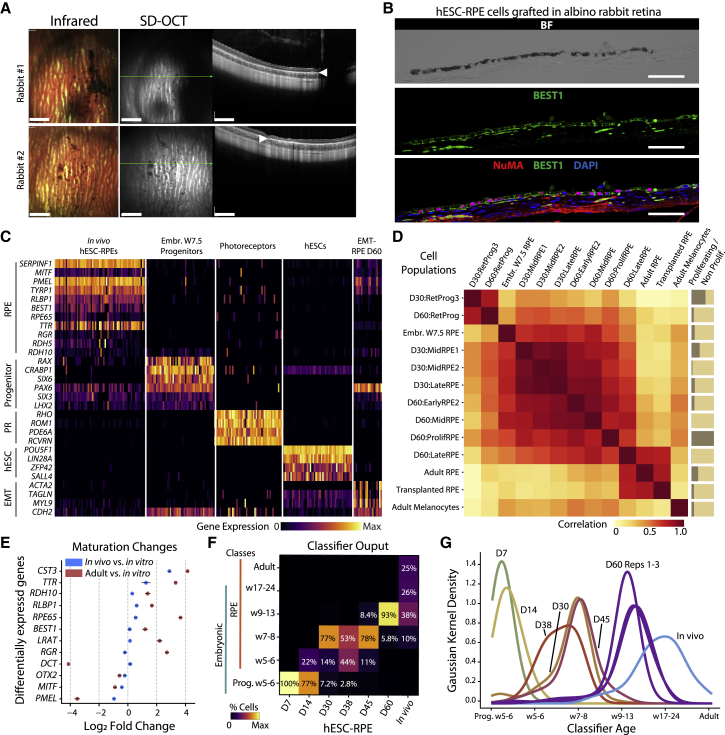
Phenotyping of hESC-RPE transplanted in the albino rabbit subretinal space (A) Infrared and SD-OCT images of injected hESC-RPE cells (HS980 line) in the subretinal space of albino rabbits. Green lines indicate the SD-OCT scan plane. White arrows indicate the hyper-reflective RPE layer. Scale bars, 1 mm. (B) Brightfield and immunofluorescent staining for human marker NuMA and BEST1 30 days after injection. Scale bars, 50 μm. (C) Gene expression heatmap comparing 65 single hESC-RPE cells 30 days after transplantation to embryonic week 7.5 retinal progenitors, adult photoreceptors, undifferentiated hESCs, and D60 EMT-RPE. (D) Pearson’s correlation matrix between gene expression profiles of HS980 hESC-RPEs at D30 and D60, post-transplantation (*in vivo*) RPE, adult RPE and melanocytes, and embryonic RPE. (E) Dot plot graph showing log_2_ fold change of RPE markers between HS980 hESC-RPE D60 cells, *in vivo* RPE, and adult RPE. Error bars represent mean ± SEM from all cells at each time point. (F) Ordinal classification summary matrix showing the percentage of HS980 retinal cells from *in vitro* and *in vivo* time points predicted to correspond to each RPE developmental time point (embryonic weeks 5–24, adult). (G) Graph showing classification distribution for hESC-derived progenitor and RPE cells *in vitro* and *in vivo*. See also Figure S7.

Given the therapeutic potential of hESC-derived RPE, we next wanted to investigate the transcriptional profiles of cells following *in vivo* transplantation to assess whether cells continued to mature and ensure that alternative lineages remain absent. D60 hESC-RPE cells (HS908 line) were therefore transplanted into the subretinal space of two albino rabbits, a preclinical large-eyed animal model with an assessed high degree of transcriptional similarity (Figure S7 and Table S6). Four weeks following transplantation, infrared and spectral domain optical coherence tomography (SD-OCT) imaging showed pigmented patches of hyper-reflective RPE layer in the albino rabbit retina (Figure 7A). Histology and immunofluorescence analysis of 227 human-NuMA^+^ single cells found that 99.56% were either pigmented or expressed the RPE marker BEST1, thus corroborating the successful integration of injected hESC-RPE cells in a polarized and matured RPE monolayer (Figures 7B, S6I, and S6J). The contiguous injected retina of two rabbits was then processed for scRNA-seq, yielding 65 human hESC-derived cell profiles that all exhibited a high expression of mature RPE markers. Crucially, markers of retinal progenitors, photoreceptors, pluripotent hESCs, and EMT-RPE were benchmarked against our references and undetected *in vivo* (Figure 7C).

Gene expression correlation analysis of grafted cells to embryonic references and *in vitro* clusters from D30 and D60 confirmed similar patterns overall, yet one *in vitro* cluster (D60: LateRPE) and the *in vivo* transplanted RPE were the most similar to the adult RPE reference (Figure 7D, cf. Figures 3, S3, and S6). Differential expression analysis confirmed an expression pattern closer to adult RPE cells after *in vivo* implantation, particularly for visual cycle components (Figure 7E). Interestingly, ordinal classification of the retrieved 65 post-transplantation human cells showed that the gradual progression of maturity *in vitro* continued further in the *in vivo* environment. Transplanted hESC-RPE were assigned into the late embryonic weeks 17–24 (26%) and adult RPE ordinal classes (25%) more than any *in vitro* time point, whereas D60 cells were predominantly assigned to embryonic weeks 9–13 (93%) (Figures 7F and 7G).

## Discussion

By molecular profiling an hESC-RPE differentiation protocol established for clinical translation, we demonstrate that the described culture conditions successfully specify RPE lineage, selection, and maturation over 60 days through a sequence of gene expression waves consistent with embryological studies (Fuhrmann et al., 2014; Hu et al., 2019). At early stages, we found cell pool heterogeneity incompatible with the induction of a single lineage and, instead, evidence of widespread initial cellular diversity. Similar heterogeneity expansion was observed in studies of endoderm and endothelial tissue derivation, but meta-analysis of several differentiation protocols is needed to understand whether the observed event is a widespread phenomenon (Cuomo et al., 2020). Our findings suggest a divergence-convergence model: heterogeneity expansion at early time points (influenced by cell-line-specific properties), followed by selection of RPE lineage (driven by replating at D30), and convergence onto a homogeneous and highly pure cellular product.

Each cell line manifested different biases to this initial diversification: E1C3 developed endoderm-like cells, HS980 produced populations reminiscent of different rostral neural tissues, and a fraction of KARO1 displayed signatures of lingering pluripotent cells at the earliest time points. Particularly interesting is the finding of expression profiles resembling patterned regions surrounding the optic field: the pre-placodal epithelium, neural fold, and neural crest. This axis of embryonic patterning is induced by organizer cells of the floor plate and anterior neural ridge, which promote specification of different anterior neural tube territories, including the optic vesicle (Begbie, 2013; Streit, 2007). These findings hint at an intriguing self-organization process occurring in 2D culture, despite the lack of spatially directed cues or 3D structure.

Contrasting with embryonic and adult references, our data highlight that an adult RPE pattern of expression is not yet reached in D60 cells. While a more mature stage can be achieved after 1 year of further culturing, even such long-term RPE cultures still contain persisting retinal progenitor and EMT-RPE populations (Lidgerwood et al., 2021). Further studies are warranted to elucidate the function of these populations to understand whether they may represent a normal part of RPE physiology and how they may impact cell therapy products.

We demonstrated that NCAM1-High cells are not RPE-fate restricted and, upon altered culture conditions, can give rise to additional cell types including anterior neurons, mesenchyme, and lens epithelium. This potency is particularly relevant, as the identification and isolation of less mature progenitors with an increased plasticity is of importance to efforts aimed at replacement of other retinal cell types affected by advanced AMD (Bhatia et al., 2010; Marquardt et al., 2001). Further evaluation of the NCAM1-High potency as a response to different and more specific culture conditions, and in other *in vivo* models lacking certain retinal cell types, constitute promising avenues for future investigation.

Considering that the final cell product may contain 2% of such retinal progenitors, it should be averted in the future despite being unlikely to pose a safety risk. However, we did not detect any non-RPE cell types from our histological (227 cells) or transcriptional (65 cells) analysis following cell transplantation, suggesting that the progenitor pool has not expanded or generated alternative lineages. Additionally, our analysis highlights the importance of ensuring that the final cell product does not contain lingering PSCs at a single-cell level, especially as we detected unexpected pluripotent signatures as late as D38. Importantly, our focused analysis showed that at D60, none of the eight samples from the three cell lines (63,370 cells) contained cells with a pluripotent profile.

The behavior of grafted cells *in vivo* is a topic discussed extensively by the community, with maintenance of the proliferative potential and dedifferentiation generally considered the two processes of major concern (Wang et al., 2020; Zarbin et al., 2019). Our analysis identified neither specific signs of dedifferentiation nor the presence of a non-RPE molecular profile. Instead, we detected a distinct shift in the RPE maturation toward a more adult phenotype. The induction mechanism of the observed *in vivo* maturation remains unclear, albeit the increased expression of visual cycle genes suggests that grafted hESC-RPE cells support neighboring photoreceptors functionally.

Overall, our findings provide a high-resolution perspective on hPSC differentiation and a necessary detailed analysis of a stem cell-based product intended for successful and safe human therapeutic strategies. Ultimately, this study will guide future efforts focused on the differentiation of retinal cells, leading to a deeper understanding of mechanisms of retinal disease and applications in regenerative medicine.

## Experimental procedures

### hESC cell culture and hESC-RPE differentiation

hESC lines HS980 and KARO1 were established and cultured in 5% CO_2_/5% O_2_ on rhLN-521 (10 μg/mL) and passaged as described previously (Rodin et al., 2014). E1C3 (NN GMP0050E1C3) cultured on iMatrix-511 (0.25 μg/cm^2^, Nippi, T303) was provided as a research cell bank of the clinical GMP cell line by NovoNordisk (UCSF IRB: 1518222, for RPE differentiation Projekt-ID: H-18016740, Anmeldelsesnr.: 73105).

For differentiation (Plaza Reyes et al., 2020a, 2020b), cells were plated at a density of 2.4 × 10^4^ cells/cm^2^ on 20 μg/mL hrLN-521 or iMatrix-coated dishes using NutriStem hPSC XF medium and Rho-kinase inhibitor (10 μM) during the first 24 h. Medium was then replaced with NutriStem hPSC XF without basic fibroblast growth factor (bFGF) and transforming growth factor β (differentiation medium) in 5% CO_2_/21% O_2_, and from day 6 after plating, 100 ng/mL of Activin A was added to the medium for a total of 30 days. Day-30 monolayers were replated using TrypLE Select (10 min, 37°C) and passed through a 40-μm strainer. Cells were seeded on hrLN-521-coated dishes (20 μg/mL) at 6.8 × 10^4^ cells/cm^2^, and fed three times a week for 30 subsequent days with differentiation medium without Activin A.

### Sample processing for single-cell RNA sequencing

For cells, specific stage hESC-RPE cells were trypsinized with TrypLE (10 min, 37°C, 5% CO_2_) and resuspended to 1,000 cells/μL in 0.04% BSA in PBS prior to scRNA-seq.

For tissues, two human 32-h postmortem eyes from the same donor were collected; the retinas were dissected out and cut into several small pieces mixed together in 500 μL of digestion buffer (see supplemental experimental procedures). Two pooled embryonic eyes at Carnegie stages 12, 13, 14, and 15 (5 weeks post-conception) and two embryonic eyes from the same donor (7.5 weeks post-conception) were collected. Optic cups were dissected out and chopped in several small pieces to facilitate dissociation in 500 μL of digestion buffer. Two rabbit eyes (from different animals) with 30-day integrated hESC-RPE were enucleated and neuroretina, choroid, and RPE layers were dissected out and mixed together in 500 μL of digestion buffer. After digestion (37°C, 25 min on a 300 × *g* rotator, resuspended every 5 min), samples were filtered using a 30-μm strainer followed by a Dead Cell Removal kit. At this stage, one of the rabbit eye cell samples was stained with mouse anti-human HLA-ABC-FITC; HLA-ABC-positive cells were sorted by fluorescence-activated cell sorting (FACS), collected, and resuspended to 1,000 cells/μL in 1% BSA in PBS. The rest of the samples were also resuspended to 1,000 cells/μL in 1% BSA in PBS prior to scRNA-seq.

### Single-cell RNA sequencing analysis

Cells were either transported at 4°C to the Eukaryotic Single Cell Genomics Facility (ESCG; SciLifeLab, Stockholm, Sweden) or used in-house to prepare cDNA libraries for scRNA-seq. The 10x Genomics Single Cell 3′ Reagent Dual Index Kit v2 and v3.1 (10x Genomics, CG000315) was used, sometimes with an additional Cell Multiplexing Oligo Labeling step (10x Genomics, CG000391), followed by protocol CB000388 and sequencing on a NovaSeq 6000 (ESCG) or Illumina Nextseq 2000 (in-house). Cell Ranger 3.1.0 was used to convert base call files to FASTQ format, map sequencing reads to the human GRCh38 reference transcriptome, and generate feature-barcode matrices. For the E1C3 cell line sequenced at NovoNordisk, CellRanger 3.0.2 was used. Quality control, normalization, dimensionality reduction, and visualization were performed using the *scanpy* and *velocyto* modules (La Manno et al., 2018; Wolf et al., 2018). For samples on which RNA velocity was performed, the *velocyto run10x* command was used on CellRanger sorted BAM files to produce loom files containing spliced and unspliced counts. Cell filtering, dimensionality reduction, and visualization criteria are provided for each individual sample in supplemental experimental procedures and Table S1.

### Subretinal transplantation and *in vivo* imaging

Dissociated hESC-RPEs were injected in sterile PBS (50 μL; 50,000 cells) subretinally using a transvitreal pars plana technique in New Zealand white albino rabbits (Bartuma et al., 2015; Petrus-Reurer et al., 2017, 2018). SD-OCT and confocal scanning laser ophthalmoscopy was performed to obtain horizontal cross-sectional B-scans and *en face* fundus *in vivo* images, respectively.

### Data and code availability

FASTQ files, processed feature-barcode count matrices, annotated h5ad/loom files, and other metadata are available on the Gene Expression Omnibus (GEO: GSE164092). Jupyter notebooks for the single-cell analyses are shared at https://github.com/lamanno-epfl/rpe_differentiation_profiling_code. Datasets are available for interactive visualization and analysis at https://asap.epfl.ch/ under public keys ASAP 75–90 (David et al., 2020).

## Author contributions

S.P.-R., A.R.L., F.L., and G.L.M. conceived the study; F.L., G.L.M., and J.C.V. supervised the work; S.P.-R., L.B.-V., I.K., M.W., H.A., E.S., A.B., A.W., Y.S., P.E., A. Kriegstein, and A. Kvanta performed experiments; I.D. and B.P. helped with the cell sorting; H.B. and M.A. contributed to the animal work; A.R.L., E.J., H.W., and G.L.M. performed scRNA-seq analysis; S.P.-R., A.R.L., A.K., G.L.M., and F.L. planned experiments, analyzed data, and wrote the manuscript.

## Conflicts of interest

S.P.-R., and F.L. are the inventors of a patent (“Methods and compositions for producing retinal pigment epithelium cells,” filed 19.06.2019, PCT/EP2019/066285).

## Supplemental information

Document S1. Figures S1–S7 and supplemental experimental procedures

Table S1. Overview of all scRNA-seq samples generated and quality control conditions, related to experimental procedures

Table S2. Gene enrichment during hESC-RPE pigmentation induction (D7, D14, and D30) by cell type and time point, related to Figure 2

Table S3. Gene enrichment for embryonic eyes at Carnegie stages 12, 13, 14, 15, and 20 by cell type and time point, related to Figure 3

Table S4. Gene correlation scores with retinal progenitor and neural tube signature during the identification of NCAM1 cell-surface marker, related to Figure 4

Table S5. Gene enrichment during late hESC-RPE differentiation (D38, D45, D60) by cell type, related to Figure 6

Table S6. Gene enrichment scores for adult human and rabbit eyes by cell type, related to Figure S7

Document S2. Article plus supplemental information
