# Supplementary material for: Molecular profiling of stem cell-derived retinal pigment epithelial cell differentiation established for clinical translation
Source: Stem Cell Reports. 2022 Jun 14;17(6):1458–75. doi: 10.1016/j.stemcr.2022.05.005 (PMC9214069; doi:10.1016/j.stemcr.2022.05.005)
Supplement: Document S1. Figures S1–S7 and supplemental experimental procedures [file mmc1.pdf]

Stem Cell Reports, Volume *17*

Supplemental Information

Molecular profiling of stem cell-derived retinal pigment epithelial cell differentiation established for clinical translation

Sandra Petrus-Reurer, Alex R. Lederer, Laura Baqué-Vidal, Iyadh Douagi, Belinda Pannagel, Irina Khven, Monica Aronsson, Hammurabi Bartuma, Magdalena Wagner, Andreas Wrona, Paschalis Efstathopoulos, Elham Jaberi, Hanni Willenbrock, Yutaka Shimizu, J. Carlos Villaescusa, Helder André, Erik Sundström, Aparna Bhaduri, Arnold Kriegstein, Anders Kvanta, Gioele La Manno, and Fredrik Lanner

SUPPLEMENTAL ITEMS

Molecular profiling of stem cell-derived retinal pigment epithelial cell differentiation established for clinical translation

Petrus-Reurer, Lederer et al.

Supplemental Figures 1 – 7

Supplemental Tables 1 – 6

Experimental Procedures

SUPPLEMENTAL FIGURES

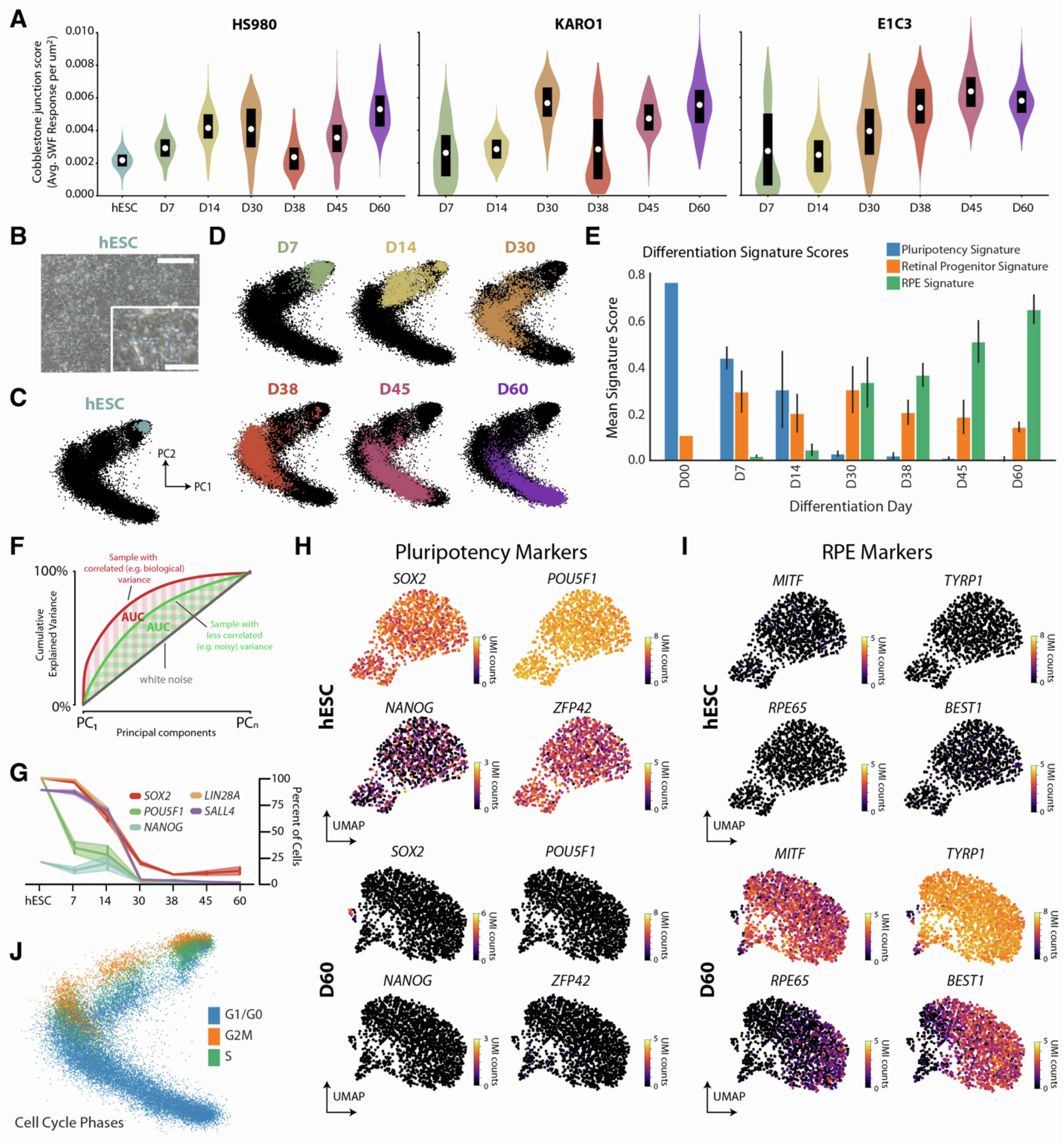

Figure S1. Cellular heterogeneity analysis of hESC-RPE differentiation. Related to Figure 1. (A) Graphs showing quantification of cobblestone morphology throughout differentiation in the HS980, KARO1, and E1C3 cell lines using the junction score methodology and software developed by Joshi et al., 2016. **(B)** Brightfield image of undifferentiated hESCs in the HS980 line. Scale bars: 100μm; inset 20μm. **(C)** Principal component (PC) representation of hESCs in the HS980 line. **(D)** PC representation of *in vitro* hESC-RPE time points across three lines, colored by day. **(E)** Bar graph of average pluripotency, retinal progenitor and RPE signature scores by differentiation day. Error bars represent standard deviation of the mean over three cell line replicates. **(F)** Schematic of AUC variance evaluation metric. **(G)** Graph showing the percentage of cells positive (>0.5 normalized UMI counts) for pluripotency marker genes at each time point. **(H, I)** UMAPs showing normalized gene expression of pluripotent stem cells markers (H) and RPE markers (I) in undifferentiated hESCs and at D60. **(J)** Principal component representation of hESC-RPE differentiation across all lines colored by assigned cell cycle phase.

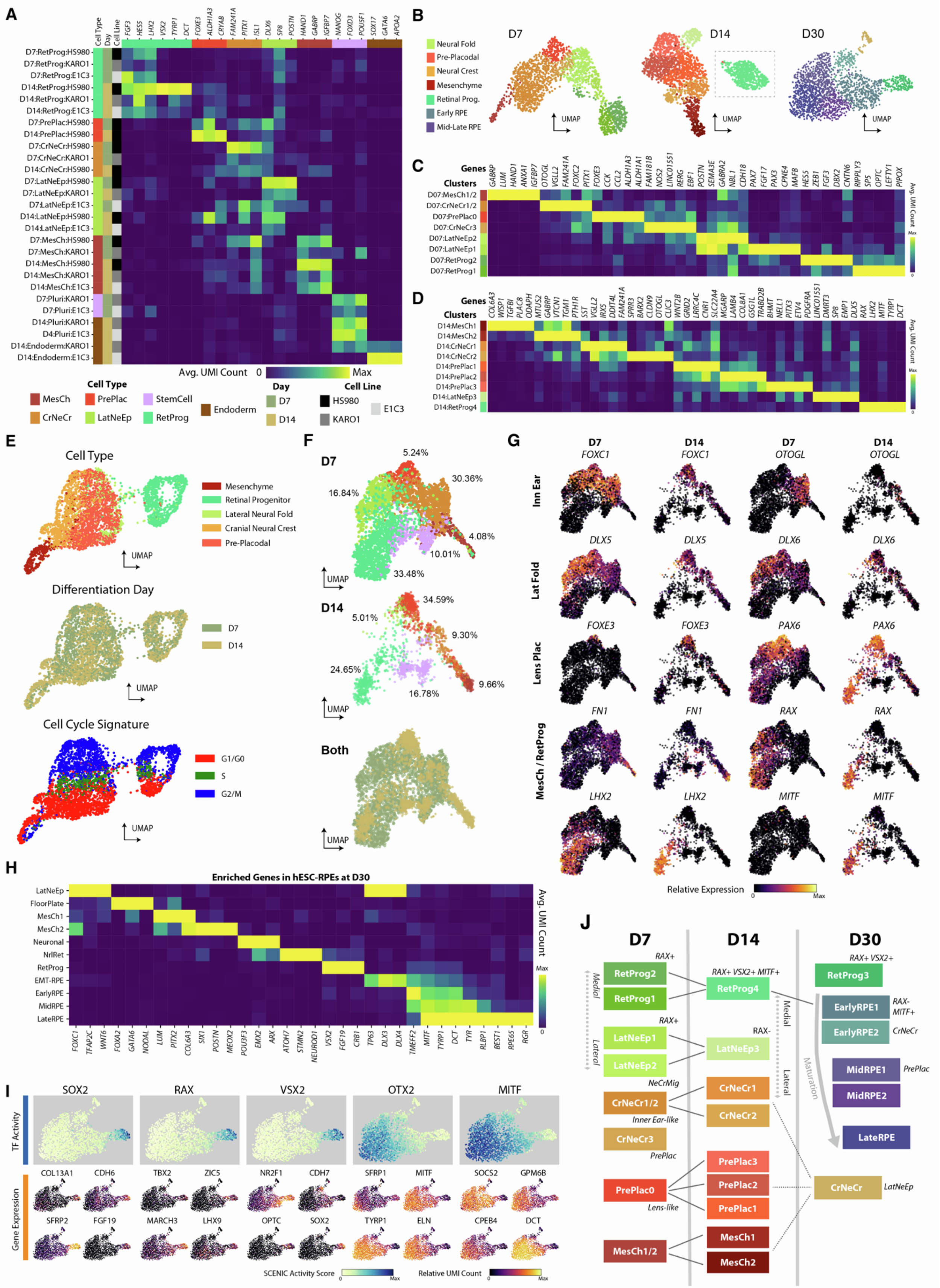
A
Cell Type
Day
Cell Line
D7:RetProg:HS980
D7:RetProg:KARO1
D7:RetProg:E1C3
D14:RetProg:HS980
D14:RetProg:KARO1
D14:RetProg:E1C3
D7:PrePlac:HS980
D14:PrePlac:HS980
D7:CrNeCr:HS980
D7:CrNeCr:KARO1
D14:CrNeCr:HS980
D7:LatNeEp:HS980
D7:LatNeEp:KARO1
D7:LatNeEp:E1C3
D14:LatNeEp:HS980
D14:LatNeEp:E1C3
D7:MesCh:HS980
D7:MesCh:KARO1
D14:MesCh:HS980
D14:MesCh:KARO1
D14:MesCh:E1C3
D7:Pluri:KARO1
D7:Pluri:E1C3
D14:Pluri:KARO1
D4:Pluri:E1C3
D14:Endoderm:KARO1
D14:Endoderm:E1C3
Avg. UMI Count 0 Max
MesCh
CrNeCr
PrePlac
LatNeEp
StemCell
RetProg
Endoderm
D7
D14
HS980
KARO1
E1C3
B
Neural Fold
Pre-Placodal
Neural Crest
Mesenchyme
Retinal Prog.
Early RPE
Mid-Late RPE
D7
D14
D30
UMAP
C
Genes
Clusters
D07:MesCh1/2
D07:CrNeCr1/2
D07:PrePlac0
D07:CrNeCr3
D07:LatNeEp2
D07:LatNeEp1
D07:RetProg2
D07:RetProg1
D
D14:MesCh1
D14:MesCh2
D14:CrNeCr1
D14:CrNeCr2
D14:PrePlac1
D14:PrePlac2
D14:PrePlac3
D14:LatNeEp3
D14:RetProg4
E
Cell Type
Mesenchyme
Retinal Progenitor
Lateral Neural Fold
Cranial Neural Crest
Pre-Placodal
Differentiation Day
Cell Cycle Signature
G1/G0
S
G2/M
F
D7
5.24%
30.36%
16.84%
4.08%
10.01%
33.48%
D14
34.59%
5.01%
9.30%
24.65%
9.66%
16.78%
Both
G
Inn Ear
Lat Fold
Lens Plac
MesCh / RetProg
Relative Expression
H
Enriched Genes in hESC-RPEs at D30
LatNeEp
FloorPlate
MesCh1
MesCh2
Neuronal
NrlRet
RetProg
EMT-RPE
EarlyRPE
MidRPE
LateRPE
I
SOX2
RAX
VSX2
OTX2
MITF
TF Activity
Gene Expression
SCENIC Activity Score
Relative UMI Count
J
D7
D14
D30
RetProg2
RetProg1
RetProg4
RetProg3
LatNeEp1
LatNeEp2
LatNeEp3
CrNeCr1/2
CrNeCr1
CrNeCr2
CrNeCr3
EarlyRPE1
EarlyRPE2
MidRPE1
MidRPE2
LateRPE
PrePlac0
PrePlac3
PrePlac2
PrePlac1
CrNeCr
MesCh1/2
MesCh1
MesCh2
Medial
Lateral
Maturation
Inner Ear-like
Lens-like
LatNeEp

Figure S2. Gene expression characterization and canonical correlation analysis of early differentiation. Related to Figure 2. (A) Heatmap of enriched genes by primary clusters, grouped by cell line, at D7 and D14 of hESC-RPE differentiation. **(B)** UMAP representation of HS980 differentiation at D7, D14, and D30, colored by cell type. **(C)** Heatmap of top enriched genes of each cell type cluster at D7. **(D)** Heatmap of top enriched genes of each cell type cluster at D14. **(E)** D7 and D14 cell HS980 populations projected on a shared low dimensional subspace using canonical correlation analysis (CCA; see Experimental Procedures), colored by cell type, differentiation day, and cell cycle phase. **(F)** UMAP representation of D7 and D14 cells, across all three lines, integrated with CCA. **(G)** UMAPs showing gene expression in all three lines of fundamental cell type markers for Inner Ear (InnEar), Lateral Fold (LatFold), Lens Placode (LensPlac), Mesenchyme (MesCh), and Retinal Progenitor (RetProg) from D7 to D14. Expression of neural crest inner ear (*FOXC1*, *OTOGL*) and lateral fold (*DLX5*, *DLX6*) markers decreases from D7 to D14. **(H)** Heatmap of enriched genes by cell type at D30 across all three lines. **(I)** Top: Transcription factor (TF) activity scores for SOX2, RAX, VSX2, OTX2, and MITF obtained by SCENIC analysis. Bottom: UMAPs showing gene expression of the top four inferred target genes of each TF at D30 of differentiation in HS980. **(J)** Schematic of the proposed relationship among the various secondary clusters during pigmentation induction. Edges indicate putative relationships between cell types identified at different time points.

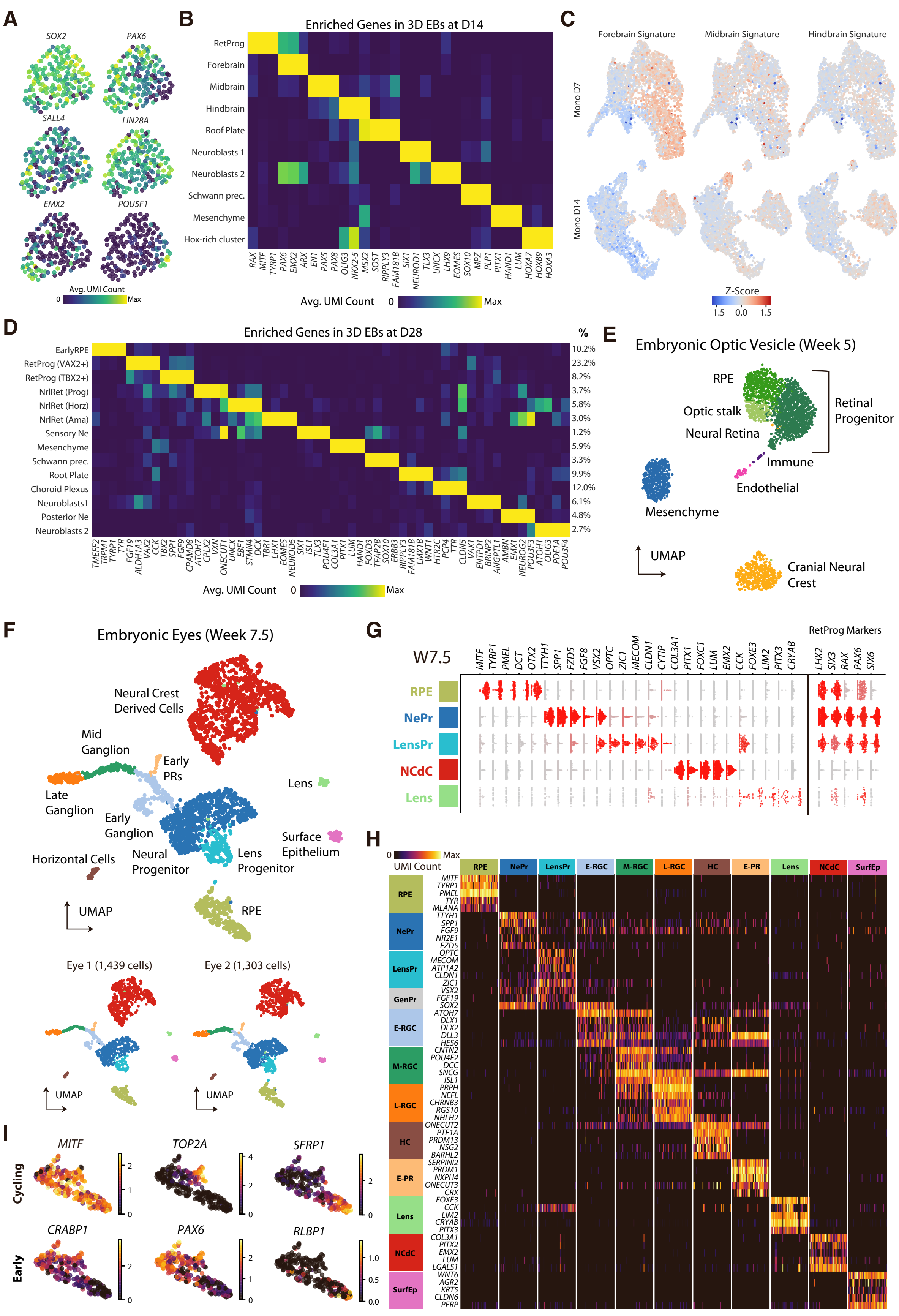
A
SOX2
PAX6
SALL4
LIN28A
EMX2
POU5F1
Avg. UMI Count
0
Max
B
Enriched Genes in 3D EBs at D14
RetProg
Forebrain
Midbrain
Hindbrain
Roof Plate
Neuroblasts 1
Neuroblasts 2
Schwann prec.
Mesenchyme
Hox-rich cluster
Avg. UMI Count
0
Max
C
Forebrain Signature
Midbrain Signature
Hindbrain Signature
Mono D7
Mono D14
Z-Score
−1.5
0.0
1.5
D
Enriched Genes in 3D EBs at D28
%
EarlyRPE
RetProg (VAX2+)
RetProg (TBX2+)
NrlRet (Prog)
NrlRet (Horz)
NrlRet (Ama)
Sensory Ne
Mesenchyme
Schwann prec.
Root Plate
Choroid Plexus
Neuroblasts1
Posterior Ne
Neuroblasts 2
10.2%
23.2%
8.2%
3.7%
5.8%
3.0%
1.2%
5.9%
3.3%
9.9%
12.0%
6.1%
4.8%
2.7%
Avg. UMI Count
0
Max
E
Embryonic Optic Vesicle (Week 5)
RPE
Optic stalk
Neural Retina
Retinal Progenitor
Immune
Endothelial
Mesenchyme
UMAP
Cranial Neural Crest
F
Embryonic Eyes (Week 7.5)
Neural Crest Derived Cells
Mid Ganglion
Early PRs
Late Ganglion
Early Ganglion
Lens
Surface Epithelium
Horizontal Cells
Neural Progenitor
Lens Progenitor
UMAP
RPE
Eye 1 (1,439 cells)
Eye 2 (1,303 cells)
UMAP
UMAP
G
W7.5
RetProg Markers
RPE
NePr
LensPr
NCdC
Lens
H
0
Max
UMI Count
RPE
NePr
LensPr
GenPr
E-RGC
M-RGC
L-RGC
HC
E-PR
Lens
NCdC
SurfEp
I
Cycling
Early
MITF
TOP2A
SFRP1
CRABP1
PAX6
RLBP1

Figure S3. Characterization of RPE differentiation in 3D embryoid bodies and compared to embryonic references. Related to Figure 3. (A) UMAP representation of EB differentiation a D7 (181 cells), colored by normalized UMI count of progenitor (*SOX2, PAX6*), pluripotency (*SALL4, LIN28A*) and regional (*EMX2*, *POU5F1*) marker genes. **(B)** Heatmap of enriched genes by cell type at EB at D14. **(C)** Signature scores for forebrain, midbrain and hindbrain visualized on D7 and D14 monolayer cells. Scores were computed as those in Figure 3B. **(D)** Heatmap of enriched genes by cell type at EB at D28, with percent composition of total EB population. **(E)** UMAP representation of a human embryonic optic vesicle (2,637 cells) dissected at 5 weeks (Carnegie Stage 13). Cluster identities include: optic cell types derived from retinal progenitors (RetProg), such as retinal pigment epithelium (RPE), neural retina (NR), and optic stalk (OS), in addition to periocular mesenchyme (MesCh), cranial neural crest (CrNeCr), immune, and smooth muscle. **(F)** Top: UMAP representation of scRNA-seq data from two human fetal eyes at week 7.5, colored by cell type. Bottom: UMAP of each individual fetal eye separately. **(G)** Violin plots of enriched genes in the identified W7.5 clusters. **(H)** Heatmap of normalized enriched gene expression. Genes were selected using an enrichment score by cell type in (F). **(I)** Retinal progenitor and RPE log2 normalized gene expression of cycling, Early and Mid RPE markers in the RPE cell cluster from (F).

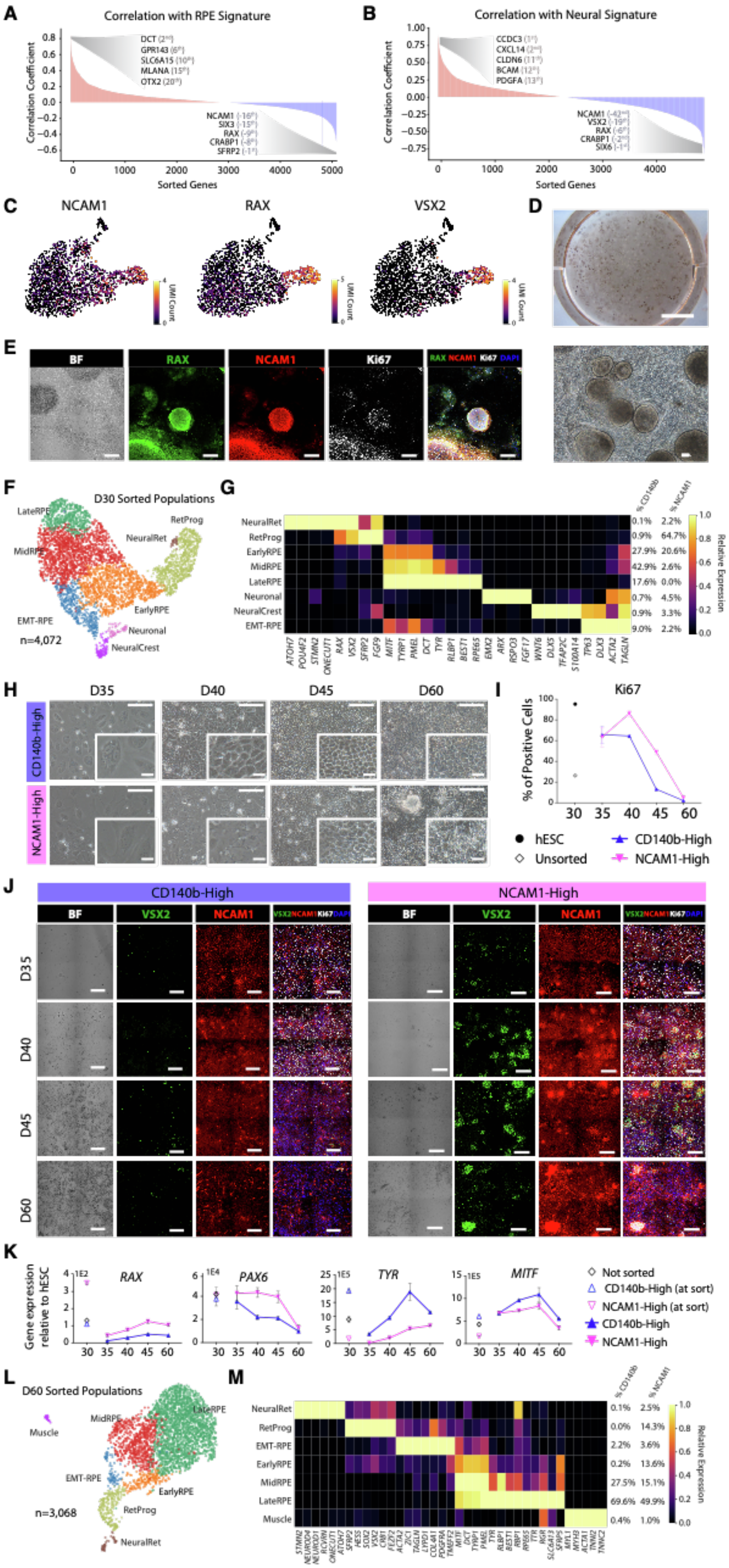
A
Correlation with RPE Signature
Correlation Coefficient
Sorted Genes
B
Correlation with Neural Signature
Correlation Coefficient
Sorted Genes
C
NCAM1
RAX
VSX2
UMI Count
D
E
BF
RAX
NCAM1
Ki67
F
D30 Sorted Populations
LateRPE
RetProg
NeuralRet
MidRPE
EarlyRPE
EMT-RPE
Neuronal
NeuralCrest
n=4,072
G
Relative Expression
H
D35
D40
D45
D60
CD140b-High
NCAM1-High
I
Ki67
% of Positive Cells
hESC
Unsorted
CD140b-High
NCAM1-High
J
CD140b-High
NCAM1-High
BF
VSX2
NCAM1
D35
D40
D45
D60
K
Gene expression relative to hESC
RAX
PAX6
TYR
MITF
Not sorted
CD140b-High (at sort)
NCAM1-High (at sort)
CD140b-High
NCAM1-High
L
D60 Sorted Populations
Muscle
MidRPE
LateRPE
EMT-RPE
EarlyRPE
RetProg
NeuralRet
n=3,068
M
Relative Expression

Figure S4. Characterization of CD140b-High and NCAM1-High sorted populations exposed to RPE differentiation conditions. Related to Figure 4. (A-B) Pearson's correlation coefficients were computed between 5,412 and an RPE signature (A) or 4,664 genes and a neural signature (B) using normalized counts and cells belonging to the D30 retinal progenitor and RPE clusters or retinal progenitor and neural clusters, respectively. **(C)** UMAPs showing gene expression of progenitor markers in scRNA-seq hESC-RPE at D30 in the HS980 cell line. **(D)** Camera pictures of hESC-RPE D30 cultures. **(E)** Brightfield and immunofluorescence stainings of hESC-RPE D30 cells showing co-expression of RAX, NCAM1 and Ki67 markers. Scale bars: top 1mm; bottom 100µm. **(F)** scRNA-seq of 4,072 single cells from NCAM1-High sorted, CD140b-High sorted, and unsorted D30 cells, colored by cell type. See Figure 4E for composition by sample identifier. **(G)** Heatmap of enriched marker genes in cell types of sorted populations at D30, with cell type composition percentages for both sorted populations. **(H)** Brightfield images of unsorted, CD140b-High and NCAM1-High populations at D33, D40, D45, and D60. FACS sorting of the two populations was performed at D30. Scale bars: 100µm; inset 20µm. **(I)** Graph showing the percentage of positive cells expressing the Ki67 proliferation marker in hESC, unsorted, CD140b-High and NCAM1-High populations at the moment of FACS sorting (D30) and D35, D40, D45, and D60. Bars represent mean +/-SEM from three independent experiments. **(J)** Brightfield and immunofluorescence images showing expression of VSX2 and NCAM1 in unsorted, CD140b-High and NCAM1-High populations after FACS sorting at differentiation D35, D40, D45, and D60. Scale bars: 200µm. **(K)** Graphs representing RT-qPCR of retinal progenitor (*RAX*, *PAX6*) and RPE (*MITF*, *TYR*) marker genes in unsorted, CD140b-High and NCAM1-High populations at the moment of sort and at post-sort D30, 35, 40, 45, and 60. **(L)** scRNA-seq of 3,068 single cells from NCAM1-High sorted, CD140b-High sorted, and unsorted D60 cells, colored by cell type. See Figure 4K for composition by sample identifier. **(M)** Heatmap of enriched marker genes in cell types of sorted populations at D60, with cell type composition percentages for both sorted populations.

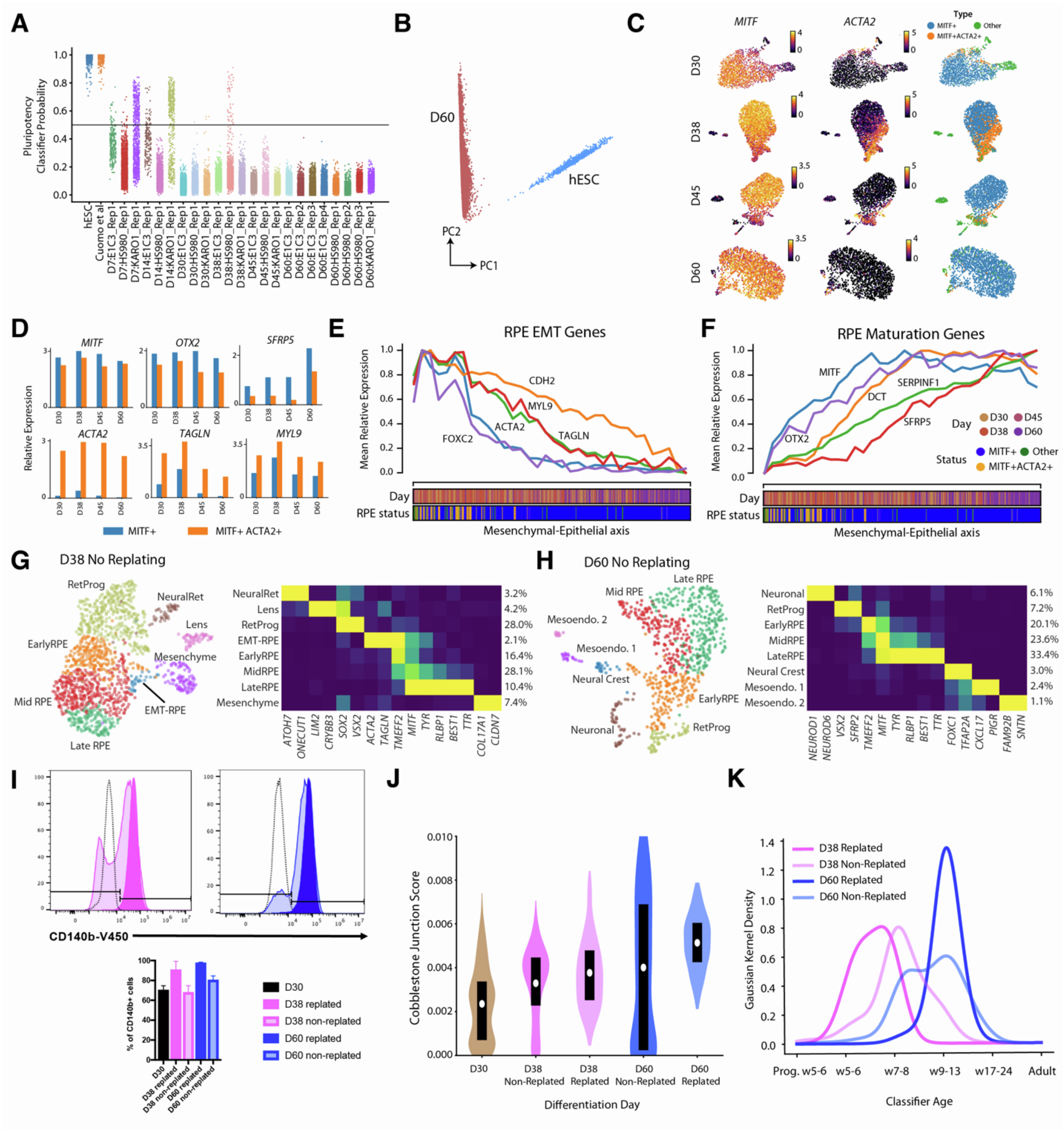

Figure S5. Characterization of late differentiation and overall gene expression correlation. Related to Figure 6. (**A**) Plot displaying the probability outputted by a pluripotency classifier when inputted data from *in vitro* cells at all time points. A random forest classifier was trained a mixture of hESCs from this and another study (Cuomo et al 2020; see Experimental Procedures) (**B**) Scatter plot of the two first principal components of all D60 and hESC *in vitro* cells. (**C**) UMAP overlayed with *MITF* and *ACTA2* gene

expression at D30, D38, D45, and D60. Cells are labeled as *MITF*+ (maturing RPE), *MITF*+*ACTA2*+ (EMT-RPE), or other (non-RPE cell types). (**D**) Bar graphs showing the gene expression differences between EMT-RPE and maturing RPE. EMT-RPE expresses EMT markers *ACTA2, TAGLN,* and *MYL9* more highly, whereas RPE markers *MITF, OTX2,* and *SFRP5* are more highly expressed in non-transitioning RPE. (**E-F**) Line plots showing average expression of RPE-EMT (E) and mature RPE (F) along a mesenchymal-epithelial axis of variation determined by fitting a principal curve (see Experimental Procedures). Colored bars on the x-axis indicate time point and RPE status of cells along the axis. (**G**) Left: UMAP representation of 1,423 single cells at hESC-RPE D38 without replating at D30. Right: heatmap of enriched genes by cell type. (**H**) Left: UMAP representation of 772 single cells at hESC-RPE D60 without replating at D30. Right: heatmap of enriched genes by cell type. (**I**) Top: representative flow cytometry plots for HS980 cell line showing CD140b cell surface marker expression at D38 and D60 for replated and non-replated conditions. Dotted lines represent hESC (negative control). Bottom: bar graphs show the average of CD140b marker expression in the stated conditions and time points for both HS980 and KARO1 cell lines. (**J**) Violin plot displaying the quantification of cobblestone morphology at D38 and D60 with and without replating in the HS980 cell lines using the junction score methodology and software developed by Joshi et al, J Ocul Pharmacol Ther. 2016. (**K**) Graph showing distribution of classifications of D38 and D60 RPE cultures, with and without replating (see also Figures 6 and S6). Bars represent mean +/-SEM from three independent experiments.

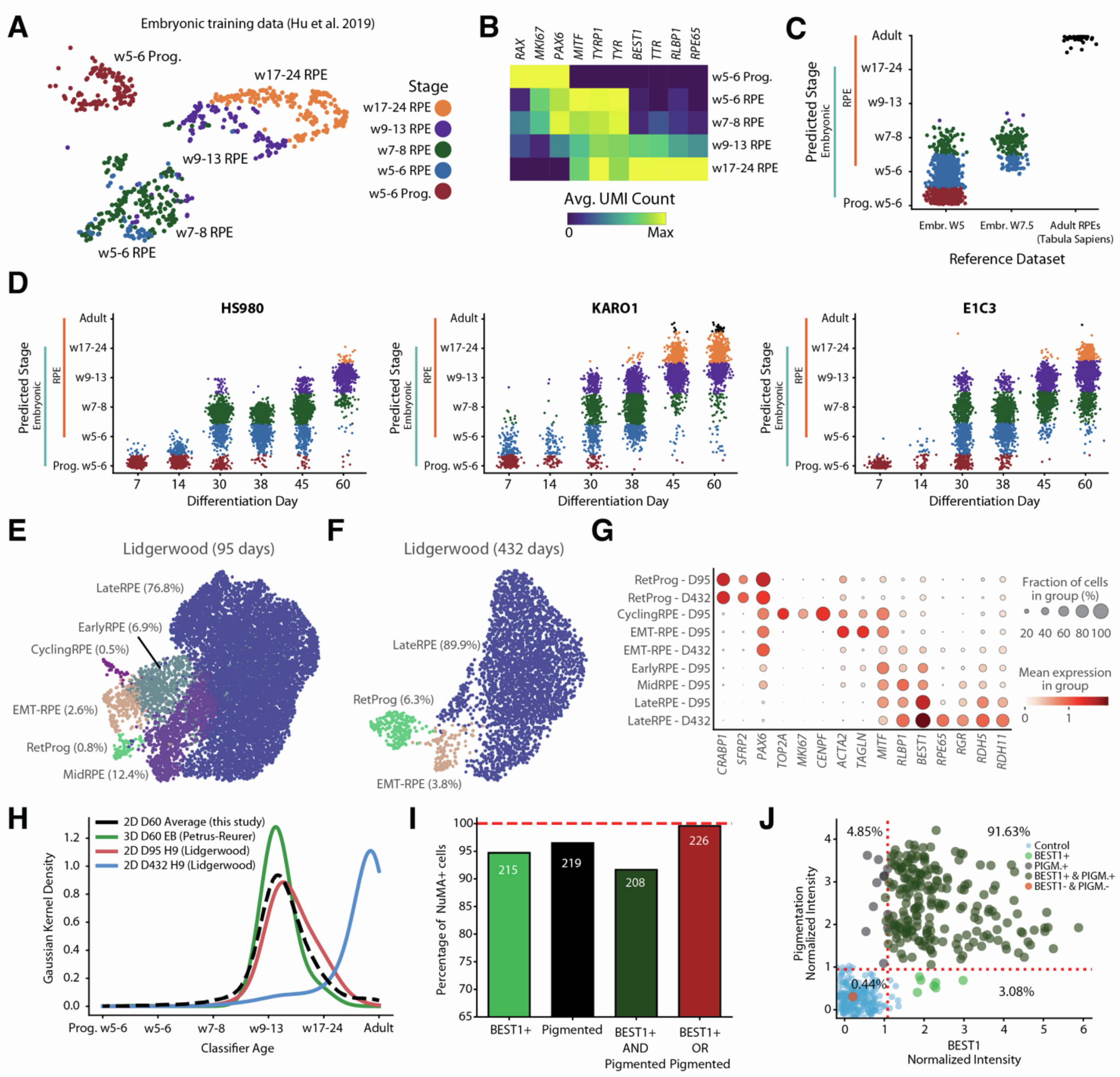

Figure S6. Ordinal classification of *in vitro* hESC-RPE. Related to Figure 6. (A) UMAP representation of Hu et al dataset of 783 human fetal cells from various time points in development, as used in the ordinal classifier (see Figure 7). Cells were colored into five categories for classification. **(B)** Heatmap showing an overview of uniquely enriched retinal progenitor and RPE marker genes in the data in (D). **(C)** Plot showing ordinal classification of reference embryonic RPEs at weeks 5 and 7.5 (see Figures 3 and S3). **(D)** Plots showing ordinal classification for hESC-RPE differentiation data in the HS980, KARO1, and E1C3 cell lines individually. **(E)** UMAP representation of RPE differentiation

day 90 in H9 cell line (9,456 single cells) re-analyzed from Lidgerwood et al, Genomics Proteomics Bioinformatics 2020. Cells colored and labeled by newly-annotated cell types. **(F)** UMAP representation of RPE differentiation day 432 (1 Year) in H9 cell line (3,216 single cells) re-analyzed from Lidgerwood et al, Genomics Proteomics Bioinformatics 2020. Cells colored and labeled by newly-annotated cell types. **(G)** Dot plot of marker gene expression for RetProg, CyclingRPE, EMT-RPE, EarlyRPE, MidRPE, and LateRPE in (A-B). **(H)** Graph showing distribution of ordinal classifications of various differentiated RPE culture protocols, including the 2D monolayer protocol from this study (20,682 cells), 3D EBs at D60 (Petrus-Reurer et al, 2020; 294 cells), 2D D95 H9 (Lidgerwood et al., 2021; 9,456 cells) and 2D D432 H9 (Lidgerwood et al., 2021; 3,216 cells). **(I)** Bar graph of 227 hESC-RPE grafted cell BEST1 and pigmentation statuses after 30 days into the albino rabbit subretinal space. NuMA+ human cells from ten sections and three rabbits were manually segmented and assessed by immunofluorescence (BEST1 expression) and brightfield (Pigmentation). **(J)** Scatter plot of the BEST1 normalized intensity and the pigmentation normalized intensity for 227 grafted NuMA+ cells and 227 NuMA- control cells. Cells are colored by histological status (Control, BEST1+, PIGM+, BEST1 & PIGM+, and BEST1- & PIGM-). Red dotted lines indicate the 97.5th percentile threshold of the signal observed in the negative control cells.

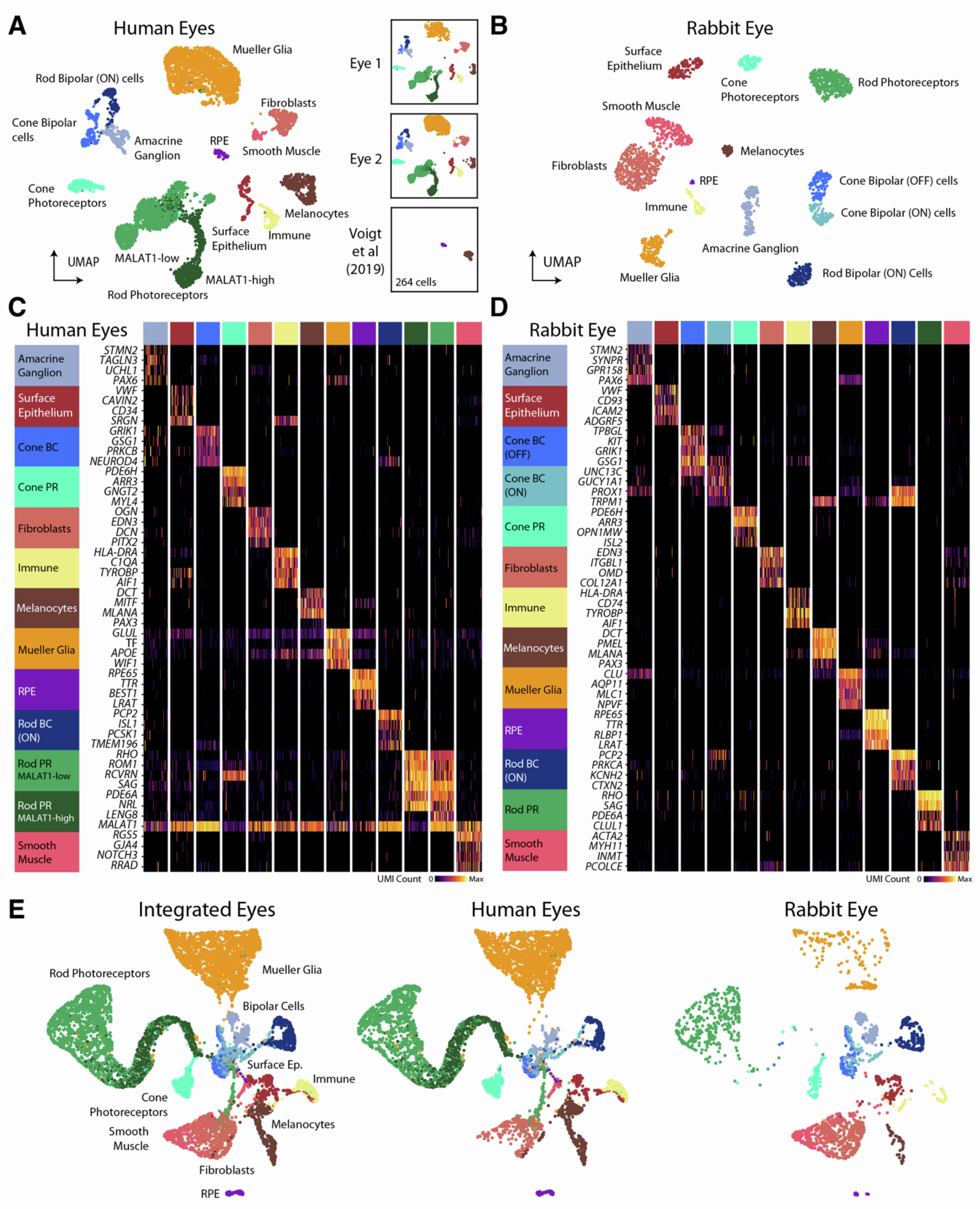

Figure S7. Transcriptional analysis of albino rabbit and human retinas. Related to Figure 7. (A) Annotated UMAP representation of 5,538 human cells from two human

eyes (1,564 cells and 3,706 cells), categorized into 13 different cell types. Additional RPEs and melanocytes (264 cells) were re-analyzed and incorporated from Voigt et al, 2019. **(B)** Annotated UMAP representation of 1,965 rabbit cells categorized into 13 different retina cell types. **(C)** Heatmap of enriched marker genes for adult eye cell types. **(D)** Heatmap of enriched marker genes for rabbit eye cell types. Genes were selected from among the top 20 enriched genes per cluster for (C) and (D). **(E)** CCA integration of human and rabbit eyes. Integration was performed using Seurat on 2,000 enriched genes from a total of 9,889 genes with shared annotations between the two species (see Experimental Procedures).

SUPPLEMENTAL TABLES

Table S1. Overview of all scRNA-seq samples generated and quality control conditions. Related to Experimental Procedures.

See attached TableS1.xlsx file.

Table S2. Gene enrichment during hESC-RPE pigmentation induction (D7, D14, and D30) by cell type and time point. Related to Figure 2.

See attached TableS2.xlsx file.

Table S3. Gene enrichment for embryonic eyes at Carnegie Stages 12, 13, 14, 15, and 20 by cell type and time point. Related to Figure 3.

See attached TableS3.xlsx file.

Table S4. Gene correlation scores with retinal progenitor and neural tube signature during the identification of NCAM1 cell surface marker. Related to Figure 4.

See attached TableS4.xlsx file.

Table S5. Gene enrichment during late hESC-RPE differentiation (D38, D45, D60) by cell type. Related to Figure 6.

See attached TableS5.xlsx file.

Table S6. Gene enrichment scores for adult human and rabbit eyes by cell type. Related to Figure S7.

See attached TableS6.xlsx file.

EXPERIMENTAL PROCEDURES, Related to Experimental Procedures.

RESOURCE AVAILABILITY

Lead Contact

Further information and requests for resources and reagents should be directed to and will be fulfilled by the Lead Contact, Fredrik Lanner (fredrik.lanner@ki.se).

MATERIALS AVAILABILITY

This study did not generate new unique reagents.

DATA AND CODE AVAILABILITY

All scRNA-seq datasets generated for this study are available as raw feature-count matrices, as generated by CellRanger, at the accession number GSE164092. Raw FASTQ files for all samples are also available, with exception of those from E1C3 cell line, for which specific approval will be needed from Novo Nordisk. Processed loom/h5ad files containing cell type annotations, UMAP embedding coordinates, and other metadata as well as the custom reference genome used for human and rabbit pooled analysis can be downloaded at the GEO accession above. Enriched gene lists, correlation scores, and other metadata are provided as Supplemental Tables. Jupyter notebooks (in both ipynb and html formats) to reproduce the single cell analyses performed in this study, as well as all custom companion source code, are shared at https://github.com/lamanno-epfl/rpe_differentiation_profiling_code. Datasets are available for interactive visualization and analysis using the ASAP web resource (https://asap.epfl.ch/) under public keys ASAP 75-90 (David et al., 2020; Gardeux et al., 2017). Human embryonic reference datasets of optic vesicle/cup from Carnegie Stages 12, 13, 14, and 15 can be downloaded at https://data.nemoarchive.org/biccn/lab/kriegstein/transcriptome/. The external scRNA-seq reference datasets used in this study can be found at the original accession numbers: GSE135922 (Voigt et al., 2019) GSE107618 (Hu et al., 2019), GSE116106 (Lu et al., 2020) or on ArrayExpress at E-MTAB-8511 (Lidgerwood et al., 2021).

hESC CELL CULTURE

HS980 and KARO1 were previously derived and cultured under xeno-free and defined conditions (Rodin et al., 2014) following informed consent by donors (Swedish Ethical Review Authority: 2011/745:31/3). E1C3 (NN GMP0050E1C3) was provided as a research cell bank of the clinical GMP cell line by NovoNordisk (UCSF IRB: 1518222, for RPE differentiation Projekt-ID: H-18016740, Anmeldelsesnr.: 73105). All hESCs maintain a normal karyotype and trilineage differentiation potential, and are routinely tested for mycoplasma. Cells were maintained on hrLN-521 (10µg/mL, Biolamina) or iMatrix-511 (0.25 µg/cm^2, Nippi, T303) in NutriStem hPSC XF medium (Biological Industries) and a 5% CO_2/5% O_2 incubator.

Cells were passaged enzymatically at a 1:10 ratio every 5-6 days. Confluent cultures were washed twice with PBS without Ca^{2+} and Mg^{2+} and incubated for 5 min at 37°C, 5% CO_2/5% O_2 with TrypLE Select (ThermoFisher Scientific, 12563011). The enzyme was then carefully removed and cells were collected in fresh pre-warmed NutriStem hPSC XF medium by gentle pipetting to obtain a single cell suspension. Cells were centrifuged at 300 g for 4 min, the pellet resuspended in fresh prewarmed NutriStem hPSC XF medium, and plated on a freshly hrLN-521 coated dish. Two days after passage, the medium was replaced with fresh pre-warmed NutriStem hPSC XF medium and changed daily.

2D and 3D hESC-RPE *in vitro* DIFFERENTIATION

A step-by-step protocol describing the differentiation procedure has been reported previously (Plaza Reyes et al., 2020a, 2020b). hESCs were plated at a cell density of $2.4x10^4$ cells/cm^2 on hrLN-521 (20 µg/mL) using NutriStem hPSC XF medium. A Rho-kinase inhibitor (Millipore, Y-27632) at a concentration of 10 µM was added during the first 24h, while cells were kept at 37°C, 5% CO_2/5% O_2. After 24h, hPSC medium was replaced with differentiation medium NutriStem hPSC XF without bFGF and TGFβ and cells were placed at 37°C, 5% CO_2/21%O_2. From day 6 after plating, 100 ng/mL of Activin A (R&D Systems, 338-AC-050) was added to the media. Cells were fed three times a week and kept for 30 days. Cells that did not undergo replating after 30 days were kept in these conditions for 60 days, but without the addition of Activin A from day 30 until day

of analysis. During standard differentiations, with replating, monolayers were then trypsinized using TrypLE Select (ThermoFisher Scientific, 12563011) for 10 min at 37°C, 5% CO_2. The enzyme was carefully removed and the cells were collected in fresh pre-warmed NutriStem hPSC XF medium without bFGF and TGFβ by gentle pipetting to obtain a single cell suspension. Cells were centrifuged at 300 g for 4 min, the pellet was resuspended, passed through a cell strainer (ø 40 µm, VWR, 732-2757) and seeded on laminin coated dishes (hrLN-521 at 20µg/mL) at $6.8x10^4$ cells/cm^2. Replated cells were fed three times a week during the subsequent 30 days with NutriStem hPSC XF medium without bFGF and TGFβ. Brightfield images were acquired with a Nikon Eclipse TE2000-S microscope and a Canon SX170 IS camera was used to capture pigmentation from the top of the wells.

3D hESC-RPE Embryoid Body (EB) differentiations were cultured following the differentiation procedure reported previously (Plaza Reyes et al., 2016). Pluripotent stem cells (HS980 line) were cultured to confluence on hrLN-521-coated plates and manually scraped to produce EBs using a 1000 µl pipette tip. The EBs were then cultured in suspension in low attachment plates at a density of $5\text{-}7x10^4$ cells/cm^2. Differentiation was performed in NutriStem hPSC XF medium without bFGF and TGFβ with media changed twice a week for 28-30 days. 10 µM Rho-kinase inhibitor (Millipore Y-27632) was added to the suspension cultures only during the first 24h.

EVALUATION OF COBBLESTONE MORPHOLOGY

Brightfield images acquired with a 10x objective were analyzed to quantify the cobblestone morphology of RPE monolayers generated in culture. To this aim, we made use of the Steerable Wavelet Filters algorithm (Joshi et al., 2016). In particular, we used two MATLAB packages: “rpe”, a dedicated package to estimate morphology (https://git-bioimage.coe.drexel.edu/opensource/rpe) and “circular-wavelets”, its dependency, and the Steerable Wavelet Filters implementation from the package. We processed the raw images by applying the function “convolve_image_open” and extracted the unnormalized SWF filter response. We divided the images into small tiles of 128x128 pixels and, for each of the tiles, computed the cobblestone junction score of each tile as the total SWF

response per µm². Distributions were then displayed using violin plots to highlight eventual variability of the score in different regions of the image.

FLOW CYTOMETRY and CELL SORTING

hPSC-RPE growing on the tested substrates were dissociated into single cells using TrypLE Select (ThermoFisher Scientific, 12563011). Samples were stained with BV421 Mouse Anti-Human CD140b (BD Biosciences 564124, clone [28D4], 10 µg/mL), BB515 Mouse Anti-Human CD56 (BD Biosciences 564489, clone [B159], 2.5 µg/mL), PE Mouse anti-human Ki-67 (Biolegend 350504, clone [Ki-67], 50 µg/mL) conjugated antibodies, diluted in 2% FBS and 1 mM EDTA (ThermoFisher Scientific, 10082147). Cells were incubated with the conjugated antibodies on ice for 30 min. Fluorescence minus one (FMO) controls were included for each condition to identify and gate negative and positive cells. Stained cells were analyzed using a CytoFLEX flow cytometer equipped with 488 nm, 561 nm, 405 nm and 640 nm lasers (Beckman Coulter). Analysis of the data was carried out using FlowJo v.10 software (Tree Star).

Cell sorting was performed on hPSC-RPE cultures after 30 days of differentiation. Cells were incubated with the mentioned conjugated antibodies on ice for 30 min. Fluorescence minus one (FMO) controls were included for each condition to identify and gate negative and positive cells. Stained cells were then sorted using a BD FACS Aria Fusion Cell Sorter (BD Bioscience) using FACSDiva Software v8.0.1. After sorting, specific cell populations (NCAM1-High, CD140b-High and unsorted counterparts) were cultured under RPE conditions (see replated cells in 2D and 3D hESC-RPE *In Vitro* Differentiation) or under Neuroretinal progenitor conditions (see Neuroretinal Progenitor *In Vitro* Differentiation).

NEURORETINAL PROGENITOR *in vitro* DIFFERENTIATION

Directly after sorting, 68,420 cells/cm^2 were plated on matrigel coated plates (HS980 line). Cells were cultured until confluency in P1 media with DMEM/F12 (ThermoFisher Scientific, 11320033) as basal media containing B27 (ThermoFisher Scientific, 17504044), N2 (ThermoFisher Scientific, 17502048), 10ng/mL hDKK1 (R&D Systems, 5439-DK-010), 10ng/mL mouse Noggin (R&D Systems, 1967-NG-025), 10ng/mL hIGF-1

(R&D Systems, 291-G1-200), 5ng/mL bFGF (ThermoFisher Scientific, 13256029) and 50 U/mL of Penicillin-Streptomycin (ThermoFisher Scientific, 15140122). Thereafter and until day 40, cells were continued cultured in P1 with the addition of 40ng/mL 3,3′,5-Triiodo-L-thyronine T3 (Sigma, T-074-1ML) and 100uM Taurine (Sigma, T8691-25G). This protocol was based on previous work (Shao et al., 2017).

qPCR

Total RNA was isolated using the RNeasy Plus Mini Kit and treated with RNase-free DNase (both from Qiagen, 74106 and 79254, respectively). cDNA was synthesized using 1 µg of total RNA in a 20 µL reaction mixture, containing random hexamers and Superscript III reverse transcriptase (ThermoFisher Scientific, 18080085), according to the manufacturer's instructions. Taq-polymerase together with Taqman probes (ThermoFisher Scientific) for *MITF* (Hs01117294_m1), *BEST-1* (Hs00188249_m1), *RPE65* (Hs01071462_m1), *TYR* (Hs00165976_m1), *SIX6* (HS00201310_m1), *PAX6* (HS01088114_m1), *VSX2* (HS01584046_m1), *RAX* (HS00429459_m1), and *GAPDH* (4333764F) were used. Samples were subjected to the real-time PCR amplification protocol on StepOne™ real-time PCR System (Applied Biosystems). Three independent experiments were performed for every condition and technical duplicates were carried for each reaction. Results are presented as mean ± SEM (standard error of the mean).

ENZYME-LINKED IMMUNOSORBENT ASSAY (ELISA)

Day 30 unsorted, CD140b-High and NCAM1-High sorted hESC-RPE cells were cultured for extra 30 days on Transwell membranes (0.33 cm^2, Merck Millipore, PTHT24H48) coated with hrLN-521 (20 µg/mL). Supernatants from both the apical and basal sides (meaning upper and lower compartments of the transwell, respectively) were collected 60h after the last medium change. PEDF secretion levels were measured in triplicates for each condition with commercially available human PEDF ELISA Kit (BioVendor, RD191114200R), in accordance with manufacturer's instructions. The optical density readings were measured using SpectraMax 250 Microplate Reader (Molecular Devices). Results are presented as mean ± SEM.

TRANSEPITHELIAL ELECTRICAL RESISTANCE (TEER)

Day 30 unsorted, CD140b-High and NCAM1-High sorted hESC-RPE cells were cultured for extra 30 days on Transwell membranes (0.33 cm^2, Merck Millipore, PTHT24H48) coated with hrLN-521 (20 μg/mL). TEER readings were measured using the Millicell Electrical Resistance System volt-ohm meter (Millicell ERS-2 Voltohmmeter, MERS00002, Millipore), in accordance with manufacturer's instructions. Cultures were equilibrated outside the incubator at room temperature for 15–20 min before the start of the readings. Measurements were performed in unchanged culture media in triplicates for each condition and well, at three different well positions. Respective averages were used for further analysis. The background resistance was determined from a blank culture insert in the same media coated with substrate but without cells (also in triplicates). Average blank was subtracted from the averaged triplicates per well. Measurements are reported as resistance in ohms times the surface area in square centimeters ($\Omega \times cm^2$). Results are presented as mean ± SEM.

IMMUNOCYTOFLUORESCENCE

Protein expression of day 60 hPSC-RPE monolayers was assessed with immunofluorescence. Cells were fixed with 4% methanol-free formaldehyde formaldehyde (VWR, FFCHFF22023000) at room temperature for 10 min, followed by permeabilization with 0.3% Triton X-100 (Sigma, T9284) in Dulbecco's phosphate-buffered saline (D-PBS, ThermoFisher Scientific, 14190094) for 10 min and blocking with 4% fetal bovine serum (FBS, ThermoFisher Scientific, 10082147) and 0.1% Tween-20 (Sigma, P9416) in DPBS for 1 hour. Primary antibodies were diluted to the specified concentrations in 4% FBS, 0.1% Tween-20, DPBS solution: VSX2/Chx10 (1:50, Santa Cruz Biotechnology sc-365519, clone [E-12]), Ki67 (1:400, Cell Signaling Technology 9027, clone [D2H10]), Bestrophin 1 (BEST-1) (1:100, Millipore MAB5466), Zonula occludens-1 (ZO-1) (1:100, Invitrogen, 40-2200), RAX (10 ug/mL, Novusbio H00030062-M02, clone [4F4]), PDGFRB (CD140b) (1:100, BD Biosciences, 558820, clone [28D4]) and NCAM1 (CD56) (1:100, BD Biosciences, 555513, clone [B159]). The primary antibodies were incubated overnight at 4°C followed by 2 hours incubation at room temperature with secondary antibodies: donkey anti-mouse IgG (H+L) Alexa Fluor 488,

donkey anti-mouse IgG Alexa Fluor (H+L) 555, donkey anti-mouse IgG Alexa Fluor (H+L) 647, donkey anti-rabbit IgG (H+L) Alexa Fluor 647 (all of them from ThermoFisher Scientific, A21202, A31570, A31571, A31573, respectively) diluted 1:1,000 in 4% FBS, 0.1% Tween-20, D-PBS solution. Nuclei were stained with Hoechst 33342 (1:1,000, Invitrogen H3570). Images were acquired with Zeiss LSM710-NLO point scanning confocal microscope. Post-acquisition analysis of the pictures was performed using ImageJ v2.0 software.

HISTOLOGY and TISSUE IMMUNOSTAINING

Immediately after euthanasia by intravenous injection of 100 mg/kg pentobarbital (Allfatal vet. 100 mg/mL, Omnidea), the eyes were enucleated and the bleb injection area marked with green Tissue Marking Dye (TMD; Histolab Products AB, 02199). An intravitreal injection of 100 µL fixing solution (FS) consisting of 4% buffered formaldehyde (Histolab Products AB, 02175) was performed before fixation in FS for 24-48 hours and embedding in paraffin. Four-µm serial sections were produced through the TMD-labeled area. For immunostaining, slides were deparaffinized in xylene, dehydrated in graded alcohols, and rinsed with ddH_2O and Tris Buffered Saline (TBS, Sigma, 93352, pH 7.6). Antigen retrieval was achieved in 10 mM citrate buffer (trisodium citrate dihydrate, Sigma, S1804, pH 6.0) with 1:2000 Tween-20 (Sigma, P9416) at 96°C for 30 min, followed by 30 min cooling at room temperature. Slides were washed with TBS and blocked for 30 min with 10% Normal Donkey Serum (Abcam, ab138579) diluted in TBS containing 5% (w/v) IgG and protease-free bovine serum albumin (Jackson Immunoresearch, 001-000-162) in a humidified chamber. Primary antibodies diluted in the blocking buffer were incubated overnight at 4°C: human nuclear mitotic apparatus protein (NuMA) (1:200, Abcam ab84680), BEST-1 (1:200, Millipore MAB5466). Secondary antibodies (donkey anti-rabbit IgG (H+L) Alexa Fluor 555 A31572 and donkey anti-mouse IgG (H+L) Alexa Fluor 647 A31571, both from ThermoFisher Scientific) diluted 1:200 in blocking buffer, were incubated 1 hour at room temperature. Sections were mounted with vector vectashield with DAPI mounting medium (Vector Laboratories, H-1200-10) under a 24x50 mm coverslip. Images were taken with an Olympus IX81 fluorescence inverted microscope or

Zeiss LSM710-NLO point scanning confocal microscope. Post-acquisition analysis was performed using ImageJ v2.0 software.

SUBRETINAL INJECTIONS

hESC-RPE monolayers (HS980 line) were washed with DPBS (ThermoFisher Scientific, 14190-094), incubated with TrypLE (ThermoFisher Scientific, 12563-011) and dissociated to single cell suspension as described above. Cells were counted in a Neubauer hemocytometer (VWR, 631-0925) chamber using 0.4% trypan blue (ThermoFisher Scientific, 15250061), centrifuged at 300g for 4 min, and the cell pellet was resuspended in freshly filter-sterilized DPBS (ThermoFisher Scientific, 14190-094) to a final concentration of 1000 cells/µL. The cell suspension was then aseptically aliquoted into 600 µL units and kept on ice until surgery.

After approval by the Northern Stockholm Animal Experimental Ethics Committee (DNR N56/15), two female New Zealand white albino rabbits (provided by the Lidköpings rabbit farm, Lidköping, Sweden) aged 5 months and weighing 3.5 to 4.0 kg were used in this study. All experiments were conducted in accordance with the Statement for the Use of Animals in Ophthalmic and Vision Research.

As previously described (Bartuma et al., 2015; Petrus-Reurer et al., 2017, 2018; Plaza Reyes et al., 2016), animals were put under general anesthesia by intramuscular administration of 35 mg/kg ketamine (Ketaminol 100 mg/mL, Intervet, 511519) and 5 mg/kg xylazine (Rompun vet. 20 mg/mL, Bayer Animal Health, 22545), and the pupils were dilated with a mix of 0.75% cyclopentolate / 2.5% phenylephrine (APL, 321968). Microsurgeries were performed on both eyes using a 2-port 25G transvitreal pars plana technique (Alcon Nordic A/S, 8065751448). 25G trocars were inserted 1 mm from the limbus and an infusion cannula was connected to the lower temporal trocar. The cell suspension was drawn into a 1 mL syringe connected to an extension tube and a 38G polytip cannula (MedOne Surgical Inc, 3219 and 3223). Without infusion or prior vitrectomy the cannula was inserted through the upper temporal trocar. After proper tip positioning, ascertained by a focal whitening of the retina, 50 µL of cell suspension (equivalent to 50,000 cells) was injected slowly subretinally, approximately 6 mm below the inferior margin of the optic nerve head, forming a uniform bleb that was clearly visible

under the operating microscope. To minimize reflux, the tip was maintained within the bleb during the injection. After instrument removal light pressure was applied to the self-sealing suture-less sclerotomies. 2 mg (100 µL) of intravitreal triamcinolone (Triescence 40 mg/mL, Alcon Nordic A/S, 412915) was administered a day prior to the surgery, and no post-surgical antibiotics were given.

GRAFTED HESC-RPE QUANTIFICATION ANALYSIS

Human cells grafted in the RPE layer of the rabbit were recognized by the presence of nuclear NuMA staining and were segmented manually using the label paintbrush tool in *napari* (Sofroniew et al., 2021). The segmentations were used to quantify the total signal for BEST1 and the total level of pigmentation (using the brightfield channel) for each cell. Ten sections were analyzed from three different rabbits, and a total of 227 cells were segmented. An equal number of internal negative control cells were extracted from each image by segmenting cells of the sclera or neural retina. Each image was analyzed independently and then results pooled; the signal for the control cells was used to determine a minimal threshold to consider the cell positive. Specifically, for each image, we used the 97.5th percentile of the signal observed in negative control cells as a threshold for both BEST1 and pigmentation. For combined plotting, the intensity values were renormalized by first computing the square root of the signal and then dividing by the threshold intensity (so that the threshold sits at 1).

RETINAL TISSUE DISSOCIATION of HUMAN ADULT, EMBRYONIC and TRANSPLANTED RABBIT EYES for SINGLE-CELL RNA SEQUENCING

Human post-mortem research-consented donor eyes were obtained from the cornea bank at St. Erik Eye Hospital, Stockholm, Sweden. The use of human tissue was in accordance with the tenets of the Declaration of Helsinki and was approved by the Swedish legislative and ethical committee (#2019-02032) for the use of human donor material for research. Donors did not present any clinical diagnosis of ocular disease, and samples were anonymized and processed under the general data protection regulation. Two human eyes from the same donor were used (45-year-old male, 32 hours post-mortem). The lens was dissected out and the rest of the retina, except the sclera, was

chopped in several small pieces mixed together in 500 µL of digestion buffer (described below). Two embryonic eyes from the same embryo were used from a 7.5 post-conception week embryo. The optic cups were dissected out and chopped in several small pieces to facilitate dissociation in 500 µL of digestion buffer (described below). Donors (deceased, family for adult human eyes, or couples for the embryos) gave their informed consent for the donation and subsequent use for research purposes. The embryonic eyes were acquired from a clinical routine abortion after informed consent by the pregnant woman, in accordance with permissions from the regional ethical review board and the Swedish National Board of Health and Welfare ("Socialstyrelsen" #8.1-11692/2019) and the Swedish Ethical Review Authority ("EPN" #2007/1477-31/3. Two rabbit eyes (from different animals) with 30-day integrated hESC-RPE were enucleated and pigmented areas including the neuroretina, choroid and RPE layer, were dissected out, trimmed and mixed together in 500 µL of digestion buffer. Digestion buffer consisted of: 2mg/ml collagenase IV (ThermoFisher Scientific, 17104019), 120 U/µl DNase I (NEB, Sigma, 4536282001), and 1mg/ml papain (Sigma, 10108014001) in PBS. Eppendorfs containing the samples were rotated and incubated at 37°C on a thermocycler at 300g for 25 min until samples were homogenized. Samples were pipetted every 5 min to digest the tissue sample into single cells. Digestion was stopped by adding equivalent volume of 10% fetal bovine serum (ThermoFisher Scientific, 10082147) in PBS, the samples were filtered using a 30µm MACS Smart Strainer (Miltenyi, 130-098-458) followed by Dead Cell Removal kit (Miltenyi, 130-090-101) to remove dead cells and debris. At this stage, one of the rabbit eye cell samples was stained with mouse anti-human HLA-ABC-FITC (1:20, BD Biosciences, 555552, clone [G46-2.6]), and anti-human HLA-ABC-positive cells were FACS-sorted as specified above, collected and resuspended to 1000 cells/µL in 1% BSA (Sigma, A1470-10g) in PBS for further scRNA-seq. The rest of the samples were also resuspended to 1000 cells/µL in 1% BSA in PBS prior to scRNA-seq.

Acquisition of all primary human tissue samples from two pooled embryonic eyes at Carnegie Stages 12, 13, 14, and 15 (5 post-conception week embryo) was approved by the UCSF Human Gamete, Embryo and Stem Cell Research Committee (GESCR, approval 10-03379 and 10-05113). All experiments were performed in accordance with protocol guidelines. Informed consent was obtained before sample collection and use for

this study. First-trimester human samples were collected from elective pregnancy terminations through the Human Developmental Biology Resource (HDBR), staged using crown-rump length (CRL) and shipped overnight on ice in Rosewell Park Memorial Institute (RPMI) media. Dissections were based upon anatomical landmarks, and dissociations were performed using papain (Worthington). Samples were incubated in papain for 20-30 minutes and triturated manually into a single cell suspension. The samples were filtered for remaining debris and moved to PBS with 0.05% BSA to be captured by 10X Genomics Chromium RNA capture version 2. Library preparation was performed based upon manufacturer's instructions, and sequencing was performed on a NovaSeq S4 lane.

SINGLE-CELL RNA SEQUENCING

Specific stage hESC-RPE cells were trypsinized in TrypLE (10 min, 37°C, 5% CO_2), resuspended to 1000 cells/µL in 0.04% BSA in PBS, and transported at 4°C to the Eukaryotic Single Cell Genomics Facility (ESCG, SciLifeLab, Stockholm, Sweden) where a 3' cDNA library was prepared for scRNA-seq using the 10X Genomics platform and NovaSeq 6000 software. For in house scRNA-seq preparations, cells were collected with 0.04% BSA and counted with the automatized cell count Nucleocounter NC-200 (Chemometec) using the "Cell count and viability assay" and kept at 4°C for less than 1h until loaded onto the Chromium Next GEM Chip G. To process single samples, the Chromium Next GEM Single Cell 3' Reagent Kit v3.1 (Dual Index) was used (10x Genomics, CG000315). Some samples were multiplexed with CellPlex technology, in which cells first underwent the Cell Multiplexing Oligo Labeling Protocol (10x Genomics, CG000391) followed by protocol CB000388, Dual Index to integrate Cell Multiplexing samples. cDNA libraries were sequenced using Illumina Nextseq 2000 Platform 100 cycles P2 and Illumina Nextseq 550 High Output kit v2.5 (150 Cycles).

Cell Ranger 3.1.0 was used to convert Illumina base call files to FASTQ format and to map sequencing reads to the human GRCh38 reference transcriptome with the STAR aligner and generate feature-barcode count matrices. For the E1C3 cell line sequenced at Novo, Cellranger version 3.0.2 was used to demultiplex Illumina base calls and the resulting reads (FASTQ files) were mapped to the Human GRCh38 reference

transcriptome, Ensembl release 90 limited to protein coding genes and lincRNAs. For some older samples, Illumina base call files were converted to FASTQ format using Cell Ranger 2.1.1. Feature-barcode count matrices for all scRNA-seq data are available on GEO under accession number GSE164092. For samples on which RNA velocity was performed, the *velocyto run10x* command was used to produce loom files containing spliced and unspliced RNA information. Dataset quality control, normalization, dimensionality reduction, and visualization were performed using the *scanpy* and *velocyto* modules (La Manno et al., 2018; Wolf et al., 2018). Processed metadata files (h5ad/loom formats) are available on GEO. Jupyter notebooks to reproduce the analysis described below (both ipynb and html formats) are available for download, along with custom, documented source code, at https://github.com/lamanno-epfl. An overview of all scRNA-seq samples obtained in this study can be found in **Table S1**.

scRNA-seq PROCESSING for hESC-RPE *in vitro* DIFFERENTIATION

The initial number of barcoded cells across three cell lines (HS980, KARO1, and E1C3) was 31,879 cells from 19 individual samples (1,790 hESCs, 3,923 cells at D7, 3,453 cells at D14, 4,733 cells at D30, 5,826 cells at D38, 5,587 cells at D45, and 6,567 cells at D60). As a quality-control, cells were filtered based on the UMI count, number of uniquely expressed genes, and percentage of mitochondrial reads according to sample-specific criteria determined by the sequencing depth and coverage, outlined for all samples in **Table S1**. QC filtering resulted in 26,615 single cells: 11,791 HS980 cells (1,016 hESCs, 1,811 at D7, 1,872 at D14, 1,852 at D30, 1,784 at D38, 1,707 at D45, and 1,749 at D60), 6,983 KARO1 cells (885 at D7, 463 at D14, 841 at D30, 1,920 at D38, 1,638 at D45, and 1,236 at D60), and 7,841 E1C3 cells (321 at D7, 180 at D14, 1,153 at D30, 1,602 at D38, 1,793 at D45, and 2,792 at D60). Additional replicates of HS980 (3 total replicates) and E1C3 cell lines (4 total replicates) at D60 yielded 60,593 additional single cells, for a total of 87,208 single cells along the standard 2D monolayer differentiation protocol.

All scRNA-seq samples were first analyzed individually to obtain a UMAP representation and clustered using the standard parameters for normalization, PCA, UMAP, and Louvain clustering from the *scanpy* and *velocyto* packages (La Manno et al., 2018; Wolf et al., 2018). For global analysis of all three lines and seven time points, CCA

integration using a Python implementation of Seurat's integration method was applied to remove batch effects between lines (Butler et al., 2018). Source code and documentation for this custom Python CCA implementation is available at https://github.com/lamanno-epfl. Cell cycle phase assignments were inferred using the function *scanpy.score_genes_cell_cycle* on normalized counts and with published signature genes (Satija et al., 2015; Wolf et al., 2018). Global dimensionality reduction of the entire dataset using 2,000 cv-mean genes was performed using PCA, and the first two principal components derived were visualized. Signature score analysis was performed using a profile of well-studied pluripotency (6 genes: *SOX2, POU5F1, NANOG, ZFP42, LIN28A, SALL4*), retinal progenitor (10 genes: *RAX, OTX2, ZIC2, PAX6, SIX3, SIX6, LHX2, SFRP2, CRABP1, VSX2*) and RPE (15 genes: *TMEFF2, SERPINF1, MITF, PMEL, DCT, ELN, TYRP1, TYR, RLBP1, BEST1, RPE65, TTR, RGR, SFRP5, SLC6A13*) marker genes (Bosze et al., 2020; Fuhrmann, 2010; Schmitt et al., 2009). Global scores for these three cell type identities were computed across all cells by scaling each gene (above) between 0 and 1 and summing total scaled expression for included markers. Pseudotime trajectory inference of differentiating retinal progenitors and RPE at D30 across three cell lines was computed using the *dpt* diffusion pseudotime function in *scanpy*. For the HS980 cell line, SCENIC transcription factor regulon analysis was performed using *pyscenic* and default parameters as described in the original study and subsequent protocol (Aibar et al., 2017; Van de Sande et al., 2020).

Cluster annotation was performed starting with an explorative step followed by an iterative literature check process. The explorative step consisted of extracting the most enriched genes in one cluster as opposed to the others; this is achieved by ranking genes per each cell type by their enrichment score, obtained using the *enrichment.py* code available https://github.com/lamanno-epfl. The iterative literature check process involved using this list to perform literature searches on the basis of which the identity of the cluster is evaluated. More specifically, starting from the top of the list, we searched the literature (using PubMed) for pairs or triplets of enriched genes, and the literature inspection led to cell type hypotheses. Enriched gene lists were also queried using EnrichR (Chen et al., 2013) and, for relevant cell types, benchmarked against existing single cell atlases (La Manno et al., 2021; Lukowski et al., 2019). We then proceeded by attempting to falsify

each of those hypotheses by querying for known markers of the specific cell type and by querying well known markers of related but different cell types. The final cluster annotation was assigned to the cell type hypothesis left standing after this process. The specificity of the gene expression sets that lead to the assignment with respect to the other clusters was evaluated globally by plotting a heatmap displaying all the marker genes by cluster, cell type, or batch (Bosze et al., 2020; Cajal et al, 2012; Chen et al, 2017; Cohen-Salmon et al, 1997, Crespo-Enriquez et al, 2012; Gitton et al, 2011; Kasberg et al, 2013; Kumamoto and Hanashima et al, 2017; Kwon et al, 2010; Lu et al., 2019; McLarren et al., 2003; Pan and Thomson, 2007; Qu et al, 2008; Seo et al, 2017; Soldatov et al, 2019; Tahayato et al, 2003; Yamada et al, 2003).

To evaluate the pluripotency status of all *in vitro* single cells along the standard monolayer differentiation protocol, we trained a random forest classifier (*sklearn.ensemble.RandomForestClassifier)* to assign cells to one of two classes: one "pluripotent" class based on our undifferentiated hESCs and an additional 9,661 stem cells from a recent study (Cuomo et al., 2020) and one "other" class based on our CS12, CS13, CS14, CS15, and W7.5 references as well as external adult RPE references (Quake and Sapiens Consortium, 2021; Voigt et al., 2019). 1,000 cv-mean-enriched genes were used for the classifier; 70% of cells were used for training and 30% for testing. We then scored all *in vitro* cells using this classifier and visualized, by cell line, differentiation day, and replicate, each cell's probability (from 0 to 1) of belonging to the "pluripotent" class.

scRNA-seq PROCESSING for EB 3D DIFFERENTIATION

The initial number of barcoded cells at D7, D14, and D28 of EB differentiation were 205, 1,437, and 1,401 single cells, respectively. As a quality-control, cells were filtered based on the UMI count, number of uniquely expressed genes, and percentage of mitochondrial reads according to sample-specific criteria determined by the sequencing depth and coverage, outlined for all samples in **Table S1**. QC filtering resulted in 181, 1,382, and 1,288 single cells at D7, D14, and D28, respectively. Samples were analyzed using standard procedures for PCA, UMAP, and Louvain clustering using *scanpy*. Signature z-scores for forebrain, midbrain, hindbrain, and rostral neural tube tissues were obtained

using *signatures.py* (available on https://github.com/lamanno-epfl) as well as the following signature genes obtained from and supported by comprehensive single cell atlases: Forebrain (*PAX6, SIX3, FEZF1, RAX, HESX1, LHX2, LHX5, FEZF2, EMX2, SHTN1, IRS4, ARX, OTX1, OTX2, DMRT3, DMRTA2, WNT8B, ZIC4, SFRP1, FOXD4, EMX1, NR2E1, SIX6, VAX2*), Midbrain (*EN1, EN2, PAX5, PAX8, HES3, FAM181B, WNT1, LMX1A, LMX1B, IRX1, FGF18, FLRT1, FGF17, SOX21, ASCL1, TAL2*), Hindbrain (*HOXB2, VGLL3, HOXA2, HOXB1, OLIG3, MSX1, MSX2, CASZ1, FST, TSHZ1*), RetProg (*RAX, OTX2, ZIC2, PAX6, SIX3, LHX2, SFRP2, CRABP1, VSX2*), CrNeCr (*FOXC1, FOXC2, TFAP2A, PITX1, PITX2, ALX1, OTX1, GATA3, FZD10, WNT1*), PrePlac (*SIX1, SIX4, EYA1, EYA2, IRX1, ITX2, ITX3, SOX11, PAX6, OTX2, SIX3, GATA2, GATA3*), and LatNeEp (*DLX5, DLX6, SP8, SP9, DLK1, DLX3, DLX4, SEMA3E*) (Hu et al., 2019; La Manno et al., 2021; Lukowski et al., 2019; Pijuan-Sala et al., 2019). Mapping of 2D monolayer D30 cells (transferred data) onto the 3D EB D28 space (reference data) was performed as follows: correlations were computed between all transferred and reference data points (cells) using the *cdist* function from *scipy.spatial.distance* (Virtanen et al., 2020). Then, the median position of each transferred data point on reference UMAP space was calculated using its five nearest neighbors, by correlation coefficient, in the reference data. Lastly, the function *np.random.normal* was used with mean noise of 0.1 to facilitate easier visualization of multiple transferred cells mapping close to one another on the reference space.

scRNA-seq PROCESSING for D30 SORTED, D60 SORTED, and NON-REPLATING EXPERIMENTS

The initial number of barcoded cells in HS980 NCAM1-High and CD140b-High populations post-sorting was the following: D30 CD140b-high-sorted (1,703 cells), D30 NCAM1-high-sorted (865 cells), D60 CD140b-high-sorted (1,068 cells), D60 NCAM1-high-sorted (1,224 cells), and D60 unsorted (additional replicate; 1,103 cells). As a quality-control, cells were filtered based on the UMI count, number of uniquely expressed genes, and percentage of mitochondrial reads according to sample-specific criteria determined by the sequencing depth and coverage, outlined for all samples in **Table S1**. QC filtering resulted in: D30 CD140b-high-sorted (1,486 cells), D30 NCAM1-high-sorted

(734 cells), D60 CD140b-high-sorted (987 cells), D60 NCAM1-high-sorted (1,106 cells), and D60 unsorted (additional replicate of 975 cells). Samples were analyzed using standard procedures for dimensionality reduction, gene enrichment, UMAP, and clustering using *scanpy*. At D30, CCA integration with *integration.py* (available on https://github.com/lamanno-epfl) was performed between the D30 NCAM1-high-sorted and CD140b-high-sorted samples and the original HS980 D30 unsorted sample, whereas at D60 an additional unsorted sample was obtained using CellPlex with the sorted populations and used in downstream analyses without batch effect correction. Dot plots were created using the *sc.pl.dotplot* function in *scanpy*. Similarly, the initial number of barcoded cells in HS980 D38 Non-Replated and D60 Non-Replated samples were 1,983 and 1,647 cells, respectively. After QC, there were approximately 1,432 D38 and 793 D60 cells used for downstream PCA, UMAP, and clustering with *scanpy*.

scRNA-seq PROCESSING for HUMAN REFERENCE TISSUES

From the embryonic optic vesicles/cups at Carnegie Stage (CS) 12, 13, 14, 15, a total of 9,409 single cells were analyzed (excluding blood cell contaminants) using standard parameters in *scanpy* (CS12: 3,922 cells; CS13: 2,535 cells; CS14: 1,188 cells; CS15: 1,764 cells). Gene enrichment scores were computed and applied to annotate the following cell clusters: RetProg (4,121 cells), MesCh (1,750 cells), RPE (1,512 cells), Other Neural (936 cells), OcSurEct (775 cells), ANR (119 cells), Lens (72 cells), CornEp (54 cells), Endo (40 cells), NrlRet (17 cells), and Immune (13 cells). Visualization of all four samples on a single UMAP was obtained by regressing out batch information in *scanpy* using the *sc.pp.regress_out* command.

2,742 single cells from two human embryonic eyes at week 7.5 (1,439 from eye 1 and 1,303 from eye 2) were analyzed using *velocyto* and *scanpy* after QC. Due to minimal batch effects between fetal eyes, *cv_vs_mean* selection, dimensionality reduction, and other downstream analyses were jointly performed without batch effect correction (3,000 CV-mean genes, 25 PCs, 50 nearest neighbors). Cells were assigned to clusters annotated using gene enrichment as follows: Neural Crest Derived (1,078 cells), Neural Progenitors (706 cells), Retinal Pigment Epithelium (258 cells), Early Ganglion (165 cells), Lens Progenitors (150 cells), Mid Ganglion (104 cells), Late Ganglion (88 cells), Surface

Epithelium (53 cells), Horizontal Cells (36 cells), Lens (35 cells), and Early Photoreceptors (22 cells). Blood cell contaminants were excluded (47 cells).

5,538 single cells from two human adult eyes (4,420 cells from eye 1 and 1,792 from eye 2) were analyzed using *velocyto* and *scanpy* after QC. Due to the limited batch effect between eyes, downstream analyses were performed on the joined expression matrix. To strengthen our reference and ensure accurate capture of all RPE cells, a subset of RPE and melanocyte cells from scRNA-seq in a prior study were included in downstream analysis (reprocessed and filtered according to identical parameters) (Voigt et al., 2019). These 263 additional cells were obtained from 8 samples by performing a clustering of the published, reprocessed counts and selecting cells expressing melanocyte marker *MLANA* or RPE marker *RPE65* (GEO accession number: GSE135922). Adult retinal cell types were annotated using known marker genes and genes with high enrichment scores as follows: Muller Glia (1,646 cells), Rod Photoreceptors (MALAT1-lo) (1,323 cells), Rod Photoreceptors (MALAT1-hi) (747 cells), Melanocytes (364 cells), Fibroblast (267 cells), Cone Photoreceptors (218 cells), Cone Bipolar (195 cells), Amacrine Ganglion (181 cells), Rod Bipolar-ON (169 cells), Corneal Epithelium (161 cells), Immune (136 cells), Retinal Pigment Epithelium (71 cells), and Smooth Muscle (60 cells).

scRNA-seq PROCESSING for NCAM1-High-derived *in vitro* NEURORETINAL PROGENITOR DIFFERENTIATION

NCAM1-High-derived cells from our alternative neuronal differentiation protocol were analyzed using *velocyto* and *scanpy* on 2,638 unfiltered single cells. Cells with UMIs (>6,000 and <100,000) and a low percentage of mitochondrial reads (<10%) were retained during filtering. After dimensionality reduction and UMAP analysis (3,000 CV-mean genes, 20 PCs, 20 nearest neighbors), as well as the removal of a cluster cell contaminant, we obtained 980 single cells of the following clusters, upon which we performed gene enrichment: Mesenchyme (447 cells), Retinal Progenitors (134 cells), Retinal Pigment Epithelium (121 cells), Early Neuroblasts (102 cells), Neuroblasts (77 cells), Glutamatergic Neurons (46 cells), Gaba Neurons (25 cells), Corneal Epithelium (23 cells), and Lens (5 cells).

An integrated subspace was found between NCAM1-High-derived cells (980 cells) and human fetal eyes at week 7.5 (2,742 cells, see above) using CCA integration and 2,000 CV-mean genes for each dataset (3,063 unique genes in total). Gene enrichment scores were computed to identify unique and shared markers between the various populations, including overlapping lens and surface epithelium clusters. RNA velocity was performed on NCAM1-High-derived neurons (Early Neruoblast, Neuroblast, and Glutamatergic Neuron clusters) and fetal retinal ganglion cells (Early, Mid, and Late Ganglion clusters) with *velocyto*.

To compare the differentiation programs of NCAM1-High-derived *in vitro* neurons and fetal retinal ganglion neurons, we fit a principal curve to the two-dimensional UMAP embedding and identified a pseudotemporal cell ordering with which we could perform pseudotime gene expression alignment analysis. Principal curve code is adapted from the original publication and can be found at https://github.com/lamanno-epfl (Hastie and Stuetzle, 1989). Along each pseudotime, a set of five normally distributed and spaced curves were generated using scipy.stats.norm.pdf to mimic different possible expression patterns along the differentiation trajectory (i.e. early downregulation, upregulation midway through the pseudotime followed by downregulation, upregulation at the end of the pseudotime, etc.). Pearson's correlation coefficients were computed between genes and each normally distributed peak in order to identify genes with a distinct temporal upregulation and/or downregulation. For *in vitro* neurons and fetal neurons, the top 50 genes for each of five peaks were combined and compared, enabling us to visualize genes with a similar expression behavior along both pseudotimes as well as genes with a distinct expression behavior unique to either *in vitro* neurons or fetal neurons alone.

scRNA-seq PROCESSING for hESC-RPE *in vivo* RABBIT SUBRETINAL INJECTION

Unlike the other scRNA-seq data in this study, feature-barcode count matrices for hESCs isolated from rabbit retina were generated using a custom-built hybrid reference transcriptome combining both the human GRCh38 reference and the rabbit reference (Oryctolagus cuniculus 2.0.99, EMBL-EBI). FASTQ files were mapped to this hybrid reference with Cell Ranger 3.1.0 to generate count matrices. Reads were exclusively assigned either to a human or rabbit transcript. The percentage of UMIs per cell assigned

to the human or rabbit reference was then computed. Human cells were retained if >80% reads mapped to the human genome. Rabbit cells were retained if >95% reads mapped to the rabbit genome. The resulting number of cells were 24 human cells from the unsorted approach (0.8% of total, Rabbit1, unsorted) and 61 human cells by sorted approach (4.3% of total, Rabbit 2, sorted). As a control, feature-barcode count matrices were also created by aligning FASTQ files solely to the human or rabbit genome with Cell Ranger.

For analysis of human cells, cells with >30% reads mapped to mitochondrial genes were removed, resulting in a total of 65 single cells. Counts were size normalized in scanpy and gene feature selection was performed to select 100 variable genes using cv_vs_mean. Following log-transformation of counts, expression of RPE marker genes and other highly enriched genes was assessed. Differentiation expression analysis was performed by computing fold change on log2 normalized counts.

For analysis of rabbit retina, only cells obtained from the unsorted approach were used in order to avoid any potential cell-type sorting bias. Cells were filtered to retain those with a certain number of UMIs (>2,000 to <50,000), uniquely expressed genes (>600 to <6,000) and mitochondrial reads (<10%). The total number of cells after filtering was 1,965 cells. Dimensionality reduction and clustering analysis was performed using 3,000 cv_vs_mean selected genes and the workflow described above. Cell types were annotated using known marker genes and genes with high enrichment scores as follows: Fibroblast (408 cells), Rod Photoreceptors (338 cells), Smooth Muscles (200 cells), Muller Glia (182 cells), Amacrine Ganglion (156 cells), Rod Bipolar-ON (139 cells), Corneal Epithelium (125 cells), Cone Bipolar-OFF (119 cells), Cone Bipolar-ON (105 cells), Cone Photoreceptors (89 cells), Immune (54 cells), Melanocytes (41 cells), and Retinal Pigment Epithelium (9 cells). For integration of the human adult eye and rabbit eye, anchors were found only between genes with shared nomenclature between the two species' genomes (9,889 genes) and the top enriched 2,000 genes (2,979 genes in total).

GENE ENRICHMENT and SIGNATURE SCORE ANALYSIS

Throughout this study, all marker genes for visualized cell type clusters were selected from among the top 50 ranked genes calculated with a gene enrichment score from

enrichment.py. For each gene, the mean value per cluster was obtained and scaled by the number of cells per cluster with non-zero counts of the given gene. A gene-wise enrichment score was then computed by comparing the means and the fraction of non-zero values among all clusters. Likewise, signature z-scores were computed using a selection of top ranked enriched genes against a randomized set of background gene expression using *signatures.py*. Both custom source codes can be downloaded at https://github.com/lamanno-epfl/rpe_differentiation_profiling_code along with example usage in our Jupyter notebook references (available in both ipynb and html formats).

CELL HETEROGENEITY ESTIMATION

When decomposing the covariance of a heterogeneous cell population, the largest axes of variation typically explain cell type variability, whereas the smaller axes describe subtle phenotypic variation and uncorrelated noise accumulating on the remaining principal components. We deduced that the area under the curve (AUC) of cumulative principal component variance would serve as an insightful metric to assess the overall heterogeneity at each differentiation day. For each time point, *cv_vs_mean* feature selected genes with a score greater than 0.30 were collected, and a union of genes from all time points were retained and size normalized. 800 cells and half the total number of genes were randomly sub-sampled from each time point, without replacement, and PCA analysis was performed. AUC of the cumulative explained variance ratio for all principal components was calculated using *numpy.trapz*. Cells and genes were randomly subsampled for 1000 iterations to obtain a distribution of AUCs at each differentiation day. As a greater deviation from the variance due to biological noise results in a greater AUC of cumulative explained variance, the AUC can therefore be used as a proxy for assessing the overall dataset heterogeneity.

CANONICAL CORRELATION ANALYSIS and DATA INTEGRATION

Canonical correlation analysis (CCA) was performed in all cases using a Python adaptation of the original Seurat method and is available to download at our GitHub repository: https://github.com/lamanno-epfl/ (Butler et al., 2018). For integration of the *in vitro* hESC-RPE D7 and D14 time points, both with and without the cell cycle regressed

out, anchors were found using the top enriched 1,000 genes at each time point obtained with cv_vs_mean in velocyto. For integration of the human fetal week 7.5 and NCAM1-High-derived neuronal hESCs, anchors were found using the top enriched 1,000 genes.

RPE CORRELATION ANALYSIS

We reasoned that reliable retinal progenitor markers would be inversely correlated to genes characterizing more mature cells, such as RPE, and to progenitors for other neuroepithelium tissues. To identify genes strongly linked to a progenitor fate among *in vitro* hESC-RPE D30 cells, Pearson's correlation coefficients were calculated between log-normalized gene counts and either an RPE signature score (16 genes, described above) or a neural tube signature (17 genes: *WNT6*, *COL17A1*, *CDH1*, *TP63*, *KRT19*, *KRT17*, *CRABP2*, *COL3A1*, *CYP26A1*, *FOXC1*, *HAND1*, *SEMA3D*, *SOX17*, *DLX2*, *DLX3*, *DLX4*, *PDGFRA*). For the RPE correlation score, D30 cells from RetProg and RPE secondary clusters were used and for the neural tube correlation score, D30 cells from RPE and CrNeCr secondary clusters were used. Genes with an average spliced expression <0.5 as well as genes included in the signature scores themselves were excluded, resulting in the calculation of RPE signature correlation coefficients for 5,214 genes and of neural signature correlation coefficients for 4,664 genes. Both correlation coefficients were then averaged to obtain the top genes anticorrelated to both signatures and therefore most indicative of a cell progenitor status. Some of these genes possessed known links to anterior placode and epithelium development (Imuta et al, 2009; Yamada et al, 2003; Zhou et al, 2010). The computed correlation coefficients are available in **Table S4**.

RNA VELOCITY ANALYSIS

RNA velocity analysis was performed using *velocyto* and *scvelo,* for dimensionality reduction, KNN smoothing, and gamma fitting we used default parameters unless specified (Bergen et al., 2019; La Manno et al., 2018). For fetal retinal ganglion neurons and NCAM1-High-derived neuronal hESCs, the steady-state implementation of RNA velocity was used (2,000 enriched genes, 20 PCs, 20 nearest neighbors). For estimating RNA velocity of selected *in vitro* hESC-RPE D60 cells, 1,000 CV-mean genes were

selected for velocity estimation. To ensure capture of the appropriate genes, we selected genes with a highly coordinated velocity using a velocity coordination function adapted from velocyto and available at: https://github.com/lamanno-epfl/r. Phase portraits were computed on a gene-wise basis using normalized spliced and unspliced counts, represented on the x-axis and y-axis, respectively. A steady-state fit line was obtained by inferring the degradation rate (γ) according to the standard parameters in *velocyto* (La Manno et al., 2018).

ORDINAL CLASSIFIER

In order to assign *in vitro* RPE cells to an embryonic reference, we constructed an ordinal classifier function in Python and trained it using scRNA-seq data of 783 embryonic RPE and retinal progenitor cells from weeks 5 to 24 of development (Hu et al., 2019) as well as 176 adult RPE reference cells (Voigt et al., 2019). This classifier is based on prior work and relies on training a series of sequential classifiers representing temporally ordered stages of development (Frank and Hall, 2001). Sequential classes were designed by grouping RPE scRNA-seq gene expression training data by similar stages as follows: weeks 5-6 (both retinal progenitors and early RPE), weeks 7-9, weeks 11-13, weeks 17-24, and adult RPE). Classification was performed using log-transformed, size-normalized counts on 2,000 cv-mean enriched genes, excluding mitochondrial and blood-enriched contaminant markers. Results of the classifier were either visualized as scatter plots of each individual cell's predicted ordinal class using *matplotlib.pyplot.scatter* or as kernel density plots averaging a trend for all single classifier cells in a particular sample using the *sklearn.neighbors.KernelDensity* function. To verify the robustness of the classifier, retinal progenitor and RPE cells from our CS13 (W5) and CS20 (W7.5) datasets were classified, as well as 49 additional adults RPE cells from an additional study not used in training of the classifier (Quake and Sapiens Consortium, 2021). The implementation is available as *ordinal_classifier.py* at our GitHub page: https://github.com/lamanno-epfl/.

BIOINFORMATICS SOFTWARE

All analysis was performed using Python 3.7.4. The following modules were used: jupyterlab 1.1.4, loompy 3.0.6, louvain 0.6.1, matplotlib 3.3.1, numpy 1.19.1, pandas

0.25.1, pyscenic 0.10.4, python-igraph 0.7.1, python-louvain 0.13, scanpy 1.4.5, scikit-learn 0.23.2, scipy 1.5.2, scvelo 0.1.25, seaborn 0.9.0, umap-learn 0.4.0, velocyto 0.17.17.

STATISTICAL ANALYSIS

For statistical analysis, one-way ANOVA and posthoc multiple comparisons using Tukey's test correction were performed to assess the *in vitro* differences of the sorted (NCAM1-High, CD140b-High) and unsorted D30 cells in TEER and PEDF secretion assays. Standard error of the mean and standard deviation calculations for all single cell analyses were performed using the *numpy* package in Python across replicates from three cell lines.

REFERENCES

Aibar, S., González-Blas, C.B., Moerman, T., Huynh-Thu, V.A., Imrichova, H., Hulselmans, G., Rambow, F., Marine, J.-C., Geurts, P., Aerts, J., et al. (2017). SCENIC: single-cell regulatory network inference and clustering. Nat. Methods 14, 1083–1086.

Bartuma, H., Petrus-Reurer, S., Aronsson, M., Westman, S., André, H., and Kvanta, A. (2015). In Vivo Imaging of Subretinal Bleb-Induced Outer Retinal Degeneration in the Rabbit. Invest. Ophthalmol. Vis. Sci. 56, 2423–2430.

Bergen, V., Lange, M., Peidli, S., Alexander Wolf, F., and Theis, F.J. (2019). Generalizing RNA velocity to transient cell states through dynamical modeling.

Bosze, B., Hufnagel, R.B., and Brown, N.L. (2020). Chapter 21 - Specification of retinal cell types. In Patterning and Cell Type Specification in the Developing CNS and PNS (Second Edition), J. Rubenstein, P. Rakic, B. Chen, and K.Y. Kwan, eds. (Academic Press), pp. 481–504.

Butler, A., Hoffman, P., Smibert, P., Papalexi, E., and Satija, R. (2018). Integrating single-cell transcriptomic data across different conditions, technologies, and species. Nat. Biotechnol. 36, 411–420.

Cajal, M., Lawson, K.A., Hill, B., Moreau, A., Rao, J., Ross, A., Collignon, J., and Camus, A. (2012). Clonal and molecular analysis of the prospective anterior neural boundary in the mouse embryo. Development 139, 423–436.

Chen, E.Y., Tan, C.M., Kou, Y., Duan, Q., Wang, Z., Meirelles, G.V., Clark, N.R., and Ma'ayan, A. (2013). Enrichr: interactive and collaborative HTML5 gene list enrichment analysis tool. BMC Bioinformatics 14, 128.

Chen, J., Tambalo, M., Barembaum, M., Ranganathan, R., Simões-Costa, M., Bronner, M.E., and Streit, A. (2017). A systems-level approach reveals new gene regulatory modules in the developing ear. Development 144, 1531–1543.

Cohen-Salmon, M., El-Amraoui, A., Leibovici, M., and Petit, C. (1997). Otogelin: a glycoprotein specific to the acellular membranes of the inner ear. Proc. Natl. Acad. Sci. U. S. A. 94, 14450–14455.

Crespo-Enriquez, I., Partanen, J., Martinez, S., and Echevarria, D. (2012). Fgf8-Related Secondary Organizers Exert Different Polarizing Planar Instructions along the Mouse Anterior Neural Tube. PLoS One 7, e39977.

Cuomo, A.S.E., Seaton, D.D., McCarthy, D.J., Martinez, I., Bonder, M.J., Garcia-Bernardo, J., Amatya, S., Madrigal, P., Isaacson, A., Buettner, F., et al. (2020). Single-cell RNA-sequencing of differentiating iPS cells reveals dynamic genetic effects on gene expression. Nat. Commun. 11, 810.

David, F.P.A., Litovchenko, M., Deplancke, B., and Gardeux, V. (2020). ASAP 2020 update: an open, scalable and interactive web-based portal for (single-cell) omics analyses. Nucleic Acids Res. 48, W403–W414.

Frank, E., and Hall, M. (2001). A Simple Approach to Ordinal Classification. In Machine Learning: ECML 2001, (Springer Berlin Heidelberg), pp. 145–156.

Fuhrmann, S. (2010). Eye morphogenesis and patterning of the optic vesicle. Curr. Top. Dev. Biol. 93, 61–84.

Gardeux, V., David, F.P.A., Shajkofci, A., Schwalie, P.C., and Deplancke, B. (2017). ASAP: a web-based platform for the analysis and interactive visualization of single-cell RNA-seq data. Bioinformatics 33, 3123–3125.

Gitton, Y., Benouaiche, L., Vincent, C., Heude, E., Soulika, M., Bouhali, K., Couly, G., and Levi, G. (2011). Dlx5 and Dlx6 expression in the anterior neural fold is essential for patterning the dorsal nasal capsule. Development 138, 897–903.

Hastie, T., and Stuetzle, W. (1989). Principal Curves. J. Am. Stat. Assoc. 84, 502–516.

Hu, Y., Wang, X., Hu, B., Mao, Y., Chen, Y., Yan, L., Yong, J., Dong, J., Wei, Y., Wang, W., et al. (2019). Dissecting the transcriptome landscape of the human fetal neural retina and retinal pigment epithelium by single-cell RNA-seq analysis. PLoS Biol. 17, e3000365.

Imuta, Y., Nishioka, N., Kiyonari, H., and Sasaki, H. (2009). Short limbs, cleft palate, and delayed formation of flat proliferative chondrocytes in mice with targeted disruption of a putative protein kinase gene, Pkdcc (AW548124). Dev. Dyn. 238, 210–222.

Joshi, R., Mankowski, W., Winter, M., Saini, J.S., Blenkinsop, T.A., Stern, J.H., Temple, S., and Cohen, A.R. (2016). Automated Measurement of Cobblestone Morphology for Characterizing Stem Cell Derived Retinal Pigment Epithelial Cell Cultures. J. Ocul. Pharmacol. Ther. 32, 331–339.

Kasberg, A.D., Brunskill, E.W., and Steven Potter, S. (2013). SP8 regulates signaling centers during craniofacial development. Dev. Biol. 381, 312–323.

Kumamoto, T., and Hanashima, C. (2017). Evolutionary conservation and conversion of Foxg1 function in brain development. Dev. Growth Differ. 59, 258–269.

Kwon, H.-J., Bhat, N., Sweet, E.M., Cornell, R.A., and Riley, B.B. (2010). Identification of early requirements for preplacodal ectoderm and sensory organ development. PLoS Genet. 6, e1001133.

La Manno, G., Soldatov, R., Zeisel, A., Braun, E., Hochgerner, H., Petukhov, V., Lidschreiber, K., Kastriti, M.E., Lönnerberg, P., Furlan, A., et al. (2018). RNA velocity of single cells. Nature 560, 494–498.

La Manno, G., Siletti, K., Furlan, A., Gyllborg, D., Vinsland, E., Mossi Albiach, A., Mattsson Langseth, C., Khven, I., Lederer, A.R., Dratva, L.M., et al. (2021). Molecular architecture of the developing mouse brain. Nature 596, 92–96.

Lidgerwood, G.E., Senabouth, A., Smith-Anttila, C.J.A., Gnanasambandapillai, V., Kaczorowski, D.C., Amann-Zalcenstein, D., Fletcher, E.L., Naik, S.H., Hewitt, A.W., Powell, J.E., et al. (2021). Transcriptomic Profiling of Human Pluripotent Stem Cell-derived Retinal Pigment Epithelium over Time. Genomics Proteomics Bioinformatics 19, 223–242.

Lu, Y., Shiau, F., Yi, W., Lu, S., Wu, Q., Pearson, J.D., Kallman, A., Zhong, S., Hoang, T., Zuo, Z., et al. (2020). Single-Cell Analysis of Human Retina Identifies Evolutionarily Conserved and Species-Specific Mechanisms Controlling Development. Dev. Cell 53, 473–491.e9.

Lukowski, S.W., Lo, C.Y., Sharov, A.A., Nguyen, Q., Fang, L., Hung, S.S., Zhu, L., Zhang, T., Grünert, U., Nguyen, T., et al. (2019). A single-cell transcriptome atlas of the adult human retina. EMBO J. 38, e100811.

McLarren, K.W., Litsiou, A., and Streit, A. (2003). DLX5 positions the neural crest and preplacode region at the border of the neural plate. Dev. Biol. 259, 34–47.

Pan, G., and Thomson, J.A. (2007). Nanog and transcriptional networks in embryonic stem cell pluripotency. Cell Res. 17, 42–49.

Petrus-Reurer, S., Bartuma, H., Aronsson, M., Westman, S., Lanner, F., André, H., and Kvanta, A. (2017). Integration of Subretinal Suspension Transplants of Human Embryonic Stem Cell-Derived Retinal Pigment Epithelial Cells in a Large-Eyed Model of Geographic Atrophy. Investigative Opthalmology & Visual Science 58, 1314.

Petrus-Reurer, S., Bartuma, H., Aronsson, M., Westman, S., Lanner, F., and Kvanta, A. (2018). Subretinal Transplantation of Human Embryonic Stem Cell Derived-retinal Pigment Epithelial Cells into a Large-eyed Model of Geographic Atrophy. J. Vis. Exp. I 131, 56702.

Pijuan-Sala, B., Griffiths, J.A., Guibentif, C., Hiscock, T.W., Jawaid, W., Calero-Nieto, F.J., Mulas, C., Ibarra-Soria, X., Tyser, R.C.V., Ho, D.L.L., et al. (2019). A single-cell molecular map of mouse gastrulation and early organogenesis. Nature 566, 490–495.

Plaza Reyes, A., Petrus-Reurer, S., Antonsson, L., Stenfelt, S., Bartuma, H., Panula, S., Mader, T., Douagi, I., André, H., Hovatta, O., et al. (2016). Xeno-Free and Defined Human Embryonic Stem Cell-Derived Retinal Pigment Epithelial Cells Functionally Integrate in a Large-Eyed Preclinical Model. Stem Cell Reports 6, 9–17.

Plaza Reyes, A., Petrus-Reurer, S., Padrell Sanchez, S., Kumar, P., Douagi, I., Bartuma, H., Aronsson, M., Westman, S., Lardner, E., Andre, H., et al. (2020a). Identification of cell

surface markers and establishment of monolayer differentiation to retinal pigment epithelial cells. Nat. Commun. 11, 1609.

Plaza Reyes, A., Petrus-Reurer, S., Sánchez, S.P., Kumar, P., Douagi, I., Bartuma, H., Aronsson, M., Westman, S., Lardner, E., Falk, A., et al. (2020b). Xeno-free, chemically defined and scalable monolayer differentiation protocol for retinal pigment epithelial cells. PROTOCOL (Version 1) available at Protocol Exchange [https://doi.org/10.21203/rs.3.pex-635/v1].

Qu, X.-B., Pan, J., Zhang, C., and Huang, S.-Y. (2008). Sox17 facilitates the differentiation of mouse embryonic stem cells into primitive and definitive endoderm in vitro. Dev. Growth Differ. 50, 585–593.

Quake, S.R., and Sapiens Consortium, T. (2021). The Tabula Sapiens: a single cell transcriptomic atlas of multiple organs from individual human donors. Biorxiv.

Rodin, S., Antonsson, L., Niaudet, C., Simonson, O.E., Salmela, E., Hansson, E.M., Domogatskaya, A., Xiao, Z., Damdimopoulou, P., Sheikhi, M., et al. (2014). Clonal culturing of human embryonic stem cells on laminin-521/E-cadherin matrix in defined and xeno-free environment. Nat. Commun. 5, 3195.

Satija, R., Farrell, J.A., Gennert, D., Schier, A.F., and Regev, A. (2015). Spatial reconstruction of single-cell gene expression data. Nat. Biotechnol. 33, 495–502.

Schmitt, S., Aftab, U., Jiang, C., Redenti, S., Klassen, H., Miljan, E., Sinden, J., and Young, M. (2009). Molecular characterization of human retinal progenitor cells. Invest. Ophthalmol. Vis. Sci. 50, 5901–5908.

Sofroniew, N., Lambert, T., Evans, K., Nunez-Iglesias, J., Bokota, G., Peña-Castellanos, G., Winston, P., Yamauchi, K., Bussonnier, M., Pop, D.D., et al. (2021). napari/napari: 0.4.12rc2.

Soldatov, R., Kaucka, M., Kastriti, M.E., and Petersen, J. (2019). Spatiotemporal structure of cell fate decisions in murine neural crest. Science. 2019 Jun 7;364(6444):eaas9536.

Seo, S., Chen, L., Liu, W., Zhao, D., Schultz, K.M., Sasman, A., Liu, T., Zhang, H.F., Gage, P.J., and Kume, T. (2017). Foxc1 and Foxc2 in the Neural Crest Are Required for Ocular Anterior Segment Development. Invest. Ophthalmol. Vis. Sci. 58, 1368–1377.

Tahayato, A., Dollé, P., and Petkovich, M. (2003). Cyp26C1 encodes a novel retinoic acid-metabolizing enzyme expressed in the hindbrain, inner ear, first branchial arch and tooth buds during murine development. Gene Expr. Patterns 3, 449–454.

Van de Sande, B., Flerin, C., Davie, K., De Waegeneer, M., Hulselmans, G., Aibar, S., Seurinck, R., Saelens, W., Cannoodt, R., Rouchon, Q., et al. (2020). A scalable SCENIC workflow for single-cell gene regulatory network analysis. Nat. Protoc. 15, 2247–2276.

Virtanen, P., Gommers, R., Oliphant, T.E., Haberland, M., Reddy, T., Cournapeau, D., Burovski, E., Peterson, P., Weckesser, W., Bright, J., et al. (2020). SciPy 1.0: fundamental algorithms for scientific computing in Python. Nat. Methods 17, 261–272.

Voigt, A.P., Mulfaul, K., Mullin, N.K., Flamme-Wiese, M.J., Giacalone, J.C., Stone, E.M., Tucker, B.A., Scheetz, T.E., and Mullins, R.F. (2019). Single-cell transcriptomics of the human retinal pigment epithelium and choroid in health and macular degeneration. Proc. Natl. Acad. Sci. U. S. A. 116, 24100–24107.

Wolf, F.A., Angerer, P., and Theis, F.J. (2018). SCANPY: large-scale single-cell gene expression data analysis. Genome Biol. 19, 15.

Yamada, R., Mizutani-Koseki, Y., Hasegawa, T., Osumi, N., Koseki, H., and Takahashi, N. (2003). Cell-autonomous involvement of Mab21l1 is essential for lens placode development. Development 130, 1759–1770.

Zhou, J., Wang, C., Wang, Z., Dampier, W., Wu, K., Casimiro, M.C., Chepelev, I., Popov, V.M., Quong, A., Tozeren, A., et al. (2010). Attenuation of Forkhead signaling by the retinal determination factor DACH1. Proc. Natl. Acad. Sci. U. S. A. 107, 6864–6869.
