# Supplementary material for: Molecular profiling of stem cell-derived retinal pigment epithelial cell differentiation established for clinical translation
Source: Stem Cell Reports. 2022 Jun 14;17(6):1458–75. doi: 10.1016/j.stemcr.2022.05.005 (PMC9214069; doi:10.1016/j.stemcr.2022.05.005)
Supplement: Document S2. Article plus supplemental information [file mmc8.pdf]

Stem Cell Reports
Article

OPEN ACCESS

Molecular profiling of stem cell-derived retinal pigment epithelial cell differentiation established for clinical translation

Sandra Petrus-Reurer,[1,2,3,11] Alex R. Lederer,[4,11] Laura Baqué-Vidal,[1,2] Iyadh Douagi,[5] Belinda Pannagel,[5] Irina Khven,[4] Monica Aronsson,[3] Hammurabi Bartuma,[3] Magdalena Wagner,[1,2] Andreas Wrona,[6] Paschalis Efstathopoulos,[6] Elham Jaberi,[6] Hanni Willenbrock,[6] Yutaka Shimizu,[6] J. Carlos Villaescusa,[6] Helder André,[3] Erik Sundström,[7] Aparna Bhaduri,[8,9] Arnold Kriegstein,[8,9] Anders Kvanta,[3] Gioele La Manno,[4,*] and Fredrik Lanner[1,2,10,*]

[1]Department of Clinical Sciences, Intervention and Technology, Karolinska Institutet, 17177 Stockholm, Sweden
[2]Gynecology and Reproductive Medicine, Karolinska Universitetssjukhuset, 14186 Stockholm, Sweden
[3]Department of Clinical Neuroscience, Division of Eye and Vision, St. Erik Eye Hospital, Karolinska Institutet, 11282 Stockholm, Sweden
[4]Laboratory of Neurodevelopmental Systems Biology, Brain Mind Institute, School of Life Sciences, École Polytechnique Fédérale de Lausanne (EPFL), 1015 Lausanne, Switzerland
[5]Center for Hematology and Regenerative Medicine, Department of Medicine, Karolinska Institutet, 17177 Stockholm, Sweden
[6]Cell Therapy R&D, Novo Nordisk A/S, Måløv 2760, Denmark
[7]Department of Neurobiology, Care Sciences and Society, Karolinska Institutet, 17177 Stockholm, Sweden
[8]Department of Neurology, University of California, San Francisco, CA, USA
[9]Eli and Edythe Broad Center for Regeneration Medicine and Stem Cell Research, University of California, San Francisco, CA, USA
[10]Ming Wai Lau Center for Reparative Medicine, Stockholm node, Karolinska Institutet, 17177 Stockholm, Sweden
[11]These authors contributed equally
*Correspondence: gioele.lamanno@epfl.ch (G.L.M.), fredrik.lanner@ki.se (F.L.)
https://doi.org/10.1016/j.stemcr.2022.05.005

SUMMARY

Human embryonic stem cell-derived retinal pigment epithelial cells (hESC-RPE) are a promising cell source to treat age-related macular degeneration (AMD). Despite several ongoing clinical studies, a detailed mapping of transient cellular states during *in vitro* differentiation has not been performed. Here, we conduct single-cell transcriptomic profiling of an hESC-RPE differentiation protocol that has been developed for clinical use. Differentiation progressed through a culture diversification recapitulating early embryonic development, whereby cells rapidly acquired a rostral embryo patterning signature before converging toward the RPE lineage. At intermediate steps, we identified and examined the potency of an NCAM1$^+$ retinal progenitor population and showed the ability of the protocol to suppress non-RPE fates. We demonstrated that the method produces a pure RPE pool capable of maturing further after subretinal transplantation in a large-eyed animal model. Our evaluation of hESC-RPE differentiation supports the development of safe and efficient pluripotent stem cell-based therapies for AMD.

INTRODUCTION

The eye, by virtue of its accessibility and isolated anatomical location, has emerged as a promising organ for gene- and cell-based therapies. A pathology that is particularly promising to tackle with these approaches is age-related macular degeneration (AMD), which causes severe vision loss and affects more than 180 million people globally (Gehrs et al., 2006). The dry form of the disease, for which no treatment is available, affects 80%–90% of advanced patients and is characterized by well-demarcated areas of retinal pigment epithelium (RPE) loss and retinal degeneration (Ambati et al., 2003; Sunness, 1999). Human pluripotent stem cell (hPSC)-derived RPE cells are thus of high interest for cell replacement treatment options, and currently are being tested in several clinical trials (Maeda et al., 2022).

Efforts have been made toward developing strategies to ensure high-purity RPE products, but focus on final product composition has overshadowed the characterization of intermediate stages appearing before a final steady state is reached (Choudhary and Whiting, 2016; Plaza Reyes et al., 2020a). Single-cell RNA sequencing (scRNA-seq) can systematically phenotype cell populations, and its genome-wide readout is crucial to explore *in vitro* differentiation (Kulkarni et al., 2019; Kumar et al., 2017; Lederer and La Manno, 2020). For example, scRNA-seq can determine whether cells follow developmental or non-canonical paths to maturation (Cuomo et al., 2020). Analyzing cell pools at intermediate stages might expose interesting relations between *in vitro* and *in vivo* processes and help to correctly identify potential risks for clinical translation (Begbie, 2013; La Manno et al., 2016). Comprehensive single-cell atlases of embryonic and postnatal neurodevelopment are also fundamental to assisting in the evaluation of gene expression profiles measured *in vitro* (La Manno et al., 2021; Zeisel et al., 2018). Recent work has sought to decompose cellular heterogeneity of the embryonic and postnatal eye with scRNA-seq, but the similarity between transient states arising in development and human pluripotent stem cell (hPSC)-derived intermediates en route to RPE

This is an open access article under the CC BY license (http://creativecommons.org/licenses/by/4.0/).

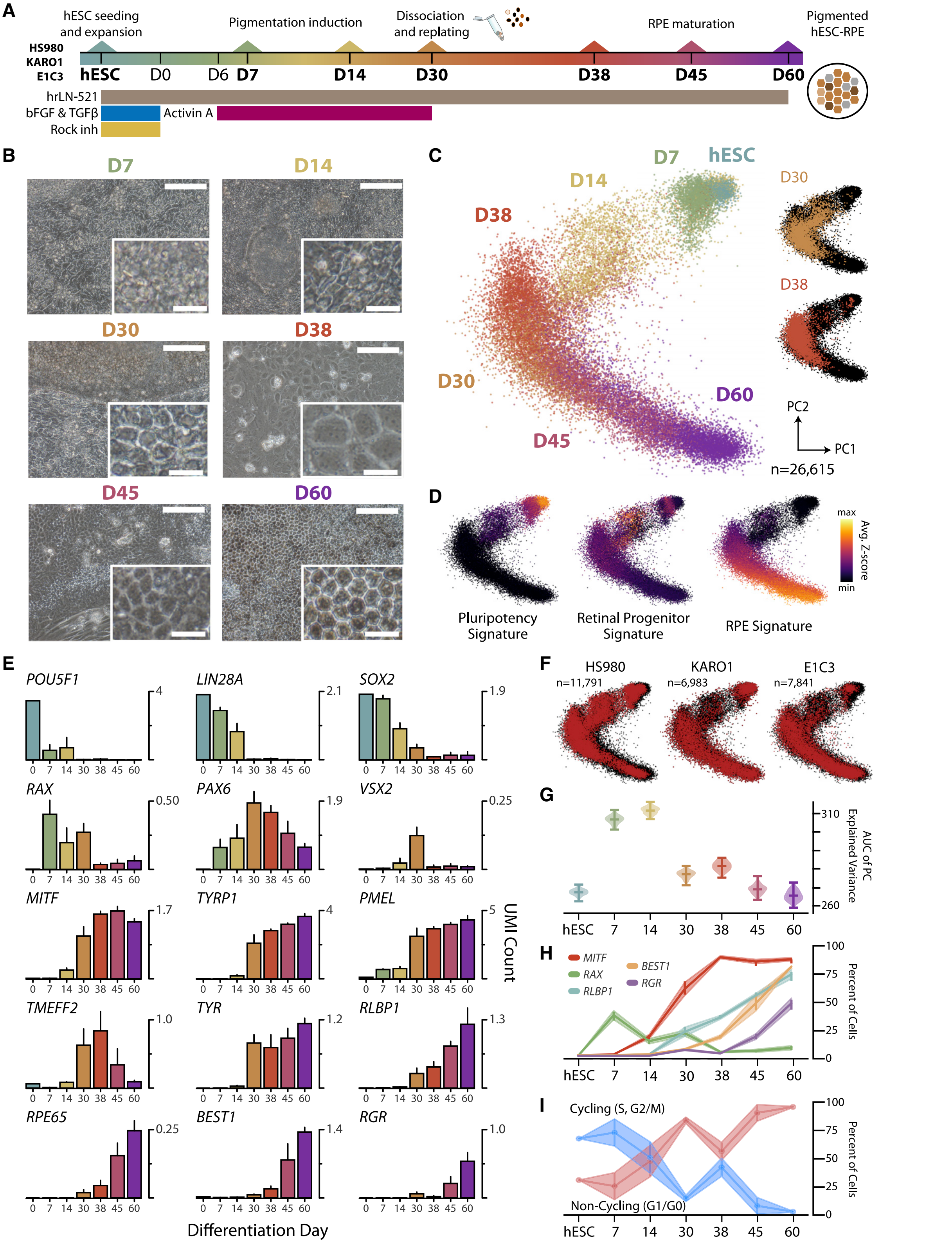

(legend on next page)

lineage has not yet been evaluated (Hu et al., 2019; Lidgerwood et al., 2021; Lukowski et al., 2019; Voigt et al., 2019).

In this study, we performed scRNA-seq analyses during human embryonic stem cell RPE (hESC-RPE) differentiation using a protocol established for clinical translation (Plaza Reyes et al., 2020a, 2020b). We demonstrate that cells follow embryonic retinal specification, reaching a mature, pigmented RPE phenotype and even undergoing further maturation toward an adult-like state upon subretinal transplantation into the albino rabbit eye. These findings provide valuable insight into the developmental program of hESC-RPE differentiation and illustrate the required high quality of the derived cells to be used as a future certified clinical product.

RESULTS

Human embryonic stem cells traverse gene expression space and sequentially mature into retinal pigment epithelium

To examine the process by which hESC-RPE are generated (Plaza Reyes et al., 2020a, 2020b), we performed scRNA-seq throughout differentiation (Figure 1A). We profiled differentiation of one research and two clinical grade cell lines (HS980, KARO1, and E1C3, respectively) at six time points (day 7 [D7], D14, D30, D38, D45, and D60; Table S1). Morphological evaluation using cobblestone junction scores confirmed that changes in cell shape and size followed differentiation as cells progressively assumed a tighter cobblestone monolayer of pigmented cells (Joshi et al., 2016) (Figures 1B, S1A, and S1B).

Assessment of 26,615 single-cell transcriptomes showed that cells traversed a reduced gene expression space from the pluripotent state toward a mature RPE identity (Figures 1C, S1C, and S1D). Gene signature scores detected an initial loss of the pluripotency signature, increased progenitor status at intermediate days, and a later rise of mature RPE (Figures 1D and S1E). Temporal assessment of gene expression confirmed distinct expression waves, with pluripotency genes (*POU5F1*, *LIN28A*, *SOX2*) leading and being downregulated in favor of progenitor genes (*RAX*, *PAX6*, *VSX2*), eventually trailed by early (*MITF*, *TYRP1*, *PMEL*, *TMEFF2*), intermediate (*TYR*, *RLBP1*), and late (*RPE65*, *BEST1*, *RGR*) RPE maturation genes (Figure 1E). Cells from all three lines were uniformly distributed along the global representation, demonstrating robustness and reproducibility of the protocol through a path consistent with the intended differentiation (Figure 1F).

Heterogeneity analysis reveals changes in cell diversity during differentiation

Interestingly, we observed deviations from a uniform progression toward RPE. D30 cells appeared more morphologically differentiated toward RPE than D38 cells, likely a response to dissociating and replating (Figures 1C and S1A–S1E). A subset of intermediate cells did not exhibit a strong signature for any of the three global identities considered, suggesting a complex differentiation process and presence of additional cell types (Figure 1D).

To quantify the biological heterogeneity observed, we calculated the variance accumulated in correlated gene modules (see supplemental experimental procedures and Figure S1F). This revealed that initial (hESCs) and endpoint (D60) cells harbored a lower heterogeneity compared to intermediate days (Figure 1G). While a large decrease in heterogeneity was detected from D14 to D30, suggesting an initial convergence toward RPE fate, we observed a slight increase from D30 to D38, thus hinting at an effect of cell dissociation, replating, or Activin A removal on cell composition. A similar pattern was observed with cobblestone junction scores (Figure S1A). Initial and endpoint samples also had mutually exclusive and uniform expression of pluripotency and RPE genes (Figures 1H and S1G–S1I). This was consistent with proliferation trends: a decreased fraction of cycling cells from hESC to D30, followed by an increase from D30 to D38, and finally a second decline from D38 to D60 (Figures 1I and S1J).

Figure 1. Global scRNA-seq characterization of hESC-RPE differentiation trajectory

(A) Schematic of the hESC-RPE differentiation experimental protocol where scRNA-seq was performed at the seven time points (bolded; D, day) in three cell lines: HS980, KARO1, and E1C3.

(B) Brightfield images during HS980 differentiation. Scale bars, 100 μm; inset scale bars, 20 μm.

(C) Principal component (PC) representation of 26,615 single cells across three lines using 2,000 cv-mean enriched genes.

(D) PC showing signature scores for pluripotency, retinal progenitors, and RPE cells.

(E) Bar graphs showing average normalized gene expression of pluripotent, retinal progenitor, and RPE markers in scRNA-seq data. Error bars represent standard deviation of the mean across three lines, except for the hESC time point.

(F) PC plot colored by cell line in red.

(G) Plot showing cumulative explained variance curve for each time point and all lines, applied to estimate how much variance accumulates over sets of correlated genes (biological-driven variability), as opposed to uniformly across genes (white noise).

(H) Line plots showing percentage of cells positive for retinal marker genes at each time point.

(I) Line plots showing scRNA-seq-based cell-cycle phase assignment. Cycling: S and G_2/M; non-cycling: G_1/G_0.

Intervals in (H) and (I) represent the 95% confidence intervals. See also Figure S1.

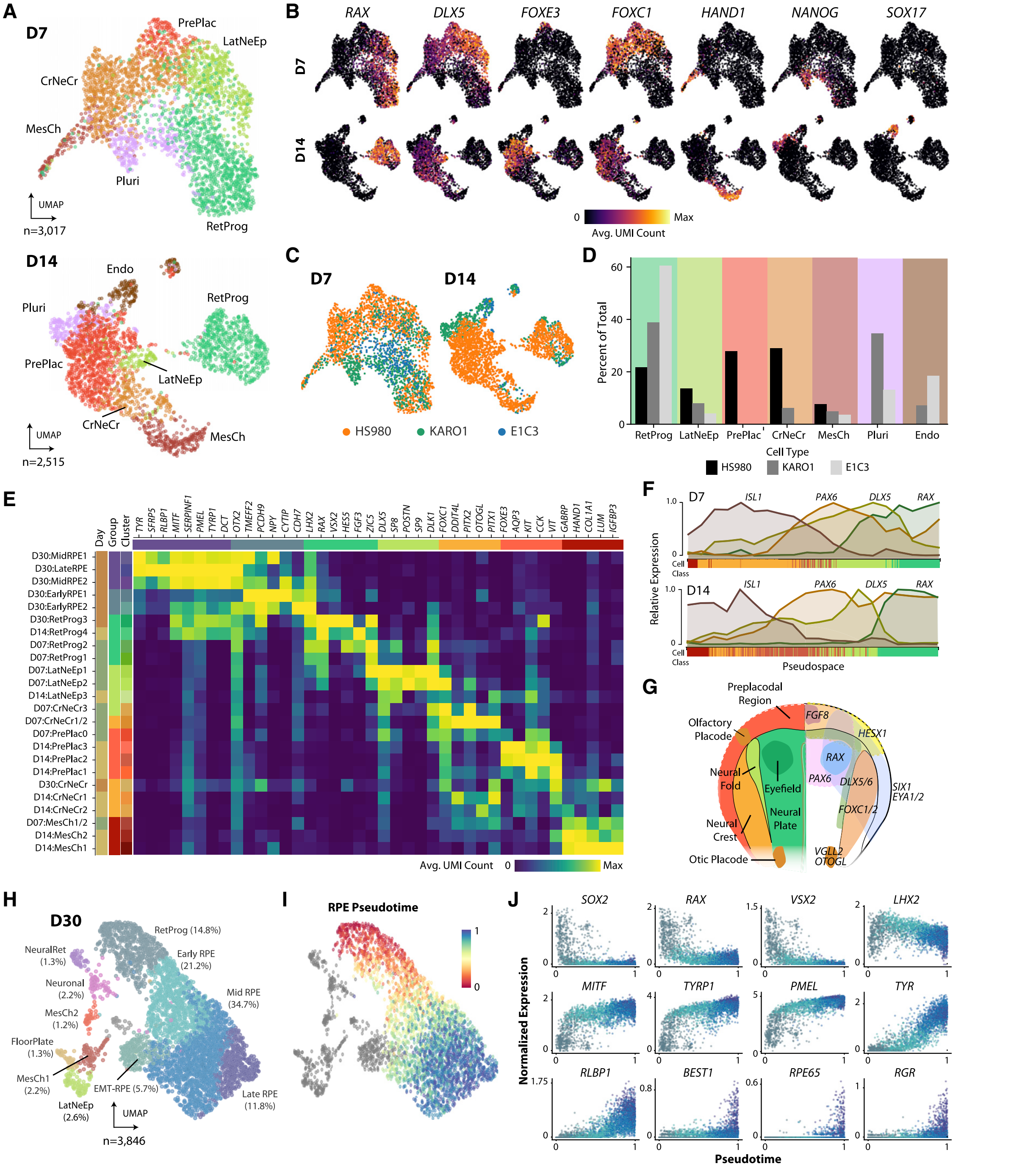

Figure 2. Evaluation of the diverse neuroepithelial cell type derivatives in early hESC-RPE differentiation

(A) Uniform manifold approximation and projection (UMAP) at differentiation day 7 (D7) and D14 in three lines. Cells were grouped into retinal progenitor (RetProg), lateral neural fold-like (LatNeEp), pre-placodal-like (Pre-Plac), cranial neural crest-like (CrNeCr), mesenchyme (MesCh), pluripotent (Pluri), and endoderm-like (Endo) clusters.

(B) UMAPs showing normalized gene expression of marker genes *RAX* (RetProg), *DLX5* (LatNeEp), *FOXE3* (Pre-Plac), *FOXC1* (CrNeCr), *HAND1* (MesCh), *NANOG* (Pluri), and *SOX17* (Endo).

(C) UMAPs in (A) colored by cell line.

(D) Bar graphs showing cell type composition in each line at D7 and D14.

(E) Enriched gene expression heatmap for HS980 cell types.

(legend continued on next page)

Early differentiation recapitulates cellular diversity of the rostral neural tube and optic vesicle

To identify populations during RPE induction, we obtained enriched genes by cluster and cross-referenced the literature, revealing a mixture of intermediate cell states resembling those described in rostral neural tube patterning and eye development (see supplemental experimental procedures). We identified seven groups (pluripotent-like, endodermal-like, lateral neural fold-like, pre-placodal epithelium-like, cranial neural crest-like, mesenchymal, and retinal progenitor) and confirmed these annotations using several literature-supported marker genes (Figures 2A, 2B, and S2A; Table S2; supplemental experimental procedures) (Begbie, 2013; Bosze et al., 2020). This highlighted differences between lines: KARO1 retained pluripotent-like cells at initial time points, HS980 generated more pre-placodal-like cells, and E1C3 initiated an endodermal-like population while also establishing the largest percentage of retinal progenitors (Figures 2C and 2D).

The surprising emergence of different cell types of the anterior ectoderm highlights the inter-relatedness of gene expression programs for the eye, neural crest, and other sensory tissues during early embryonic development. Some secondary clusters matched remarkably well with specific neural tube regions, expressing a combination of markers for eye field (*RAX*, *SIX6*, *LHX2*), telencephalic neural fold (*DLX5*, *DLX6*), lens placodes (*FOXE3*, *PAX6*, *ALDH1A1*), cranial neural crest (*FOXC2*, *VGLL2*, *PITX1*), inner ear placodes (*OTOGL*, *VGLL2*, *CYP26C1*), the anterior neural ridge organizer (*FGF8*, *SP8*, *FOXG1*), and mesenchyme (*GABRP*, *HAND1*, *COL1A1*) (Figure 2E and Table S2; supplemental experimental procedures).

The observation of mesenchyme is interesting because *in vivo* periocular mesenchyme expresses inductive signals that promote RPE fate, a role substituted by Activin A in the protocol. Progenitors (RetProg) detected across differentiation possessed distinct gene expression programs, suggestive of varying degrees of progression toward RPE. RetProg clusters expressed a repertoire of known markers, including *OTX2* and *LHX2*, which are jointly necessary for activation of the transcription factor *MITF*. At D14, these two genes were co-expressed in RetProg clusters alongside MITF-activated genes *PMEL*, *SERPINF1*, *TYRP1*, and *DCT* (Figures 2E and S2B–S2D). Consistent with their classification as progenitors, cells displayed a proliferative signature (S and G_2/M phases) (Figure S2E). Canonical correlation analysis (CCA) further captured a "pseudospatial" axis of variation, with cells transitioning along a mediolateral molecular profile (Figures 2F and 2G; see supplemental experimental procedures).

To factor out time-dependent differences, we integrated D7 and D14 samples onto a shared feature space, where we observed an increase in cells assigned as pre-placodal and a decrease in both inner ear-like cranial neural crest and lateral neuroepithelial cells (Figures S2F and S2G). These analyses revealed that heterogeneity at both stages recapitulates the molecular profile of rostral embryonic territories patterned to specify sensory organs, such as lens, olfactory, and otic placodes (Begbie, 2013) (Figure 2G).

Conversely, between 79% and 96% of cells at D30, depending on the line, were categorized into retinal progenitor or RPE stages. Other observed cell types included lateral neural fold-like, neuronal, mesenchyme-like cluster, neural retina, and floor plate (Figures 2H and S2H). A pseudotemporal trajectory of retinal maturation largely characterized these cells, confirming a loss of progenitor status (*SOX2*, *RAX*, *VSX2*, *LHX2*), followed by an increase in RPE differentiation (*MITF*, *TYRP1*, *PMEL*) and, later, of advanced RPE maturation markers (*TYR*, *RLBP1*, *RPE65*, *BEST1*, *RGR*) (Figures 2I and 2J). Transcription factor network analysis of D30 HS980 cells with SCENIC confirmed the activity of regulons involving gene targets of *SOX2*, *RAX*, *VSX2*, *OTX2*, and *MITF* (Aibar et al., 2017) (Figure S2I). In summary, this data suggest a "divergence-convergence" model, with an initial expansion of cellular diversity, later dampened to favor the promotion of an RPE differentiation program (Figure S2J).

2D hESC-RPE monolayer differentiation is faster and more directed than 3D embryoid body differentiation

The molecular patterning of two-dimensional (2D) cultures during early RPE differentiation hints at an intriguing self-organization process. To clarify how the initial heterogeneity detected in our monolayer differentiation relates to a three-dimensional (3D) protocol that allows cells to organize spatially, we compared it with an embryoid body (EB, HS980 line) differentiation using scRNA-seq (Plaza Reyes et al., 2016).

(F) Plots showing relative expression of neural tube patterning markers in D7 (top) and D14 (bottom) cells across pseudospace in HS980.
(G) Schematic of the patterned anterior neural plate at the neurulation stage. Left: putative location of the cell types corresponding to identified clusters. Right: schematic of genes patterning the rostral embryo.
(H) UMAP of hESC-RPE differentiation D30 in three lines, colored by cell type.
(I) Pseudotime trajectory of all D30 RPE and RetProg cells (82.5% of total at D30).
(J) Scatter plots showing progenitor (*SOX2*, *RAX*, *VSX2*, *LHX2*), early (*MITF*, *TYRP1*, *PMEL*), mid (*TYR*, *RLBP1*), and late (*RPE65*, *BEST1*, *TTR*) gene expression along pseudotime.
See also Figure S2.

Initially, EBs displayed uniform patterning with early progenitor and pluripotency markers, but later showed the emergence of clusters corresponding to fore-, mid-, and hindbrain (Figures 3A, 3B, S3A, and S3B). Interestingly, we detected no traces of the more specific mediolateral-patterning signatures observed in the 2D protocol (Figure 3C). Conversely, midbrain and hindbrain gene expression signatures were not detected in the 2D cultures, and a greater fraction of retinal progenitors were observed in the 2D context (24.7% 2D cells compared to 1.6% 3D cells at D14) (Figures 3D and S3C).

EB cultures also continued to harbor more diversity at D28: while 2D cultures were largely defined by various stages of RPE maturation, in the 3D setting other retinal and brain-related cell types were present, including neural retina, caudal neuroblasts, and glial cells (Figures 3D, 3E, and S3D) (Brodie-Kommit et al., 2021; La Manno et al., 2021). This evidence, in conjunction with the presence of distinct *TBX2*$^{+}$ dorsal optic cup-like and *VAX2*$^{+}$ ventral optic cup-/stalk-like progenitor populations, strongly suggests that a wider set of morphogenetic events are recapitulated during the EB protocol (Bosze et al., 2020).

When 2D-cultured cells were projected onto the EB D28 embedding, the majority of 2D cells mapped to RetProg and RPE clusters rather than non-retinal cell types, illustrating that RPE comprises a much larger fraction of the monolayer cultures (73.4% cells) than EB cultures (10.2% cells) (Figures 3E and 3F). 3D differentiation ultimately produces cells of a broader neural origin whereas 2D differentiation induces rostral identity, further funneled to an RPE fate, thus supporting a divergence-convergence model.

In vitro differentiation and eye development exhibit similarities in cellular composition

We reasoned that embryonic references could validate our model and evaluate how faithfully *in vitro* phenotypes match their *in vivo* counterparts. Thus, we performed scRNA-seq of 12,151 cells from four human embryonic optic vesicles from Carnegie stage 12 (CS12), CS13, CS14, and CS15 (approximately 30, 32, 33, and 36 days/5 weeks post-conception) and two eyes at CS20 (7.5 weeks post-conception). Early stages contained patterned cell types corresponding to optic vesicle and surrounding tissues, including retinal progenitors and RPE (Figures 3G and 3H). Retinal tissues were more clearly differentiated into RPE, neural retinal, and optic stalk subpopulations at CS13 than in RPE-focused progenitors detected *in vitro* (Figure S3E, cf. Figures 2E and S2A; Table S3). CS20 samples captured a more diverse representation of cell types surrounding the eye, including proliferating progenitors, RPE, lens, and intermediate retinal ganglion cells (Figures S3F–S3I).

To evaluate the resemblance of hESC-RPE clusters to embryonic references, we extracted enriched genes from *in vivo* cell types at all stages and used them to compute signature scores for *in vitro* cell types at D7, D14, and D30 (Figure 3I). Scores for RPE clusters *in vitro* were highest using enriched genes from the *in vivo* RPEs, with signatures of later RPE populations *in vitro* scoring higher against later-stage embryonic RPEs. An inverse correspondence was observed between *in vivo* RPE signatures and scores for *in vitro* RetProg populations, relating to their gradual maturity. Signatures for *in vivo* ocular surface ectoderm scored highest on the *in vitro* LatNeEp, CrNeCr, and Pre-Plac clusters, which also had overlapping gene expression patterns for *DLX5*, *PITX1*, and *ISL1*, suggesting that these populations are similar to ectodermal tissue surrounding the optic cup (Figure 3H).

Cell-surface marker NCAM1 defines retinal progenitor cells at D30 of hESC-RPE differentiation

We next aimed to identify cell-surface markers that could distinguish retinal progenitors from RPE cells. We computed a Pearson's correlation coefficient between highly expressed genes at D30 and RPE or neural tube markers (Figures S4A and S4B). Genes with strong anticorrelation to both signatures included transcription factors involved in retina development (*SFRP2*, *CRABP1*, *RAX*, *SIX6*) as well as genes implicated in neural tube (*CPAMD8*, *PKDCC*, *NR2F1*) and lens (*MARCKS*, *DACH1*, *MAB21L1*) development (Figure 4A; see supplemental experimental procedures).

Cell-surface markers *CDH2*, *CPAMD8*, and *NCAM1* were among the most prominent progenitor markers at D30. NCAM1 staining areas coincided with rosette structures lacking pigmentation, and both scRNA-seq and protein staining revealed that NCAM1 was co-expressed with progenitor (VSX2 and RAX) and proliferative (Ki67) markers at D30 (Figures 4B and S4C–S4E).

To functionally examine whether NCAM1-positive cells hold potential to generate RPE cells, we sorted using NCAM1 and CD140b (PDGFRB), an RPE cell marker (Plaza Reyes et al., 2020a) (Figure 4C). Consistently, pigmentation was evident in the CD140b-High population cell pellets, whereas the NCAM1-High population lacked pigmentation (Figure 4D). Transcriptionally, we found that NCAM1-High cells predominantly corresponded to RetProg (64.7%) and EarlyRPE (20.6%), whereas CD140b-High cells were mostly of EarlyRPE (28.0%), MidRPE (42.9%), and LateRPE (17.6%) profiles (Figures 4E–4G, S4F, and S4G).

We then differentiated sorted cells for 30 additional days. Morphological evaluation showed that CD140b-High cells already generated a homogeneous hESC-RPE monolayer at D45, while NCAM1-High cells only yielded a defined RPE morphology at D60 (with cobblestone scores of 6.77e−3 per μm^2 for the CD140b-High population, and 5.29e−3 per μm^2 for the NCAM1-High population at D60)

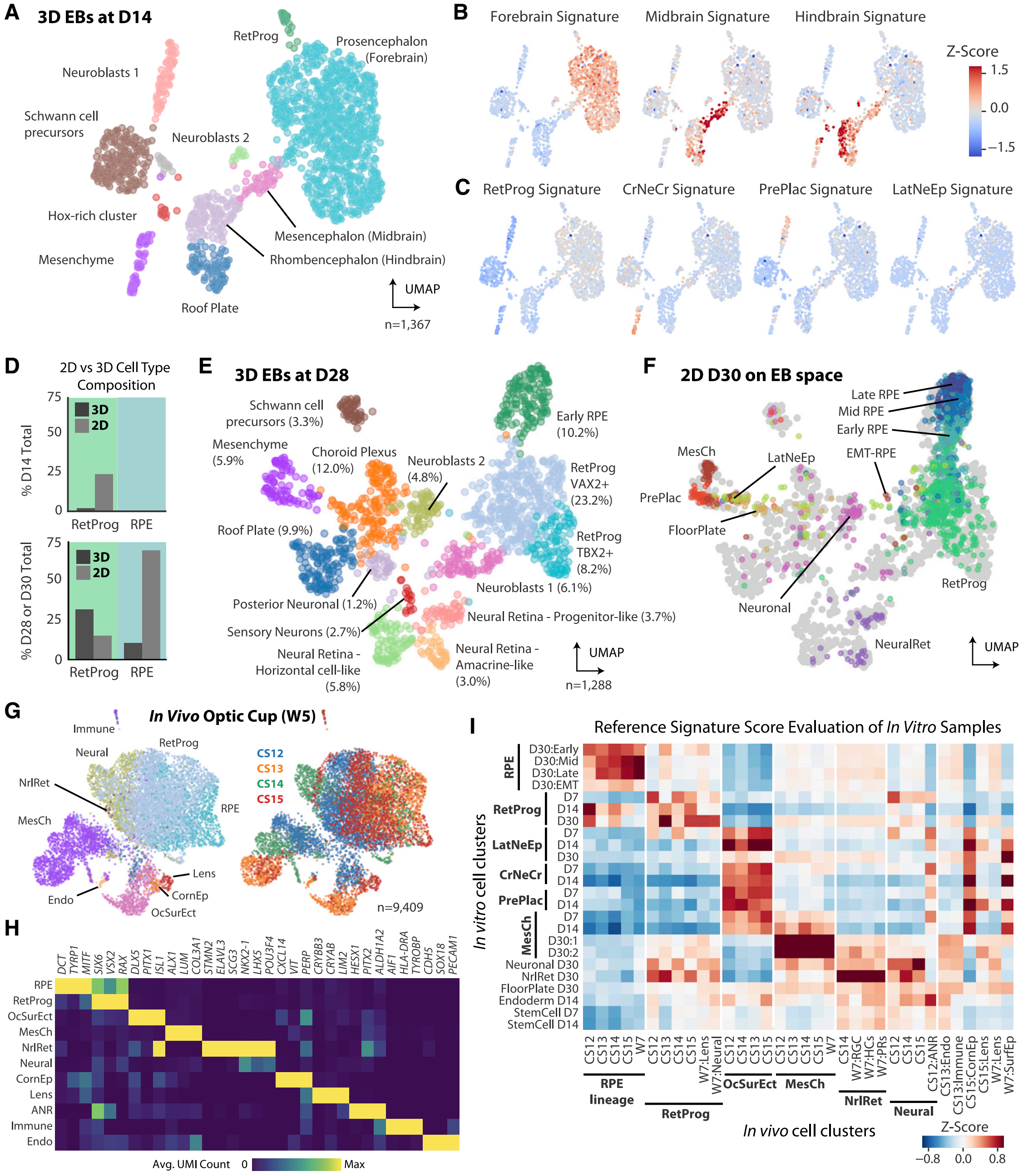

Figure 3. Comparative analysis of RPE induction between hESC-RPE, 3D EB differentiation, and human embryonic eye

(A) UMAP of 3D EB cultures at D14 (HS980 line).

(B and C) UMAPs showing signature scores for brain regions (B) and neural tube cell types (C) visualized on the EB D14 UMAP.

(D) Bar plots comparing cell type compositions in 2D and 3D cultures at D14 (top) and D28/30 (bottom).

(E) UMAP of 3D EB cultures at D28 colored by cell type.

(F) Projection of 2D D30 cells from all three cell lines onto the UMAP from (E) using pairwise correlation distances, colored by annotated cell type (see supplemental experimental procedures, cf. Figure 2H). Cells in gray are those from (E).

(G) UMAP of human embryonic optic cup cells at Carnegie stages 12, 13, 14, and 15 (week 5, W5), colored by cell type (left) or stage (right).

(H) Heatmap of enriched gene expression by cell type across all samples in (G).

(I) Heatmap showing signature scores of *in vitro* cell clusters at D7, D14, and D30 illustrating the correspondence to *in vivo* clusters from (G). Signature scores were obtained using the top 30 genes of the respective *in vivo* reference population.

See also Figure S3.

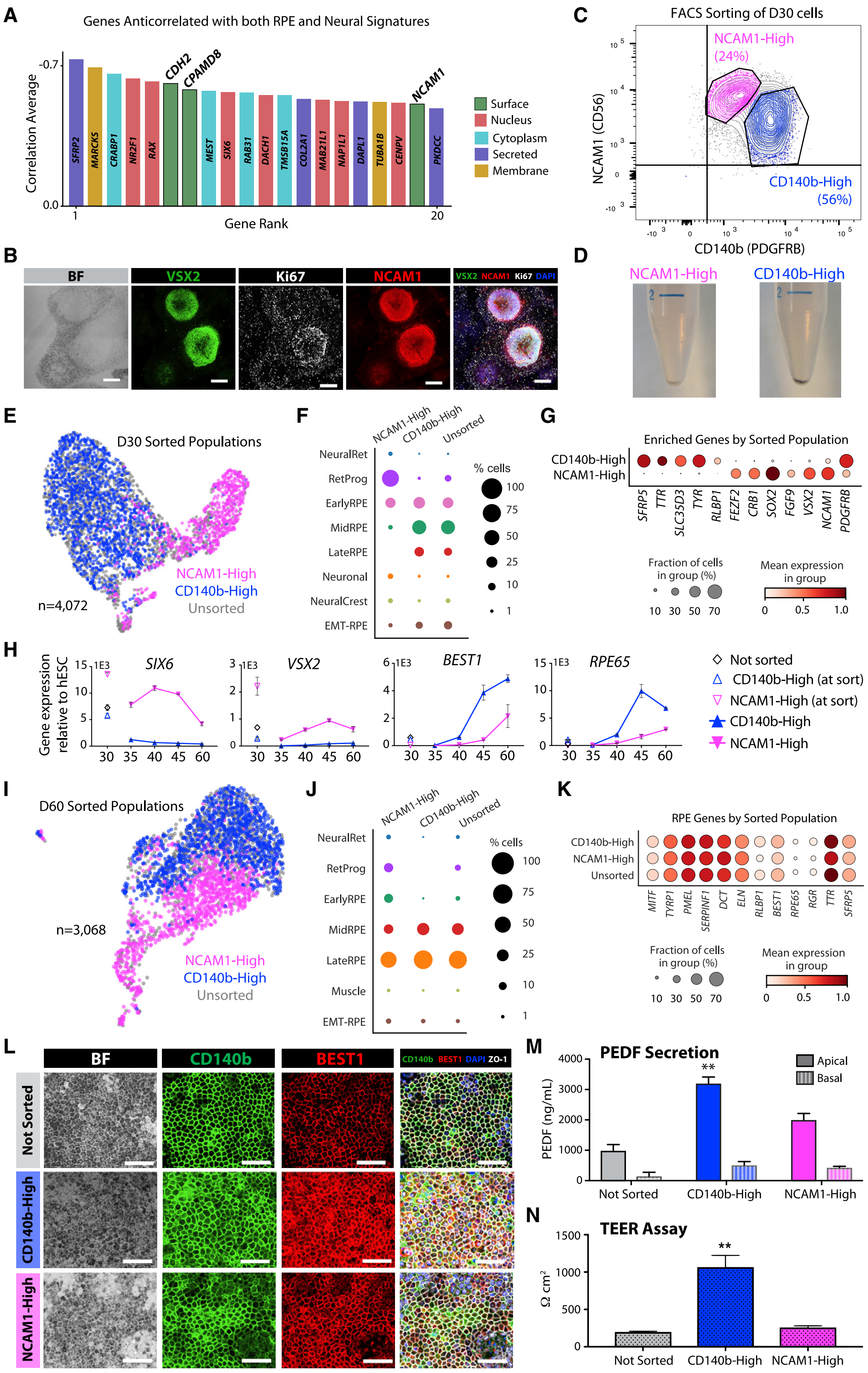

(legend on next page)

(Figure S4H, cf. Figure S1A). NCAM1-High cells were positive for the proliferation marker Ki67 and retinal progenitor transcription factor VSX2 (Figures S4I and S4J). These patterns were further confirmed by qRT-PCR (Figures 4H and S4K).

Establishment of an RPE phenotype at D60 by both NCAM1-High and CD140b-High populations was confirmed by scRNA-seq and immunofluorescence (Figures 4I–4L, S4L, and -S4M). Functionally, we assessed pigment epithelium-derived factor (PEDF) secretion and transepithelial resistance (TEER) upon protocol completion (D60), finding that CD140b-High-derived cells secreted significantly higher apical levels of PEDF than the unsorted and NCAM1-High-derived cells (Figure 4M). TEER levels displayed by CD140b-High-derived cells were higher compared to unsorted and NCAM1-High populations (Figure 4N). These results show that NCAM1 captures an immature progenitor population with potential to mature into functional RPE cells.

NCAM1-High cells can differentiate into alternative retinal cell types

To further evaluate the differentiation potential of D30 NCAM1-High cells, we plated sorted cells in neuroretinal promoting conditions for 40 additional days (Shao et al., 2017) (HS980 line, Figure 5A). NCAM1-High cells gave rise to a heterogeneous culture with a significant portion of cells displaying a distinct non-RPE cell body morphology, unlike CD140b-High cells under the same conditions (Figure 5B). Transcriptional profiling showed that only 12% of the analyzed cells were RPE, suggesting that NCAM1-High cells at D30 represent an uncommitted progenitor with potential beyond RPE whereas CD140b-High captures lineage-committed RPE cells (Figure 5C).

To systematically compare NCAM1-High-derived cells to a developmental reference, we performed CCA with week 7.5/CS20 embryonic eyes. The shared low-dimensional space emphasized similarities between corresponding clusters, including RPE, progenitor, lens, surface epithelial, and neuronal populations (Figures 5D–5H). Moreover, there was an overlap between week 7.5 retinal ganglion and NCAM1-High-derived neurons (Figures 5D and 5E, cf. Figure S3). To compare gene expression dynamics, we computed RNA velocity on each neuronal population, revealing progression toward a more mature state (La Manno et al., 2018) (Figure 5I). Pseudotemporal gene expression confirmed a common profile of expression waves, with gradual downregulation of proliferation markers (*TOP2A*, *MKI67*) followed by upregulation of a neuronal differentiation program (*STMN2*, *TUBB2A*, *DCX*) (Figure 5J). However, critical markers of retinal ganglion development, such as transcription factor *ATOH7* and its downstream targets *POU4F2* and *ISL1*, were only expressed in the week 7.5/CS20 cells (Gao et al., 2014) (Figure 5K). Other neuronal markers (*EOMES*, *NEUROD2*, *SLA*) were unique to NCAM1-High-derived neurons, implying that NCAM1-High-derived cells are another type of telencephalic neuron (Figure 5L). NCAM1-High cells are thus either a mixed pool of retinal and neuroepithelial progenitors capable of forming both cell types and other related retinal lineages or cells with the capacity to establish all of these lineages.

Figure 4. Characterization of the NCAM1-High sorted D30 hESC-RPE population

(A) Bar graph of top genes from anticorrelation analysis at HS980 D30. Genes with a mean normalized expression <0.5 were excluded.

(B) Brightfield and immunofluorescence stainings of D30 cells showing co-expression of VSX2, NCAM1, and Ki67 markers. Scale bars, 200 μm.

(C) Representative FACS plot of NCAM1-CD140b sorting to distinguish distinct populations at D30. Negative gates were set based on fluorescence minus one (FMO) and hESC control samples.

(D) Post-sort pellets of CD140b-High and NCAM1-High cells.

(E) UMAP of NCAM1-High (pink), CD140b-High (blue), and unsorted (gray) D30 cells after CCA integration.

(F) Dot plot illustrating the proportion of cells corresponding to each identified cell type in scRNA-seq samples from (E).

(G) Dot plot of selected progenitor (*FEZF2*, *CRB1*, *SOX2*, *FGF9*, *VSX2*) and RPE (*SFRP5*, *TTR*, *SLC35D3*, *TYR*, *RLBP1*) genes enriched in the sorted samples.

(H) Graphs showing qRT-PCR of retinal progenitor (*SIX6*, *VSX2*) and RPE (*BEST1*, *RPE65*) marker genes in populations from (E) at the moment of sort and at post-sort D30, D35, D40, D45, and D60.

(I) UMAP of NCAM1-High (pink), CD140b-High (blue), and unsorted (gray) D60 cells.

(J) Dot plot illustrating the proportion of cells corresponding to each identified cell type in scRNA-seq samples from (I).

(K) Dot plots of early (*MITF*, *TYRP1*, *PMEL*, *SERPINF1*, *DCT*, *ELN*) and late (*RLBP1*, *BEST1*, *RPE65*, *RGR*, *TTR*, *SFRP5*) RPE genes in the LateRPE cell clusters from each sorted sample.

(L) Brightfield and immunofluorescence stainings of unsorted, CD140b-High, and NCAM1-High populations 30 days after sorting (D60) showing co-expression of CD140b, BEST1, and ZO-1 markers. Scale bars, 100 μm.

(M and N) Bar graphs showing PEDF secretion (M) and TEER measurements (N) of the unsorted, CD140b-High, and NCAM1-High populations at D60. **p < 0.0001 compared to Not Sorted and NCAM1-High.

In (H), (M), and (N), error bars represent mean ± SEM from three independent experiments. See also Figure S3.

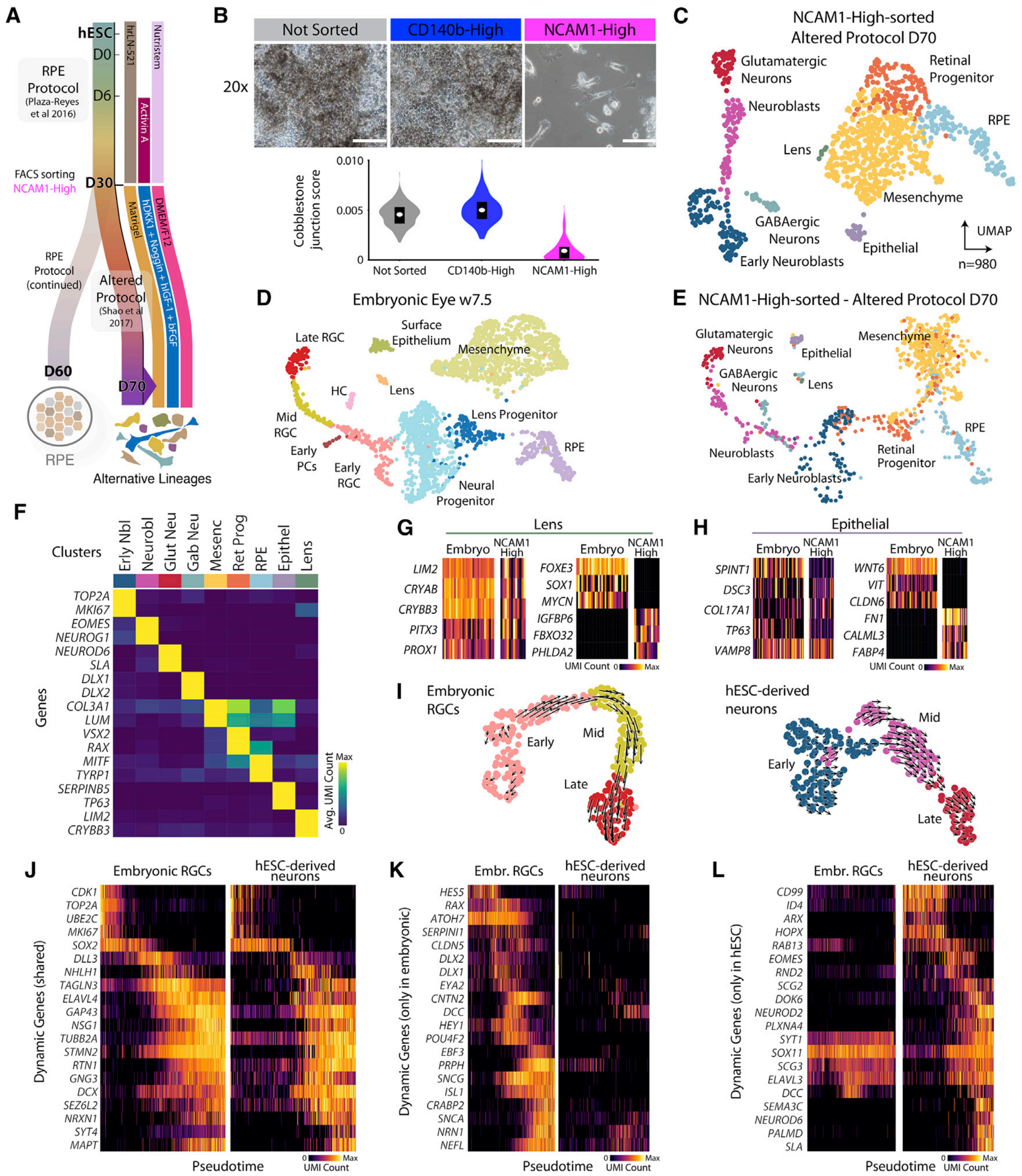

Figure 5. Neuroretinal progenitor differentiation of NCAM1-High-sorted hESC-RPE D30 cells

(A) Schematic of the neuroretinal progenitor (altered) differentiation protocol (HS980 line). D30 NCAM1-High-sorted cells were sorted and replated on Matrigel containing DMEM/F12, hDKK1, Noggin, hIGF-1, and bFGF until scRNA-seq at D70.

(B) Brightfield images and cobblestone junction scores of sorted and unsorted populations at D70. Scale bars, 100 μm.

(C) UMAP of NCAM1-High sorted cells at D70.

(D and E) CCA integration of scRNA-seq data from embryonic week 7.5 eye (D) and NCAM1-High-sorted cells subjected to the altered protocol (E).

(F) Heatmap of enriched gene expression for cell types in (C).

(G and H) Gene expression heatmaps of lens (G) and epithelial (H) cells identified in the reference and *in vitro*. Shared and differentially expressed genes are shown on the left and right plots, respectively.

(I) RNA velocity of embryonic retinal ganglion cells (left) and hESC-derived neurons (right).

(J–L) Heatmaps showing gene expression analysis of embryonic and hESC-derived neurons along their respective pseudotimes.

RGC, retinal ganglion cell; PC, photoreceptor cell; HC, horizontal cell.

Late differentiation is characterized by the selection and maturation of RPE populations

Unlike initial stages, scRNA-seq of three time points after replating revealed that most cells were of an RPE state: the proportion of LateRPE cells was 17.1% at D38, 55.7% at D45, and 77.7% at D60. By D60, approximately 98.2% of cells were of some RPE identity, with the remaining fraction consisting of retinal progenitors. At intermediate D38 and D45, small fractions of non-retinal mesenchyme and smooth muscle contaminants were detected. However, these populations were no longer present in culture at D60 (Figures 6A–6C and Table S5). We also detected a distinct cluster of lingering pluripotent cells in the HS980 D38 sample (0.9% of cells) expressing pluripotency markers *SOX2*, *LIN28A*, *SALL4*, and *GPC3* (Figure 6A). As such cells must be eliminated from the final cell product, we extended our analysis to include eight independent D60 samples containing 63,370 cells across all three lines. Encouragingly, not a single cell with a pluripotent signature was detected in the D60 samples (Figures S5A, S5B, S1H, and S1I).

D38 cells also displayed an increased heterogeneity and on average showed a less distinct RPE cobblestone morphology than D30 (Figures 1C and S1A). Furthermore, from D30 a cell population co-expressed *MITF* and markers associated with the epithelial-to-mesenchymal (EMT) transition process, particularly *ACTA2*. The dissociation of RPE cells at D30 likely induced a mesenchymal-like morphology of the RPE (Figure 1B). This observation led to characterization of two early RPE clusters from D30 onward, one $MITF^{+}ACTA2^{-}$ (EarlyRPE) and one $MITF^{+}ACTA2^{+}$ (EMT-RPE) (Figure S5C). EMT-RPE expressed some but not all RPE markers while co-expressing EMT genes. The fraction of EMT-RPE increased during replating from D30 to D38, followed by a steady decrease to low levels (0.8% of cells) by D60 (HS980). Moreover, the representation of RPE from later time points along a phenotype variation axis confirmed the presence of shared EMT and RPE differentiation properties (Figures S5C–S5F).

Nonetheless, after D30 we observed the persistence of RPE and loss of other cell types; pseudotime inference and RNA velocity showed a trajectory of less mature populations in gene expression space toward the most mature RPE (HS980) (Figure 6D). Phase portrait analysis comparing the steady-state expectations for spliced and unspliced RNA levels further confirmed the upregulation of *RPE65* and *BEST1* as well as the downregulation of progenitor marker *PAX6* (Figure 6E; see supplemental experimental procedures).

Replating affects cell population composition and promotes a purer and more mature cell product

We previously showed that replating D30 monolayer cultures facilitates the expansion of final cell numbers (Plaza Reyes et al., 2020a), but we had not explored how replating affects maturation and purity. We therefore repeated our differentiation protocol without the replating step and performed scRNA-seq, revealing significant contamination with cells resembling neural retina, neuronal, lens, mesenchyme, neural crest, and mesoendoderm (Figures S5G and S5H). The presence of contaminant types was confirmed by flow cytometry for lack of the RPE marker CD140b (Figure S5I). Non-replated cultures at D60 maintained more RetProg cells and fewer LateRPE cells (Figures S5H and 6C). We determined that replating selects against retinal progenitors and arrests the expansion of alternative lineages. This was paralleled by increased RPE cobblestone morphology in D60 replated cultures compared to non-replated counterparts (Figure S5J).

To assess overall progression of hESC-RPE and assign cells to developmental stages, we constructed an ordinal classifier using transcriptomes of 783 embryonic eye cells from week 5 to week 24 and 127 adult RPE cells (Hu et al., 2019; Voigt et al., 2019) (Figures S6Aand S6B). As proof of principle, we applied the classifier to CS13 and CS20 embryonic references and to an independent set of 49 adult RPEs, confirming their appropriate assignment (Quake and Sapiens Consortium, 2021) (Figures S6C). Evaluation of the developmental maturity confirmed that replating leads to a more mature output (Figure S5K). Furthermore, classification of the maturation status for all *in vitro* retinal progenitor and RPE cells confirmed a gradual progression throughout differentiation corresponding to embryonic RPE development; the classifier also assigned a consistent maturation level among all D60 lines and replicates (Figures 6F, 6G, and S6D).

Lastly, we compared the maturation of RPE cells from our D60 monolayer protocol to RPE cells generated through 3D EB differentiation and to those from other protocols with a longer differentiation. D60 RPE cells in either 3D or 2D differentiation showed similar maturation statuses (Figure S6H). In addition, reanalysis and classification of scRNA-seq data in which differentiation was performed for 95 or 432 days using another 2D monolayer protocol showed that our D60 cells are similar in maturation status to the D95 cells, but that further maturation can be achieved through extensive *in vitro* culturing (Lidgerwood et al., 2021) (Figures S6E–S6H). Interestingly, while 95- and 432-day time points contained highly mature RPE, both samples also included fractions of retinal progenitors and EMT-RPE, as also observed in D60 samples (Figures S6G and S6H).

Subretinal transplantation of hESC-RPE facilitates a more advanced RPE state

Given the therapeutic potential of hESC-derived RPE, we next wanted to investigate the transcriptional profiles of cells following *in vivo* transplantation to assess whether cells continued to mature and ensure that alternative lineages

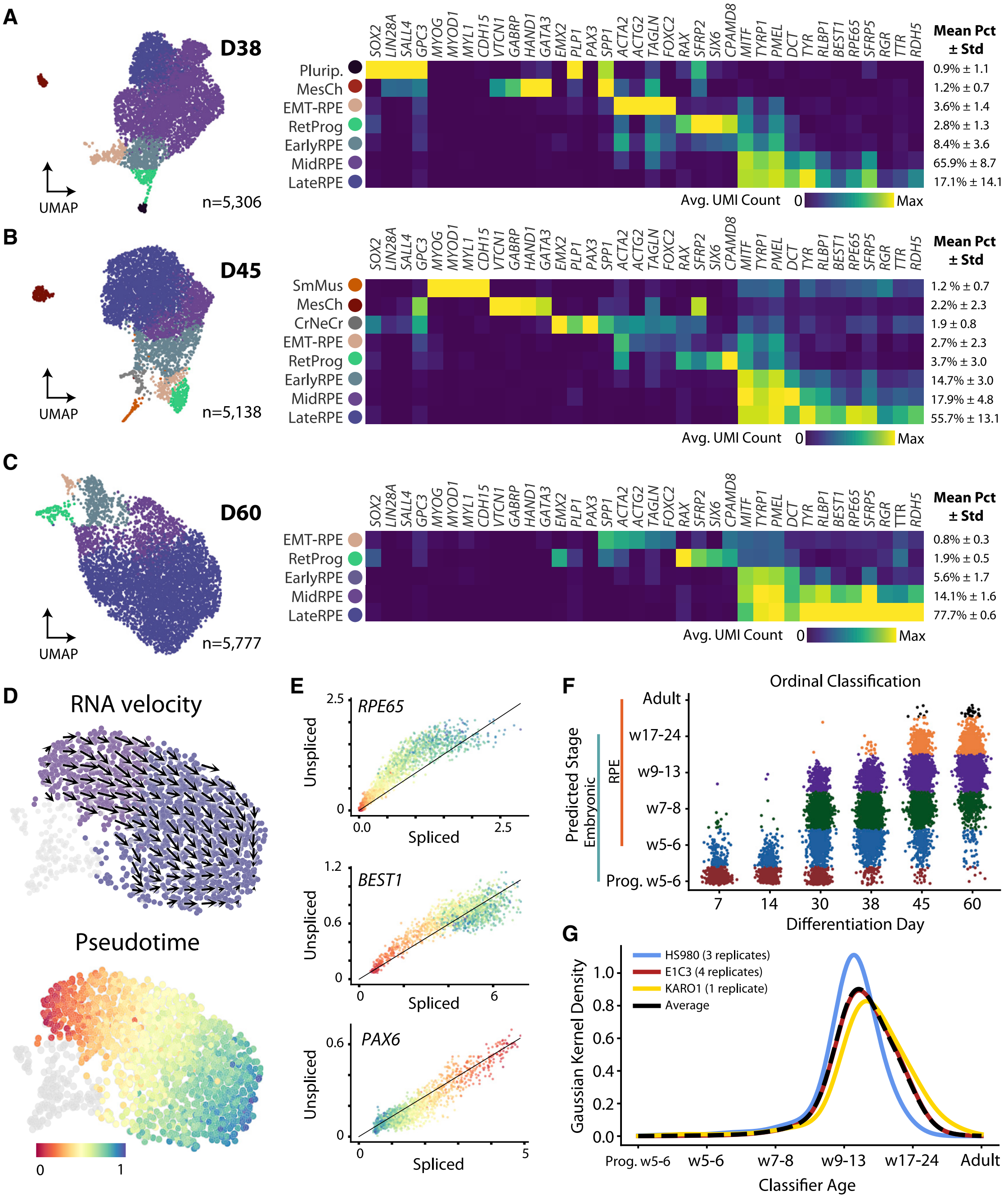

Figure 6. Late hESC-RPE differentiation profiling

(A–C) UMAPs and enriched gene expression heatmaps of hESC-RPE scRNA-seq data at D38 (A), D45 (B), and D60 (C) in all three lines.
(D) RNA velocity and pseudotime analysis of HS980 RPE at D60.
(E) Phase portraits of upregulated RPE marker genes *RPE65* and *BEST1* as well as a downregulated progenitor marker *PAX6*. The diagonal line represents the estimated steady state of gene expression, with cells above the steady state experiencing gene upregulation and those below gene downregulation.
(F) Plot showing ordinal classification of 20,682 single hESC-derived retinal progenitor and RPE cells at six differentiation time points along embryonic stages.
(G) Graph representing classification distribution for seven hESC-RPE differentiation D60 biological replicates (HS980: 3,655 cells; E1C3: 61,479 cells; KARO1: 1,236 cells).
See also Figure S6.

remain absent. D60 hESC-RPE cells (HS908 line) were therefore transplanted into the subretinal space of two albino rabbits, a preclinical large-eyed animal model with an assessed high degree of transcriptional similarity (Figure S7 and Table S6). Four weeks following transplantation, infrared and spectral domain optical coherence tomography (SD-OCT) imaging showed pigmented patches of hyperreflective RPE layer in the albino rabbit retina (Figure 7A). Histology and immunofluorescence analysis of 227 human-NuMA$^+$ single cells found that 99.56% were either pigmented or expressed the RPE marker BEST1, thus corroborating the successful integration of injected hESC-RPE cells in a polarized and matured RPE monolayer (Figures 7B, S6I, and S6J). The contiguous injected retina of two rabbits was then processed for scRNA-seq, yielding 65 human hESC-derived cell profiles that all exhibited a high expression of mature RPE markers. Crucially, markers of retinal progenitors, photoreceptors, pluripotent hESCs, and EMT-RPE were benchmarked against our references and undetected *in vivo* (Figure 7C).

Gene expression correlation analysis of grafted cells to embryonic references and *in vitro* clusters from D30 and D60 confirmed similar patterns overall, yet one *in vitro* cluster (D60: LateRPE) and the *in vivo* transplanted RPE were the most similar to the adult RPE reference (Figure 7D, cf. Figures 3, S3, and S6). Differential expression analysis confirmed an expression pattern closer to adult RPE cells after *in vivo* implantation, particularly for visual cycle components (Figure 7E). Interestingly, ordinal classification of the retrieved 65 post-transplantation human cells showed that the gradual progression of maturity *in vitro* continued further in the *in vivo* environment. Transplanted hESC-RPE were assigned into the late embryonic weeks 17–24 (26%) and adult RPE ordinal classes (25%) more than any *in vitro* time point, whereas D60 cells were predominantly assigned to embryonic weeks 9–13 (93%) (Figures 7F and 7G).

DISCUSSION

By molecular profiling an hESC-RPE differentiation protocol established for clinical translation, we demonstrate that the described culture conditions successfully specify RPE lineage, selection, and maturation over 60 days through a sequence of gene expression waves consistent with embryological studies (Fuhrmann et al., 2014; Hu et al., 2019). At early stages, we found cell pool heterogeneity incompatible with the induction of a single lineage and, instead, evidence of widespread initial cellular diversity. Similar heterogeneity expansion was observed in studies of endoderm and endothelial tissue derivation, but meta-analysis of several differentiation protocols is needed to understand whether the observed event is a widespread phenomenon (Cuomo et al., 2020). Our findings suggest a divergence-convergence model: heterogeneity expansion at early time points (influenced by cell-line-specific properties), followed by selection of RPE lineage (driven by replating at D30), and convergence onto a homogeneous and highly pure cellular product.

Each cell line manifested different biases to this initial diversification: E1C3 developed endoderm-like cells, HS980 produced populations reminiscent of different rostral neural tissues, and a fraction of KARO1 displayed signatures of lingering pluripotent cells at the earliest time points. Particularly interesting is the finding of expression profiles resembling patterned regions surrounding the optic field: the pre-placodal epithelium, neural fold, and neural crest. This axis of embryonic patterning is induced by organizer cells of the floor plate and anterior neural ridge, which promote specification of different anterior neural tube territories, including the optic vesicle (Begbie, 2013; Streit, 2007). These findings hint at an intriguing self-organization process occurring in 2D culture, despite the lack of spatially directed cues or 3D structure.

Contrasting with embryonic and adult references, our data highlight that an adult RPE pattern of expression is not yet reached in D60 cells. While a more mature stage can be achieved after 1 year of further culturing, even such long-term RPE cultures still contain persisting retinal progenitor and EMT-RPE populations (Lidgerwood et al., 2021). Further studies are warranted to elucidate the function of these populations to understand whether they may represent a normal part of RPE physiology and how they may impact cell therapy products.

We demonstrated that NCAM1-High cells are not RPE-fate restricted and, upon altered culture conditions, can give rise to additional cell types including anterior neurons, mesenchyme, and lens epithelium. This potency is particularly relevant, as the identification and isolation of less mature progenitors with an increased plasticity is of importance to efforts aimed at replacement of other retinal cell types affected by advanced AMD (Bhatia et al., 2010; Marquardt et al., 2001). Further evaluation of the NCAM1-High potency as a response to different and more specific culture conditions, and in other *in vivo* models lacking certain retinal cell types, constitute promising avenues for future investigation.

Considering that the final cell product may contain 2% of such retinal progenitors, it should be averted in the future despite being unlikely to pose a safety risk. However, we did not detect any non-RPE cell types from our histological (227 cells) or transcriptional (65 cells) analysis following cell transplantation, suggesting that the progenitor pool has not expanded or generated alternative lineages. Additionally, our analysis highlights the importance of ensuring that the final cell product does not contain lingering PSCs at a single-cell level, especially as we detected unexpected

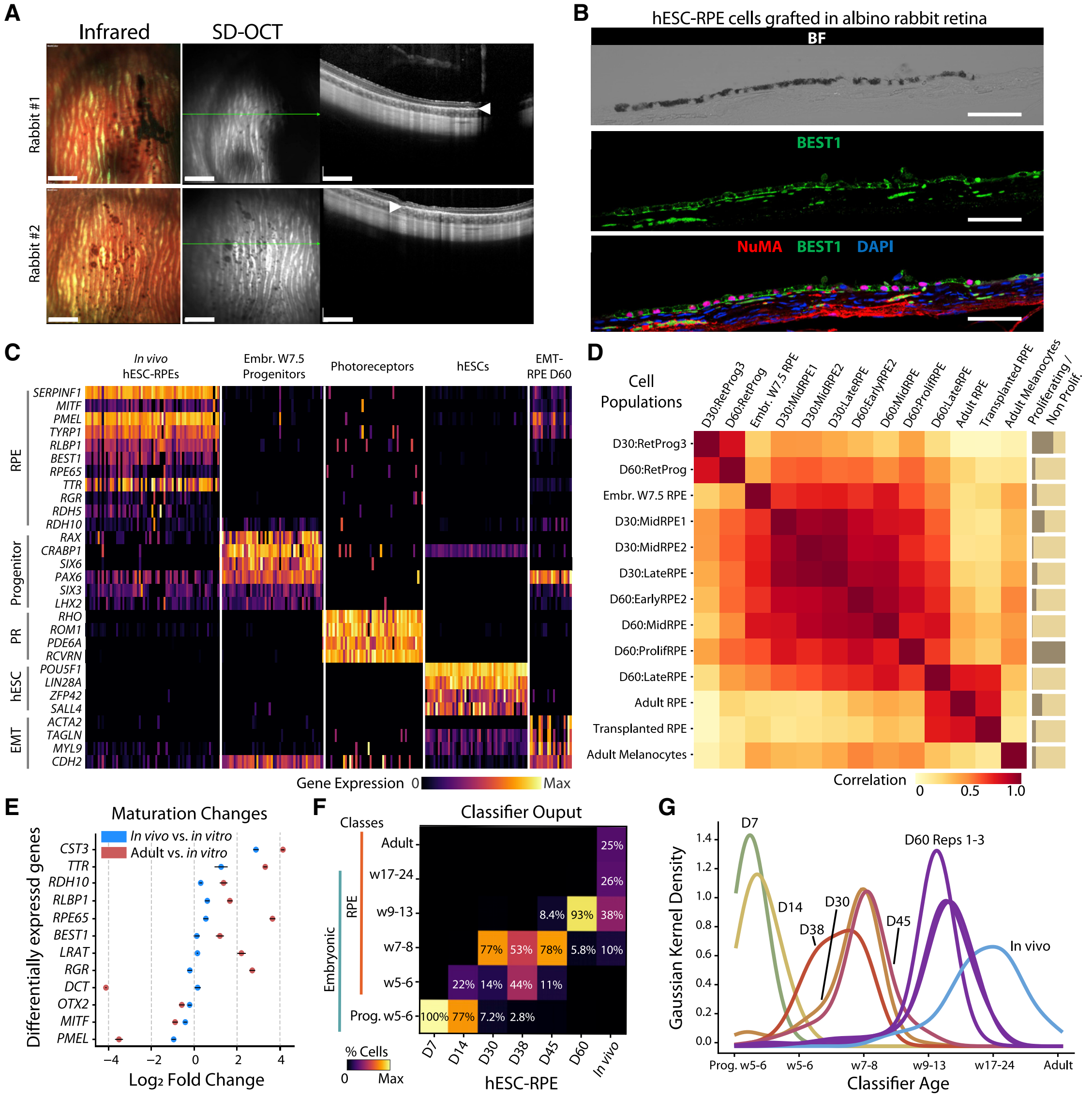

Figure 7. Phenotyping of hESC-RPE transplanted in the albino rabbit subretinal space

(A) Infrared and SD-OCT images of injected hESC-RPE cells (HS980 line) in the subretinal space of albino rabbits. Green lines indicate the SD-OCT scan plane. White arrows indicate the hyper-reflective RPE layer. Scale bars, 1 mm.

(B) Brightfield and immunofluorescent staining for human marker NuMA and BEST1 30 days after injection. Scale bars, 50 μm.

(C) Gene expression heatmap comparing 65 single hESC-RPE cells 30 days after transplantation to embryonic week 7.5 retinal progenitors, adult photoreceptors, undifferentiated hESCs, and D60 EMT-RPE.

(D) Pearson's correlation matrix between gene expression profiles of HS980 hESC-RPEs at D30 and D60, post-transplantation (*in vivo*) RPE, adult RPE and melanocytes, and embryonic RPE.

(E) Dot plot graph showing $\log_2$ fold change of RPE markers between HS980 hESC-RPE D60 cells, *in vivo* RPE, and adult RPE. Error bars represent mean ± SEM from all cells at each time point.

(F) Ordinal classification summary matrix showing the percentage of HS980 retinal cells from *in vitro* and *in vivo* time points predicted to correspond to each RPE developmental time point (embryonic weeks 5–24, adult).

(G) Graph showing classification distribution for hESC-derived progenitor and RPE cells *in vitro* and *in vivo*.

See also Figure S7.

pluripotent signatures as late as D38. Importantly, our focused analysis showed that at D60, none of the eight samples from the three cell lines (63,370 cells) contained cells with a pluripotent profile.

The behavior of grafted cells *in vivo* is a topic discussed extensively by the community, with maintenance of the proliferative potential and dedifferentiation generally considered the two processes of major concern (Wang et al., 2020; Zarbin et al., 2019). Our analysis identified neither specific signs of dedifferentiation nor the presence of a non-RPE molecular profile. Instead, we detected a distinct shift in the RPE maturation toward a more adult phenotype. The induction mechanism of the observed *in vivo* maturation remains unclear, albeit the increased expression of visual cycle genes suggests that grafted hESC-RPE cells support neighboring photoreceptors functionally.

Overall, our findings provide a high-resolution perspective on hPSC differentiation and a necessary detailed analysis of a stem cell-based product intended for successful and safe human therapeutic strategies. Ultimately, this study will guide future efforts focused on the differentiation of retinal cells, leading to a deeper understanding of mechanisms of retinal disease and applications in regenerative medicine.

EXPERIMENTAL PROCEDURES

hESC cell culture and hESC-RPE differentiation

hESC lines HS980 and KARO1 were established and cultured in 5% CO_2/5% O_2 on rhLN-521 (10 μg/mL) and passaged as described previously (Rodin et al., 2014). E1C3 (NN GMP0050E1C3) cultured on iMatrix-511 (0.25 μg/cm^2, Nippi, T303) was provided as a research cell bank of the clinical GMP cell line by NovoNordisk (UCSF IRB: 1518222, for RPE differentiation Projekt-ID: H-18016740, Anmeldelsesnr.: 73105).

For differentiation (Plaza Reyes et al., 2020a, 2020b), cells were plated at a density of 2.4×10^4 cells/cm^2 on 20 μg/mL hrLN-521 or iMatrix-coated dishes using NutriStem hPSC XF medium and Rho-kinase inhibitor (10 μM) during the first 24 h. Medium was then replaced with NutriStem hPSC XF without basic fibroblast growth factor (bFGF) and transforming growth factor β (differentiation medium) in 5% CO_2/21% O_2, and from day 6 after plating, 100 ng/mL of Activin A was added to the medium for a total of 30 days. Day-30 monolayers were replated using TrypLE Select (10 min, 37°C) and passed through a 40-μm strainer. Cells were seeded on hrLN-521-coated dishes (20 μg/mL) at 6.8×10^4 cells/cm^2, and fed three times a week for 30 subsequent days with differentiation medium without Activin A.

Sample processing for single-cell RNA sequencing

For cells, specific stage hESC-RPE cells were trypsinized with TrypLE (10 min, 37°C, 5% CO_2) and resuspended to 1,000 cells/μL in 0.04% BSA in PBS prior to scRNA-seq.

For tissues, two human 32-h postmortem eyes from the same donor were collected; the retinas were dissected out and cut into several small pieces mixed together in 500 μL of digestion buffer (see supplemental experimental procedures). Two pooled embryonic eyes at Carnegie stages 12, 13, 14, and 15 (5 weeks post-conception) and two embryonic eyes from the same donor (7.5 weeks post-conception) were collected. Optic cups were dissected out and chopped in several small pieces to facilitate dissociation in 500 μL of digestion buffer. Two rabbit eyes (from different animals) with 30-day integrated hESC-RPE were enucleated and neuroretina, choroid, and RPE layers were dissected out and mixed together in 500 μL of digestion buffer. After digestion (37°C, 25 min on a 300 × *g* rotator, resuspended every 5 min), samples were filtered using a 30-μm strainer followed by a Dead Cell Removal kit. At this stage, one of the rabbit eye cell samples was stained with mouse anti-human HLA-ABC-FITC; HLA-ABC-positive cells were sorted by fluorescence-activated cell sorting (FACS), collected, and resuspended to 1,000 cells/μL in 1% BSA in PBS. The rest of the samples were also resuspended to 1,000 cells/μL in 1% BSA in PBS prior to scRNA-seq.

Single-cell RNA sequencing analysis

Cells were either transported at 4°C to the Eukaryotic Single Cell Genomics Facility (ESCG; SciLifeLab, Stockholm, Sweden) or used in-house to prepare cDNA libraries for scRNA-seq. The 10x Genomics Single Cell 3′ Reagent Dual Index Kit v2 and v3.1 (10x Genomics, CG000315) was used, sometimes with an additional Cell Multiplexing Oligo Labeling step (10x Genomics, CG000391), followed by protocol CB000388 and sequencing on a NovaSeq 6000 (ESCG) or Illumina Nextseq 2000 (in-house). Cell Ranger 3.1.0 was used to convert base call files to FASTQ format, map sequencing reads to the human GRCh38 reference transcriptome, and generate feature-barcode matrices. For the E1C3 cell line sequenced at NovoNordisk, CellRanger 3.0.2 was used. Quality control, normalization, dimensionality reduction, and visualization were performed using the *scanpy* and *velocyto* modules (La Manno et al., 2018; Wolf et al., 2018). For samples on which RNA velocity was performed, the *velocyto run10x* command was used on CellRanger sorted BAM files to produce loom files containing spliced and unspliced counts. Cell filtering, dimensionality reduction, and visualization criteria are provided for each individual sample in supplemental experimental procedures and Table S1.

Subretinal transplantation and *in vivo* imaging

Dissociated hESC-RPEs were injected in sterile PBS (50 μL; 50,000 cells) subretinally using a transvitreal pars plana technique in New Zealand white albino rabbits (Bartuma et al., 2015; Petrus-Reurer et al., 2017, 2018). SD-OCT and confocal scanning laser ophthalmoscopy was performed to obtain horizontal cross-sectional B-scans and *en face* fundus *in vivo* images, respectively.

Data and code availability

FASTQ files, processed feature-barcode count matrices, annotated h5ad/loom files, and other metadata are available on the Gene Expression Omnibus (GEO: GSE164092). Jupyter notebooks for the single-cell analyses are shared at https://github.com/lamanno-epfl/rpe_differentiation_profiling_code. Datasets are available for interactive visualization and analysis at https://asap.epfl.ch/ under public keys ASAP 75–90 (David et al., 2020).

SUPPLEMENTAL INFORMATION

Supplemental information can be found online at https://doi.org/10.1016/j.stemcr.2022.05.005.

AUTHOR CONTRIBUTIONS

S.P.-R., A.R.L., F.L., and G.L.M. conceived the study; F.L., G.L.M., and J.C.V. supervised the work; S.P.-R., L.B.-V., I.K., M.W., H.A., E.S., A.B., A.W., Y.S., P.E., A. Kriegstein, and A. Kvanta performed experiments; I.D. and B.P. helped with the cell sorting; H.B. and M.A. contributed to the animal work; A.R.L., E.J., H.W., and G.L.M. performed scRNA-seq analysis; S.P.-R., A.R.L., A.K., G.L.M., and F.L. planned experiments, analyzed data, and wrote the manuscript.

CONFLICTS OF INTEREST

S.P.-R., and F.L. are the inventors of a patent ("Methods and compositions for producing retinal pigment epithelium cells," filed 19.06.2019, PCT/EP2019/066285).

ACKNOWLEDGMENTS

We thank Ernest Arenas, Pierre Fabre, Igor Adameyko, Pierre Gönczy, Felix Naef, and Bart Deplancke for helpful feedback on the manuscript. We also thank the EPFL Histology Core Facility team, particularly Jessica Dessimoz, Gian-Filippo Mancini, and Nathalie Müller, for their assistance with immunostaining. The work was supported by grants from the Swedish Research Council, Ragnar Söderberg Foundation, Ming Wai Lau Center for Reparative Medicine, Center for Innovative Medicine, Wallenberg Academy Fellow, Strategic Research Area Stem Cells and Regenerative Medicine, Vinnova, Stockholm County Council (ALF project), Karolinska Institutet, Crown Princess Margareta's Foundation for the Visually Impaired, ARMEC Lindeberg Foundation, the Ulla och Ingemar Dahlberg Foundation, and King Gustav V and Queen Victoria Foundation, Compton Foundation, the Swiss National Science Foundation grants CRSK-3_190495 and PZ00P3_193445, and grant CZF2019-002427 from the Chan Zuckerberg Initiative. This study was performed at the Live Cell Imaging unit/Nikon Center of Excellence, BioNut, and KI, supported by Knut and Alice Wallenberg Foundation, Swedish Research Council, Centre for Innovative Medicine and the Jonasson donation. Flow cytometry was performed at the MedH Flow Cytometry core facility, and prenatal human tissue was acquired through the Developmental Tissue Bank core facility, both supported by KI/SLL. Sequencing was performed at ESCG Infrastructure in Stockholm at Science for Life Laboratory (funded by the Knut and Alice Wallenberg Foundation and the Swedish Research Council) and Bioinformatics and Expression Analysis (supported by the board of research at the KI and the research committee at the Karolinska Hospital) with assistance from SNIC/Uppsala Multidisciplinary Center for Advanced Computational Science with massively parallel sequencing and access to the UPPMAX computational infrastructure.

Received: March 27, 2022
Revised: May 12, 2022
Accepted: May 13, 2022
Published: June 14, 2022

REFERENCES

Aibar, S., González-Blas, C.B., Moerman, T., Huynh-Thu, V.A., Imrichova, H., Hulselmans, G., Rambow, F., Marine, J.-C., Geurts, P., Aerts, J., et al. (2017). SCENIC: single-cell regulatory network inference and clustering. Nat. Methods *14*, 1083–1086. https://doi.org/10.1038/nmeth.4463.

Ambati, J., Ambati, B.K., Yoo, S.H., Ianchulev, S., and Adamis, A.P. (2003). Age-related macular degeneration: etiology, pathogenesis, and therapeutic strategies. Surv. Ophthalmol. *48*, 257–293. https://doi.org/10.1016/s0039-6257(03)00030-4.

Bartuma, H., Petrus-Reurer, S., Aronsson, M., Westman, S., André, H., and Kvanta, A. (2015). *In vivo* imaging of subretinal bleb-induced outer retinal degeneration in the rabbit. Invest. Ophthalmol. Vis. Sci. *56*, 2423–2430. https://doi.org/10.1167/iovs.14-16208.

Begbie, J. (2013). Induction and patterning of neural crest and ectodermal placodes and their derivatives. Compr. Dev. Neurosci. Patterning Cell Type Specif. Develop. CNS and PNS, 239–258. https://doi.org/10.1016/b978-0-12-397265-1.00212-4.

Bhatia, B., Singhal, S., Jayaram, H., Khaw, P.T., and Limb, G.A. (2010). Adult retinal stem cells revisited. Open Ophthalmol. J. *4*, 30–38. https://doi.org/10.2174/1874364101004010030.

Bosze, B., Hufnagel, R.B., and Brown, N.L. (2020). Chapter 21 - specification of retinal cell types. In Patterning and Cell Type Specification in the Developing CNS and PNS, Second Edition, J. Rubenstein, P. Rakic, B. Chen, and K.Y. Kwan, eds. (Academic Press), pp. 481–504.

Brodie-Kommit, J., Clark, B.S., Shi, Q., Shiau, F., Kim, D.W., Langel, J., Sheely, C., Ruzycki, P.A., Fries, M., Javed, A., et al. (2021). Atoh7-independent specification of retinal ganglion cell identity. Sci. Adv. *7*, eabe4983. https://doi.org/10.1126/sciadv.abe4983.

Choudhary, P., and Whiting, P.J. (2016). A strategy to ensure safety of stem cell-derived retinal pigment epithelium cells. Stem Cell Res. Ther. *7*, 127. https://doi.org/10.1186/s13287-016-0380-6.

Cuomo, A.S.E., Seaton, D.D., McCarthy, D.J., Martinez, I., Bonder, M.J., Garcia-Bernardo, J., Amatya, S., Madrigal, P., Isaacson, A., Buettner, F., et al. (2020). Single-cell RNA-sequencing of differentiating iPS cells reveals dynamic genetic effects on gene expression. Nat. Commun. *11*, 810. https://doi.org/10.1038/s41467-020-14457-z.

David, F.P.A., Litovchenko, M., Deplancke, B., and Gardeux, V. (2020). ASAP 2020 update: an open, scalable and interactive web-based portal for (single-cell) omics analyses. Nucleic Acids Res. *48*, W403–W414. https://doi.org/10.1093/nar/gkaa412.

Fuhrmann, S., Zou, C., and Levine, E.M. (2014). Retinal pigment epithelium development, plasticity, and tissue homeostasis. Exp. Eye Res. *123*, 141–150. https://doi.org/10.1016/j.exer.2013.09.003.

Gao, Z., Mao, C., Pan, P., Mu, X., and Klein, W.H. (2014). Transcriptome of Atoh7 retinal progenitor cells identifies new Atoh7-dependent regulatory genes for retinal ganglion cell formation. Dev Neurobiol *74*, 1123–1140. https://doi.org/10.1002/dneu.22188.

Gehrs, K.M., Anderson, D.H., Johnson, L.V., and Hageman, G.S. (2006). Age-related macular degeneration–emerging pathogenetic and therapeutic concepts. Ann. Med. *38*, 450–471. https://doi.org/10.1080/07853890600946724.

Hu, Y., Wang, X., Hu, B., Mao, Y., Chen, Y., Yan, L., Yong, J., Dong, J., Wei, Y., Wang, W., et al. (2019). Dissecting the transcriptome landscape of the human fetal neural retina and retinal pigment epithelium by single-cell RNA-seq analysis. PLoS Biol. *17*, e3000365. https://doi.org/10.1371/journal.pbio.3000365.

Joshi, R., Mankowski, W., Winter, M., Saini, J.S., Blenkinsop, T.A., Stern, J.H., Temple, S., and Cohen, A.R. (2016). Automated measurement of cobblestone morphology for characterizing stem cell derived retinal pigment epithelial cell cultures. J. Ocul. Pharmacol. Ther. *32*, 331–339. https://doi.org/10.1089/jop.2015.0163.

Kulkarni, A., Anderson, A.G., Merullo, D.P., and Konopka, G. (2019). Beyond bulk: a review of single cell transcriptomics methodologies and applications. Curr. Opin. Biotechnol. *58*, 129–136. https://doi.org/10.1016/j.copbio.2019.03.001.

Kumar, P., Tan, Y., and Cahan, P. (2017). Understanding development and stem cells using single cell-based analyses of gene expression. Development *144*, 17–32. https://doi.org/10.1242/dev.133058.

La Manno, G., Gyllborg, D., Codeluppi, S., Nishimura, K., Salto, C., Zeisel, A., Borm, L.E., Stott, S.R.W., Toledo, E.M., Villaescusa, J.C., et al. (2016). Molecular diversity of midbrain development in mouse, human, and stem cells. Cell *167*, 566–580.e19. https://doi.org/10.1016/j.cell.2016.09.027.

La Manno, G., Soldatov, R., Zeisel, A., Braun, E., Hochgerner, H., Petukhov, V., Lidschreiber, K., Kastriti, M.E., Lönnerberg, P., Furlan, A., et al. (2018). RNA velocity of single cells. Nature *560*, 494–498. https://doi.org/10.1038/s41586-018-0414-6.

La Manno, G., Siletti, K., Furlan, A., Gyllborg, D., Vinsland, E., Mossi Albiach, A., Mattsson Langseth, C., Khven, I., Lederer, A.R., Dratva, L.M., et al. (2021). Molecular architecture of the developing mouse brain. Nature *596*, 92–96. https://doi.org/10.1038/s41586-021-03775-x.

Lederer, A.R., and La Manno, G. (2020). The emergence and promise of single-cell temporal-omics approaches. Curr. Opin. Biotechnol. *63*, 70–78. https://doi.org/10.1016/j.copbio.2019.12.005.

Lidgerwood, G.E., Senabouth, A., Smith-Anttila, C.J.A., Gnanasambandapillai, V., Kaczorowski, D.C., Amann-Zalcenstein, D., Fletcher, E.L., Naik, S.H., Hewitt, A.W., Powell, J.E., and Pebay, A. (2021). Transcriptomic profiling of human pluripotent stem cell-derived retinal pigment epithelium over time. Genomics Proteomics Bioinf. *19*, 223–242. https://doi.org/10.1016/j.gpb.2020.08.002.

Lukowski, S.W., Lo, C.Y., Sharov, A.A., Nguyen, Q., Fang, L., Hung, S.S., Zhu, L., Zhang, T., Grünert, U., Nguyen, T., et al. (2019). A single-cell transcriptome atlas of the adult human retina. EMBO J. *38*, e100811. https://doi.org/10.15252/embj.2018100811.

Maeda, T., Mandai, M., Sugita, S., Kime, C., and Takahashi, M. (2022). Strategies of pluripotent stem cell-based therapy for retinal degeneration: update and challenges. Trends Mol. Med. *28*, 388–404. https://doi.org/10.1016/j.molmed.2022.03.001.

Marquardt, T., Ashery-Padan, R., Andrejewski, N., Scardigli, R., Guillemot, F., and Gruss, P. (2001). Pax6 is required for the multipotent state of retinal progenitor cells. Cell *105*, 43–55. https://doi.org/10.1016/s0092-8674(01)00295-1.

Petrus-Reurer, S., Bartuma, H., Aronsson, M., Westman, S., Lanner, F., André, H., and Kvanta, A. (2017). Integration of subretinal suspension transplants of human embryonic stem cell-derived retinal pigment epithelial cells in a large-eyed model of geographic atrophy. Invest. Opthalmol. Vis. Sci. *58*, 1314. https://doi.org/10.1167/iovs.16-20738.

Petrus-Reurer, S., Bartuma, H., Aronsson, M., Westman, S., Lanner, F., and Kvanta, A. (2018). Subretinal transplantation of human embryonic stem cell derived-retinal pigment epithelial cells into a large-eyed model of geographic atrophy. J. Vis. Exp. *I 131*, 56702. https://doi.org/10.3791/56702.

Plaza Reyes, A., Petrus-Reurer, S., Antonsson, L., Stenfelt, S., Bartuma, H., Panula, S., Mader, T., Douagi, I., André, H., Hovatta, O., et al. (2016). Xeno-free and defined human embryonic stem cell-derived retinal pigment epithelial cells functionally integrate in a large-eyed preclinical model. Stem Cell Rep. *6*, 9–17. https://doi.org/10.1016/j.stemcr.2015.11.008.

Plaza Reyes, A., Petrus-Reurer, S., Padrell Sanchez, S., Kumar, P., Douagi, I., Bartuma, H., Aronsson, M., Westman, S., Lardner, E., Andre, H., et al. (2020a). Identification of cell surface markers and establishment of monolayer differentiation to retinal pigment epithelial cells. Nat. Commun. *11*, 1609. https://doi.org/10.1038/s41467-020-15326-5.

Plaza Reyes, A., Petrus-Reurer, S., Sánchez, S.P., Kumar, P., Douagi, I., Bartuma, H., Aronsson, M., Westman, S., Lardner, E., Falk, A., et al. (2020b). Xeno-free, chemically defined and scalable monolayer differentiation protocol for retinal pigment epithelial cells. Protoc. Exch. https://doi.org/10.21203/rs.3.pex-635/v1.

Quake, S.R., and Sapiens Consortium, T. (2021). The Tabula Sapiens: a single cell transcriptomic atlas of multiple organs from individual human donors. Preprint at bioRxiv. https://doi.org/10.1101/2021.07.19.452956.

Rodin, S., Antonsson, L., Niaudet, C., Simonson, O.E., Salmela, E., Hansson, E.M., Domogatskaya, A., Xiao, Z., Damdimopoulou, P., Sheikhi, M., et al. (2014). Clonal culturing of human embryonic stem cells on laminin-521/E-cadherin matrix in defined and xeno-free environment. Nat Commun *5*, 3195.. https://doi.org/10.1038/ncomms4195.

Shao, J., Zhou, P.-Y., and Peng, G.-H. (2017). Experimental study of the biological properties of human embryonic stem cell-derived retinal progenitor cells. Sci. Rep. *7*, 42363. https://doi.org/10.1038/srep42363.

Streit, A. (2007). The preplacodal region: an ectodermal domain with multipotential progenitors that contribute to sense organs and cranial sensory ganglia. Int. J. Dev. Biol. *51*, 447–461. https://doi.org/10.1387/ijdb.072327as.

Sunness, J.S. (1999). The natural history of geographic atrophy, the advanced atrophic form of age-related macular degeneration. Mol. Vis. 5, 25.

Voigt, A.P., Mulfaul, K., Mullin, N.K., Flamme-Wiese, M.J., Giacalone, J.C., Stone, E.M., Tucker, B.A., Scheetz, T.E., and Mullins, R.F. (2019). Single-cell transcriptomics of the human retinal pigment epithelium and choroid in health and macular degeneration. Proc. Natl. Acad. Sci. U S A *116*, 24100–24107. https://doi.org/10.1073/pnas.1914143116.

Wang, Y., Tang, Z., and Gu, P. (2020). Stem/progenitor cell-based transplantation for retinal degeneration: a review of clinical trials. Cell Death Dis. *11*, 793. https://doi.org/10.1038/s41419-020-02955-3.

Wolf, F.A., Angerer, P., and Theis, F.J. (2018). SCANPY: large-scale single-cell gene expression data analysis. Genome Biol. *19*, 15. https://doi.org/10.1186/s13059-017-1382-0.

Zarbin, M., Sugino, I., and Townes-Anderson, E. (2019). Concise review: update on retinal pigment epithelium transplantation for age-related macular degeneration. Stem Cells Transl. Med. *8*, 466–477. https://doi.org/10.1002/sctm.18-0282.

Zeisel, A., Hochgerner, H., Lönnerberg, P., Johnsson, A., Memic, F., van der Zwan, J., Häring, M., Braun, E., Borm, L.E., La Manno, G., et al. (2018). Molecular architecture of the mouse nervous system. Cell *174*, 999–1014. https://doi.org/10.1016/j.cell.2018.06.021.

Stem Cell Reports, Volume *17*

Supplemental Information

Molecular profiling of stem cell-derived retinal pigment epithelial cell differentiation established for clinical translation

Sandra Petrus-Reurer, Alex R. Lederer, Laura Baqué-Vidal, Iyadh Douagi, Belinda Pannagel, Irina Khven, Monica Aronsson, Hammurabi Bartuma, Magdalena Wagner, Andreas Wrona, Paschalis Efstathopoulos, Elham Jaberi, Hanni Willenbrock, Yutaka Shimizu, J. Carlos Villaescusa, Helder André, Erik Sundström, Aparna Bhaduri, Arnold Kriegstein, Anders Kvanta, Gioele La Manno, and Fredrik Lanner

SUPPLEMENTAL ITEMS

Molecular profiling of stem cell-derived retinal pigment epithelial cell differentiation established for clinical translation

Petrus-Reurer, Lederer et al.

Supplemental Figures 1 – 7

Supplemental Tables 1 – 6

Experimental Procedures

SUPPLEMENTAL FIGURES

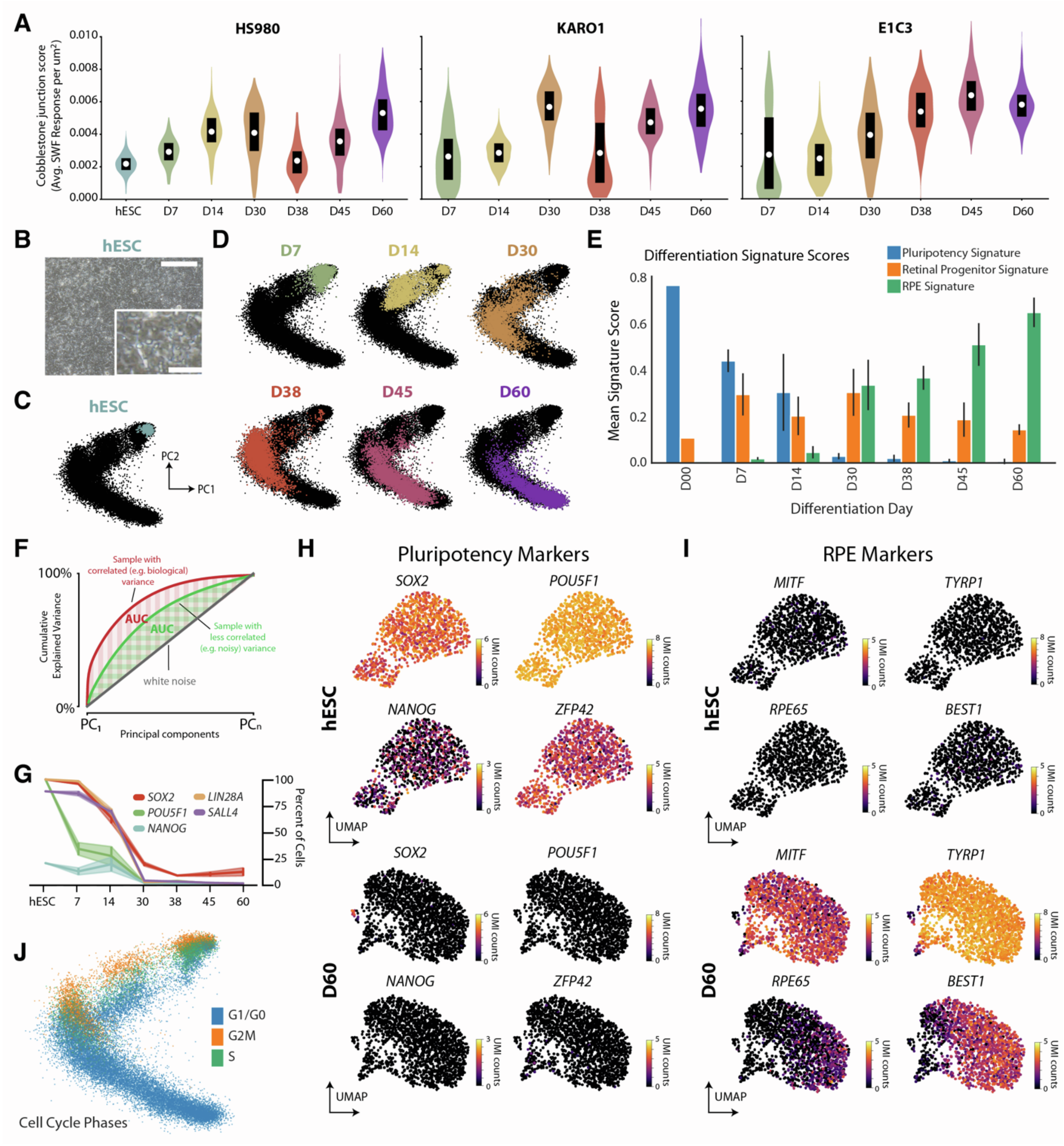

Figure S1. Cellular heterogeneity analysis of hESC-RPE differentiation. Related to Figure 1. (A) Graphs showing quantification of cobblestone morphology throughout differentiation in the HS980, KARO1, and E1C3 cell lines using the junction score methodology and software developed by Joshi et al., 2016. **(B)** Brightfield image of undifferentiated hESCs in the HS980 line. Scale bars: 100μm; inset 20μm. **(C)** Principal component (PC) representation of hESCs in the HS980 line. **(D)** PC representation of *in vitro* hESC-RPE time points across three lines, colored by day. **(E)** Bar graph of average pluripotency, retinal progenitor and RPE signature scores by differentiation day. Error bars represent standard deviation of the mean over three cell line replicates. **(F)** Schematic of AUC variance evaluation metric. **(G)** Graph showing the percentage of cells positive (>0.5 normalized UMI counts) for pluripotency marker genes at each time point. **(H, I)** UMAPs showing normalized gene expression of pluripotent stem cells markers (H) and RPE markers (I) in undifferentiated hESCs and at D60. **(J)** Principal component representation of hESC-RPE differentiation across all lines colored by assigned cell cycle phase.

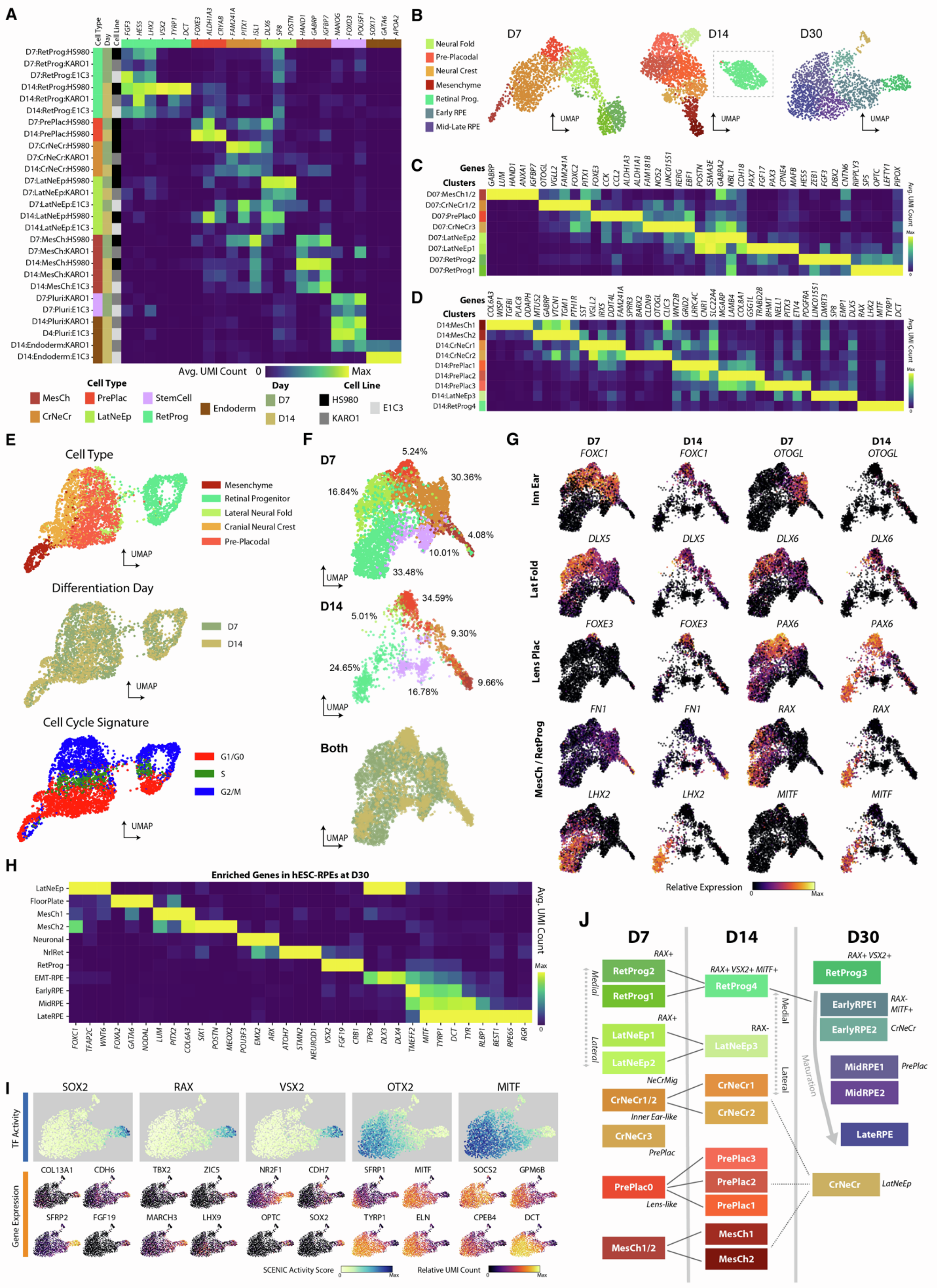
A
Cell Type
Day
Cell Line
D7:RetProg:HS980
D7:RetProg:KARO1
D7:RetProg:E1C3
D14:RetProg:HS980
D14:RetProg:KARO1
D14:RetProg:E1C3
D7:PrePlac:HS980
D14:PrePlac:HS980
D7:CrNeCr:HS980
D7:CrNeCr:KARO1
D14:CrNeCr:HS980
D7:LatNeEp:HS980
D7:LatNeEp:KARO1
D7:LatNeEp:E1C3
D14:LatNeEp:HS980
D14:LatNeEp:E1C3
D7:MesCh:HS980
D7:MesCh:KARO1
D14:MesCh:HS980
D14:MesCh:KARO1
D14:MesCh:E1C3
D7:Pluri:KARO1
D7:Pluri:E1C3
D14:Pluri:KARO1
D4:Pluri:E1C3
D14:Endoderm:KARO1
D14:Endoderm:E1C3
Avg. UMI Count 0 Max
Cell Type
MesCh
PrePlac
StemCell
CrNeCr
LatNeEp
RetProg
Endoderm
Day
D7
D14
Cell Line
HS980
KARO1
E1C3
B
Neural Fold
Pre-Placodal
Neural Crest
Mesenchyme
Retinal Prog.
Early RPE
Mid-Late RPE
D7
D14
D30
UMAP
C
Genes
Clusters
D07:MesCh1/2
D07:CrNeCr1/2
D07:PrePlac0
D07:CrNeCr3
D07:LatNeEp2
D07:LatNeEp1
D07:RetProg2
D07:RetProg1
Avg. UMI Count
D
D14:MesCh1
D14:MesCh2
D14:CrNeCr1
D14:CrNeCr2
D14:PrePlac1
D14:PrePlac2
D14:PrePlac3
D14:LatNeEp3
D14:RetProg4
E
Cell Type
Mesenchyme
Retinal Progenitor
Lateral Neural Fold
Cranial Neural Crest
Pre-Placodal
Differentiation Day
Cell Cycle Signature
G1/G0
S
G2/M
F
D7
5.24%
30.36%
16.84%
4.08%
10.01%
33.48%
D14
34.59%
5.01%
9.30%
24.65%
9.66%
16.78%
Both
G
Inn Ear
Lat Fold
Lens Plac
MesCh / RetProg
FOXC1
OTOGL
DLX5
DLX6
FOXE3
PAX6
FN1
RAX
LHX2
MITF
Relative Expression
H
Enriched Genes in hESC-RPEs at D30
LatNeEp
FloorPlate
MesCh1
MesCh2
Neuronal
NrlRet
RetProg
EMT-RPE
EarlyRPE
MidRPE
LateRPE
Avg. UMI Count
I
SOX2
RAX
VSX2
OTX2
MITF
TF Activity
Gene Expression
COL13A1
CDH6
TBX2
ZIC5
NR2F1
CDH7
SFRP1
SOCS2
GPM6B
SFRP2
FGF19
MARCH3
LHX9
OPTC
TYRP1
ELN
CPEB4
DCT
SCENIC Activity Score
Relative UMI Count
J
D7
D14
D30
RAX+
RetProg2
RetProg1
RAX+ VSX2+ MITF+
RetProg4
RAX+ VSX2+
RetProg3
Medial
Lateral
LatNeEp1
LatNeEp2
LatNeEp3
RAX-
EarlyRPE1
EarlyRPE2
RAX-
MITF+
CrNeCr
NeCrMig
CrNeCr1/2
Inner Ear-like
CrNeCr1
CrNeCr2
CrNeCr3
PrePlac
MidRPE1
MidRPE2
Maturation
LateRPE
PrePlac0
Lens-like
PrePlac1
PrePlac2
PrePlac3
CrNeCr
LatNeEp
MesCh1/2
MesCh1
MesCh2

Figure S2. Gene expression characterization and canonical correlation analysis of early differentiation. Related to Figure 2. (A) Heatmap of enriched genes by primary clusters, grouped by cell line, at D7 and D14 of hESC-RPE differentiation. **(B)** UMAP representation of HS980 differentiation at D7, D14, and D30, colored by cell type. **(C)** Heatmap of top enriched genes of each cell type cluster at D7. **(D)** Heatmap of top enriched genes of each cell type cluster at D14. **(E)** D7 and D14 cell HS980 populations projected on a shared low dimensional subspace using canonical correlation analysis (CCA; see Experimental Procedures), colored by cell type, differentiation day, and cell cycle phase. **(F)** UMAP representation of D7 and D14 cells, across all three lines, integrated with CCA. **(G)** UMAPs showing gene expression in all three lines of fundamental cell type markers for Inner Ear (InnEar), Lateral Fold (LatFold), Lens Placode (LensPlac), Mesenchyme (MesCh), and Retinal Progenitor (RetProg) from D7 to D14. Expression of neural crest inner ear (*FOXC1*, *OTOGL*) and lateral fold (*DLX5*, *DLX6*) markers decreases from D7 to D14. **(H)** Heatmap of enriched genes by cell type at D30 across all three lines. **(I)** Top: Transcription factor (TF) activity scores for SOX2, RAX, VSX2, OTX2, and MITF obtained by SCENIC analysis. Bottom: UMAPs showing gene expression of the top four inferred target genes of each TF at D30 of differentiation in HS980. **(J)** Schematic of the proposed relationship among the various secondary clusters during pigmentation induction. Edges indicate putative relationships between cell types identified at different time points.

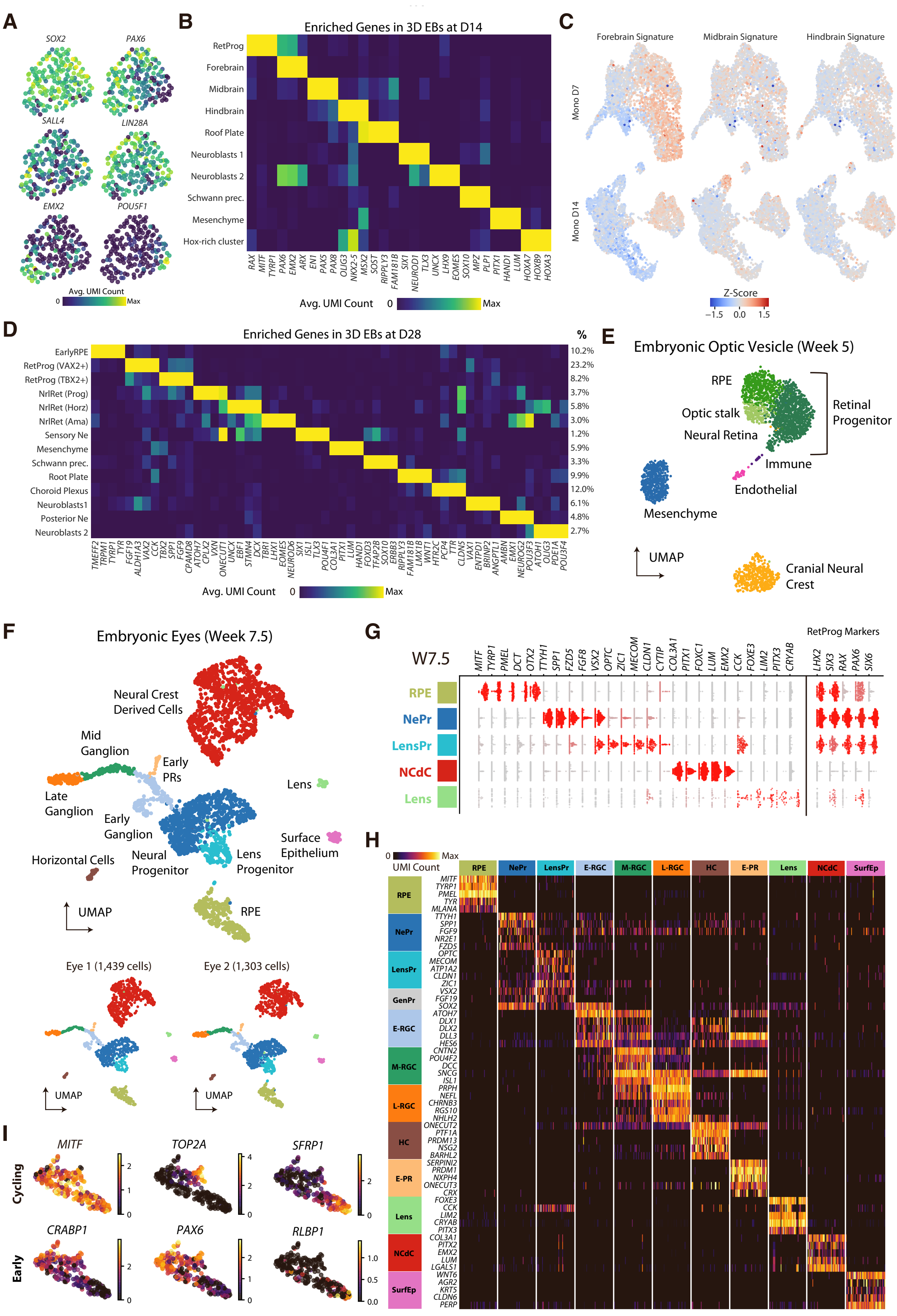
A
SOX2
PAX6
SALL4
LIN28A
EMX2
POU5F1
Avg. UMI Count
0
Max
B
Enriched Genes in 3D EBs at D14
RetProg
Forebrain
Midbrain
Hindbrain
Roof Plate
Neuroblasts 1
Neuroblasts 2
Schwann prec.
Mesenchyme
Hox-rich cluster
Avg. UMI Count
0
Max
C
Forebrain Signature
Midbrain Signature
Hindbrain Signature
Mono D7
Mono D14
Z-Score
−1.5 0.0 1.5
D
Enriched Genes in 3D EBs at D28
%
EarlyRPE 10.2%
RetProg (VAX2+) 23.2%
RetProg (TBX2+) 8.2%
NrlRet (Prog) 3.7%
NrlRet (Horz) 5.8%
NrlRet (Ama) 3.0%
Sensory Ne 1.2%
Mesenchyme 5.9%
Schwann prec. 3.3%
Root Plate 9.9%
Choroid Plexus 12.0%
Neuroblasts1 6.1%
Posterior Ne 4.8%
Neuroblasts 2 2.7%
Avg. UMI Count
0
Max
E
Embryonic Optic Vesicle (Week 5)
RPE
Optic stalk
Neural Retina
Retinal Progenitor
Immune
Endothelial
Mesenchyme
UMAP
Cranial Neural Crest
F
Embryonic Eyes (Week 7.5)
Neural Crest Derived Cells
Mid Ganglion
Early PRs
Lens
Late Ganglion
Early Ganglion
Surface Epithelium
Horizontal Cells
Neural Progenitor
Lens Progenitor
UMAP
RPE
Eye 1 (1,439 cells)
Eye 2 (1,303 cells)
UMAP
UMAP
G
W7.5
RetProg Markers
RPE
NePr
LensPr
NCdC
Lens
H
0
Max
UMI Count
RPE
NePr
LensPr
E-RGC
M-RGC
L-RGC
HC
E-PR
Lens
NCdC
SurfEp
GenPr
I
Cycling
Early
MITF
TOP2A
SFRP1
CRABP1
PAX6
RLBP1

Figure S3. Characterization of RPE differentiation in 3D embryoid bodies and compared to embryonic references. Related to Figure 3. **(A)** UMAP representation of EB differentiation a D7 (181 cells), colored by normalized UMI count of progenitor (*SOX2, PAX6*), pluripotency (*SALL4, LIN28A*) and regional (*EMX2*, *POU5F1*) marker genes. **(B)** Heatmap of enriched genes by cell type at EB at D14. **(C)** Signature scores for forebrain, midbrain and hindbrain visualized on D7 and D14 monolayer cells. Scores were computed as those in Figure 3B. **(D)** Heatmap of enriched genes by cell type at EB at D28, with percent composition of total EB population. **(E)** UMAP representation of a human embryonic optic vesicle (2,637 cells) dissected at 5 weeks (Carnegie Stage 13). Cluster identities include: optic cell types derived from retinal progenitors (RetProg), such as retinal pigment epithelium (RPE), neural retina (NR), and optic stalk (OS), in addition to periocular mesenchyme (MesCh), cranial neural crest (CrNeCr), immune, and smooth muscle. **(F)** Top: UMAP representation of scRNA-seq data from two human fetal eyes at week 7.5, colored by cell type. Bottom: UMAP of each individual fetal eye separately. **(G)** Violin plots of enriched genes in the identified W7.5 clusters. **(H)** Heatmap of normalized enriched gene expression. Genes were selected using an enrichment score by cell type in (F). **(I)** Retinal progenitor and RPE log2 normalized gene expression of cycling, Early and Mid RPE markers in the RPE cell cluster from (F).

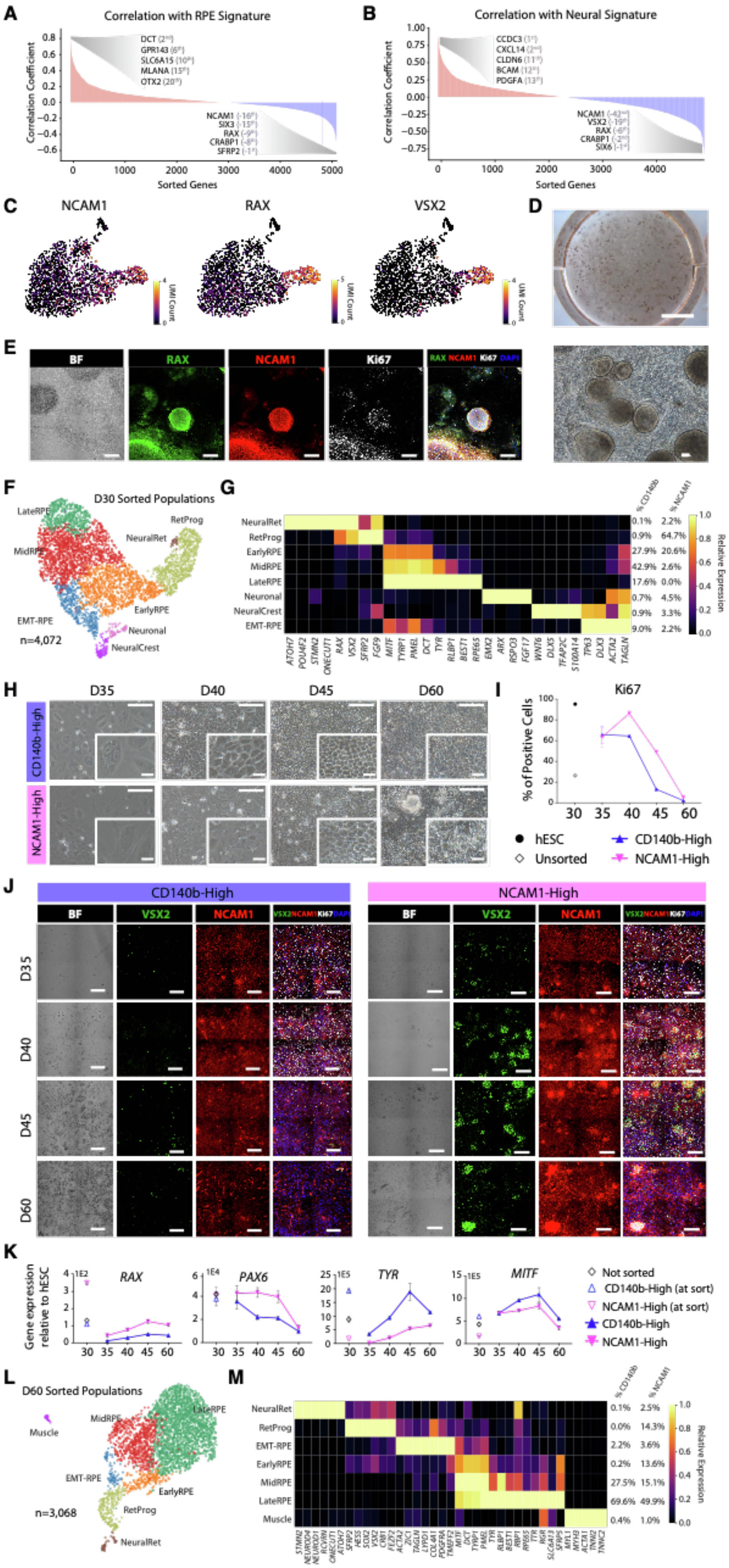
A
Correlation with RPE Signature
Correlation Coefficient
Sorted Genes
B
Correlation with Neural Signature
Correlation Coefficient
Sorted Genes
C
NCAM1
RAX
VSX2
UMI Count
D
E
BF
RAX
NCAM1
Ki67
RAX NCAM1 Ki67 DAPI
F
D30 Sorted Populations
LateRPE
RetProg
NeuralRet
MidRPE
EarlyRPE
EMT-RPE
Neuronal
NeuralCrest
n=4,072
G
% CD140b
% NCAM1
Relative Expression
H
D35
D40
D45
D60
CD140b-High
NCAM1-High
I
Ki67
% of Positive Cells
hESC
Unsorted
CD140b-High
NCAM1-High
J
CD140b-High
NCAM1-High
BF
VSX2
NCAM1
VSX2 NCAM1 Ki67 DAPI
D35
D40
D45
D60
K
Gene expression relative to hESC
RAX
PAX6
TYR
MITF
Not sorted
CD140b-High (at sort)
NCAM1-High (at sort)
CD140b-High
NCAM1-High
L
D60 Sorted Populations
Muscle
MidRPE
LateRPE
EMT-RPE
EarlyRPE
RetProg
NeuralRet
n=3,068
M
% CD140b
% NCAM1
Relative Expression

Figure S4. Characterization of CD140b-High and NCAM1-High sorted populations exposed to RPE differentiation conditions. Related to Figure 4. (A-B) Pearson's correlation coefficients were computed between 5,412 and an RPE signature (A) or 4,664 genes and a neural signature (B) using normalized counts and cells belonging to the D30 retinal progenitor and RPE clusters or retinal progenitor and neural clusters, respectively. **(C)** UMAPs showing gene expression of progenitor markers in scRNA-seq hESC-RPE at D30 in the HS980 cell line. **(D)** Camera pictures of hESC-RPE D30 cultures. **(E)** Brightfield and immunofluorescence stainings of hESC-RPE D30 cells showing co-expression of RAX, NCAM1 and Ki67 markers. Scale bars: top 1mm; bottom 100µm. **(F)** scRNA-seq of 4,072 single cells from NCAM1-High sorted, CD140b-High sorted, and unsorted D30 cells, colored by cell type. See Figure 4E for composition by sample identifier. **(G)** Heatmap of enriched marker genes in cell types of sorted populations at D30, with cell type composition percentages for both sorted populations. **(H)** Brightfield images of unsorted, CD140b-High and NCAM1-High populations at D33, D40, D45, and D60. FACS sorting of the two populations was performed at D30. Scale bars: 100µm; inset 20µm. **(I)** Graph showing the percentage of positive cells expressing the Ki67 proliferation marker in hESC, unsorted, CD140b-High and NCAM1-High populations at the moment of FACS sorting (D30) and D35, D40, D45, and D60. Bars represent mean +/-SEM from three independent experiments. **(J)** Brightfield and immunofluorescence images showing expression of VSX2 and NCAM1 in unsorted, CD140b-High and NCAM1-High populations after FACS sorting at differentiation D35, D40, D45, and D60. Scale bars: 200µm. **(K)** Graphs representing RT-qPCR of retinal progenitor (*RAX*, *PAX6*) and RPE (*MITF*, *TYR*) marker genes in unsorted, CD140b-High and NCAM1-High populations at the moment of sort and at post-sort D30, 35, 40, 45, and 60. **(L)** scRNA-seq of 3,068 single cells from NCAM1-High sorted, CD140b-High sorted, and unsorted D60 cells, colored by cell type. See Figure 4K for composition by sample identifier. **(M)** Heatmap of enriched marker genes in cell types of sorted populations at D60, with cell type composition percentages for both sorted populations.

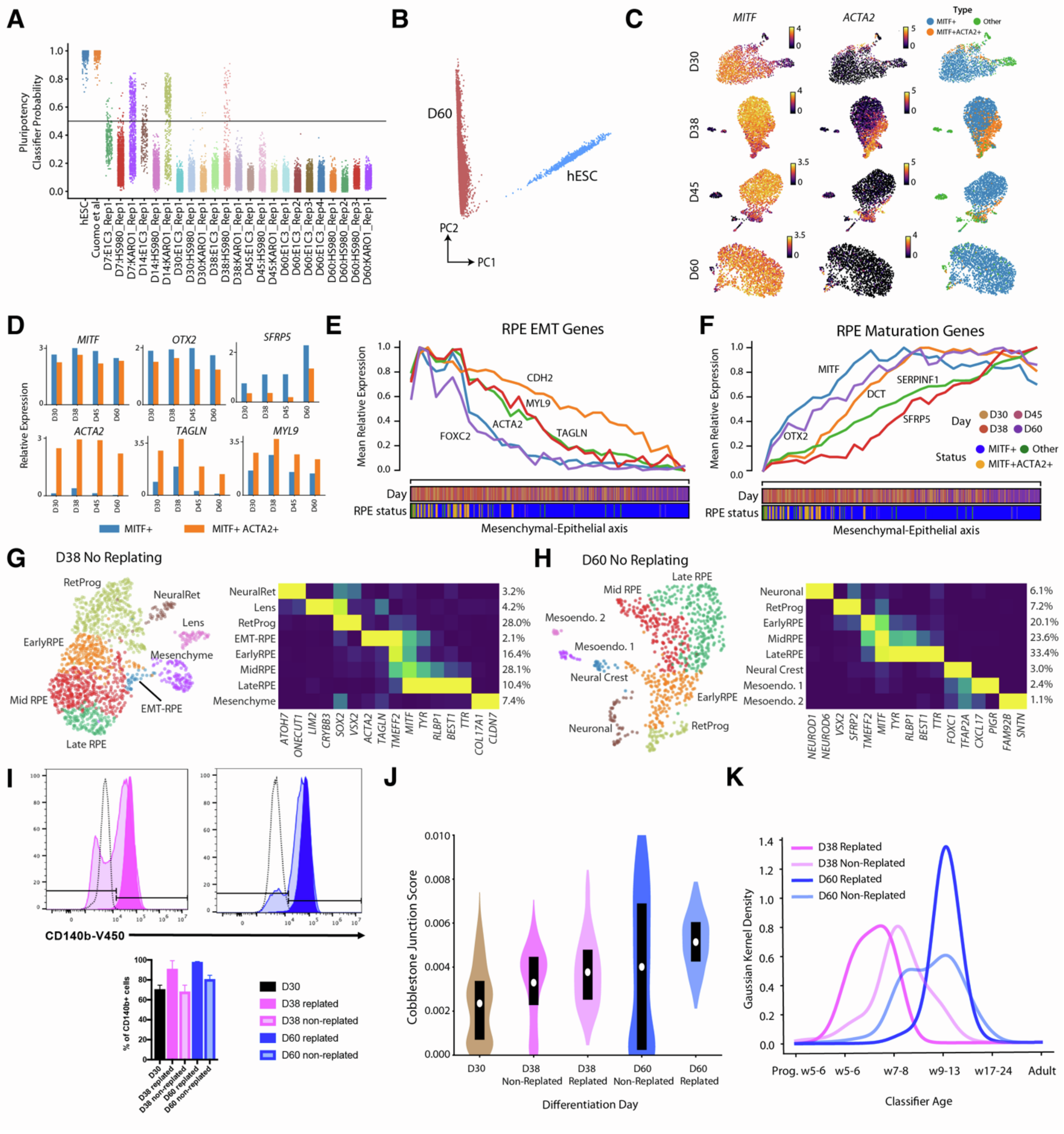

Figure S5. Characterization of late differentiation and overall gene expression correlation. Related to Figure 6. (**A**) Plot displaying the probability outputted by a pluripotency classifier when inputted data from *in vitro* cells at all time points. A random forest classifier was trained a mixture of hESCs from this and another study (Cuomo et al 2020; see Experimental Procedures) (**B**) Scatter plot of the two first principal components of all D60 and hESC *in vitro* cells. (**C**) UMAP overlayed with *MITF* and *ACTA2* gene

expression at D30, D38, D45, and D60. Cells are labeled as *MITF+* (maturing RPE), *MITF+ACTA2+* (EMT-RPE), or other (non-RPE cell types). (**D**) Bar graphs showing the gene expression differences between EMT-RPE and maturing RPE. EMT-RPE expresses EMT markers *ACTA2, TAGLN,* and *MYL9* more highly, whereas RPE markers *MITF, OTX2,* and *SFRP5* are more highly expressed in non-transitioning RPE. (**E-F**) Line plots showing average expression of RPE-EMT (E) and mature RPE (F) along a mesenchymal-epithelial axis of variation determined by fitting a principal curve (see Experimental Procedures). Colored bars on the x-axis indicate time point and RPE status of cells along the axis. (**G**) Left: UMAP representation of 1,423 single cells at hESC-RPE D38 without replating at D30. Right: heatmap of enriched genes by cell type. (**H**) Left: UMAP representation of 772 single cells at hESC-RPE D60 without replating at D30. Right: heatmap of enriched genes by cell type. (**I**) Top: representative flow cytometry plots for HS980 cell line showing CD140b cell surface marker expression at D38 and D60 for replated and non-replated conditions. Dotted lines represent hESC (negative control). Bottom: bar graphs show the average of CD140b marker expression in the stated conditions and time points for both HS980 and KARO1 cell lines. (**J**) Violin plot displaying the quantification of cobblestone morphology at D38 and D60 with and without replating in the HS980 cell lines using the junction score methodology and software developed by Joshi et al, J Ocul Pharmacol Ther. 2016. (**K**) Graph showing distribution of classifications of D38 and D60 RPE cultures, with and without replating (see also Figures 6 and S6). Bars represent mean +/-SEM from three independent experiments.

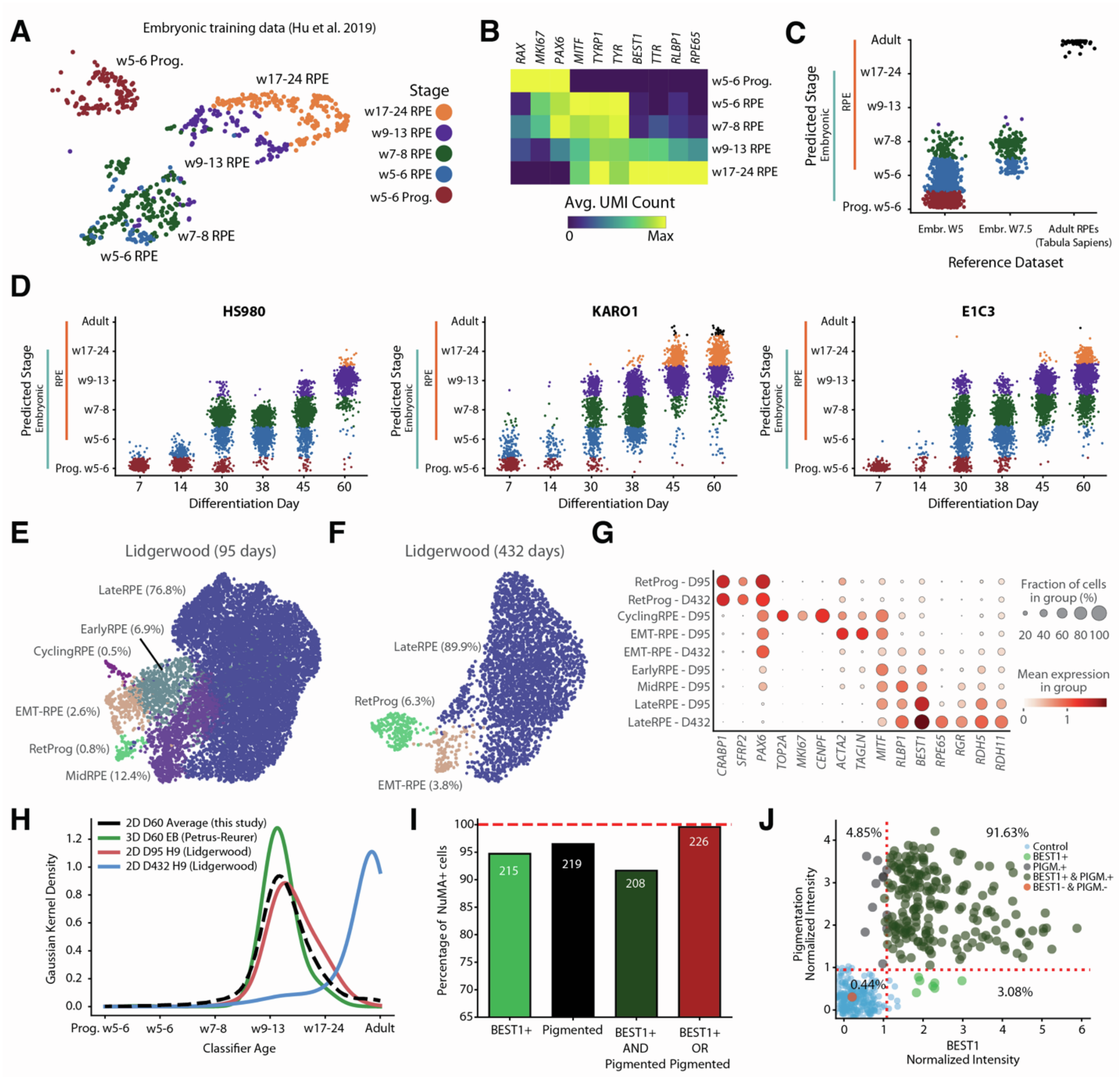

Figure S6. Ordinal classification of *in vitro* hESC-RPE. Related to Figure 6. (A) UMAP representation of Hu et al dataset of 783 human fetal cells from various time points in development, as used in the ordinal classifier (see Figure 7). Cells were colored into five categories for classification. **(B)** Heatmap showing an overview of uniquely enriched retinal progenitor and RPE marker genes in the data in (D). **(C)** Plot showing ordinal classification of reference embryonic RPEs at weeks 5 and 7.5 (see Figures 3 and S3). **(D)** Plots showing ordinal classification for hESC-RPE differentiation data in the HS980, KARO1, and E1C3 cell lines individually. **(E)** UMAP representation of RPE differentiation

day 90 in H9 cell line (9,456 single cells) re-analyzed from Lidgerwood et al, Genomics Proteomics Bioinformatics 2020. Cells colored and labeled by newly-annotated cell types. **(F)** UMAP representation of RPE differentiation day 432 (1 Year) in H9 cell line (3,216 single cells) re-analyzed from Lidgerwood et al, Genomics Proteomics Bioinformatics 2020. Cells colored and labeled by newly-annotated cell types. **(G)** Dot plot of marker gene expression for RetProg, CyclingRPE, EMT-RPE, EarlyRPE, MidRPE, and LateRPE in (A-B). **(H)** Graph showing distribution of ordinal classifications of various differentiated RPE culture protocols, including the 2D monolayer protocol from this study (20,682 cells), 3D EBs at D60 (Petrus-Reurer et al, 2020; 294 cells), 2D D95 H9 (Lidgerwood et al., 2021; 9,456 cells) and 2D D432 H9 (Lidgerwood et al., 2021; 3,216 cells). **(I)** Bar graph of 227 hESC-RPE grafted cell BEST1 and pigmentation statuses after 30 days into the albino rabbit subretinal space. NuMA+ human cells from ten sections and three rabbits were manually segmented and assessed by immunofluorescence (BEST1 expression) and brightfield (Pigmentation). **(J)** Scatter plot of the BEST1 normalized intensity and the pigmentation normalized intensity for 227 grafted NuMA+ cells and 227 NuMA- control cells. Cells are colored by histological status (Control, BEST1+, PIGM+, BEST1 & PIGM+, and BEST1- & PIGM-). Red dotted lines indicate the 97.5th percentile threshold of the signal observed in the negative control cells.

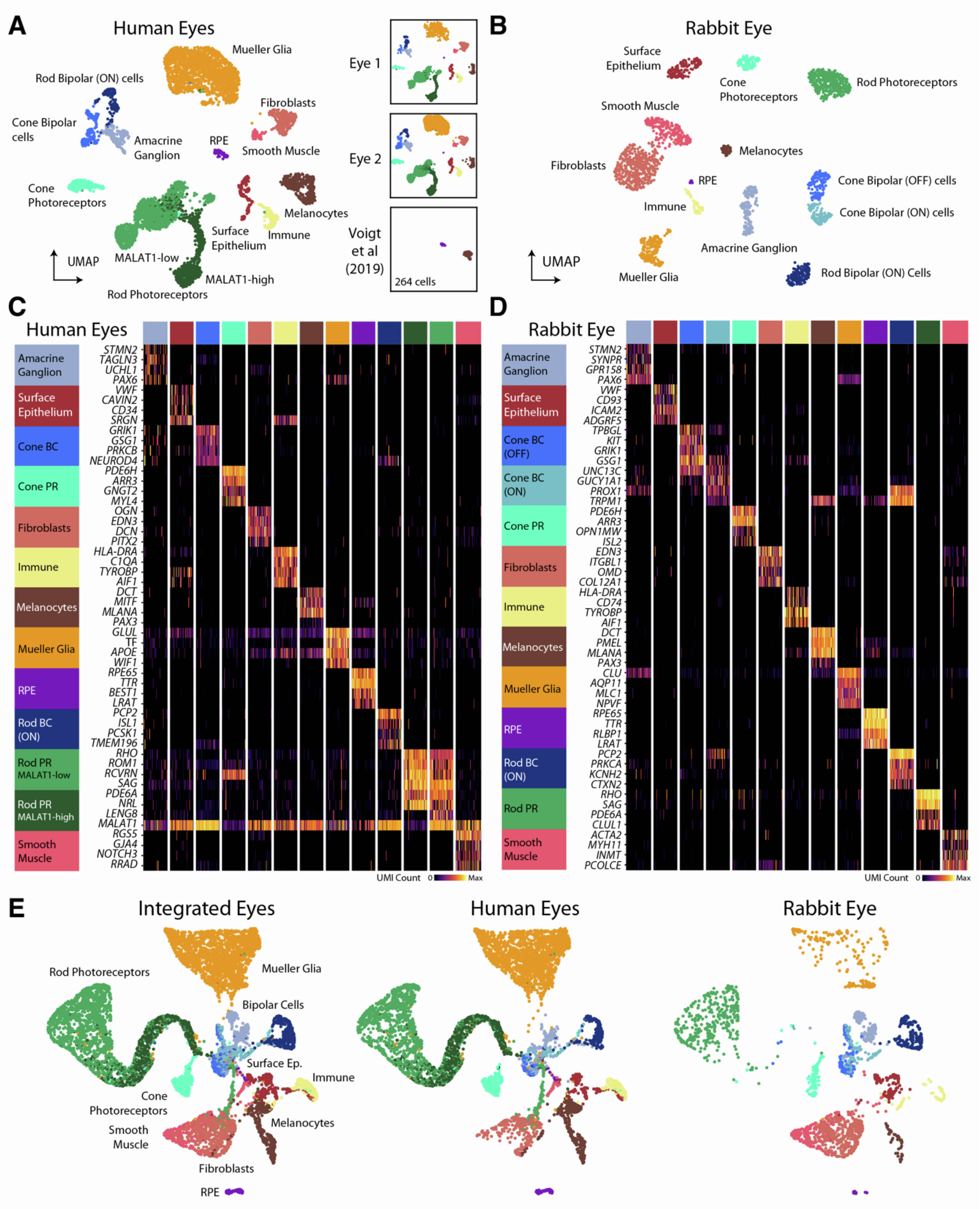

Figure S7. Transcriptional analysis of albino rabbit and human retinas. Related to Figure 7. (A) Annotated UMAP representation of 5,538 human cells from two human

eyes (1,564 cells and 3,706 cells), categorized into 13 different cell types. Additional RPEs and melanocytes (264 cells) were re-analyzed and incorporated from Voigt et al, 2019. **(B)** Annotated UMAP representation of 1,965 rabbit cells categorized into 13 different retina cell types. **(C)** Heatmap of enriched marker genes for adult eye cell types. **(D)** Heatmap of enriched marker genes for rabbit eye cell types. Genes were selected from among the top 20 enriched genes per cluster for (C) and (D). **(E)** CCA integration of human and rabbit eyes. Integration was performed using Seurat on 2,000 enriched genes from a total of 9,889 genes with shared annotations between the two species (see Experimental Procedures).

SUPPLEMENTAL TABLES

Table S1. Overview of all scRNA-seq samples generated and quality control conditions. Related to Experimental Procedures.

See attached TableS1.xlsx file.

Table S2. Gene enrichment during hESC-RPE pigmentation induction (D7, D14, and D30) by cell type and time point. Related to Figure 2.

See attached TableS2.xlsx file.

Table S3. Gene enrichment for embryonic eyes at Carnegie Stages 12, 13, 14, 15, and 20 by cell type and time point. Related to Figure 3.

See attached TableS3.xlsx file.

Table S4. Gene correlation scores with retinal progenitor and neural tube signature during the identification of NCAM1 cell surface marker. Related to Figure 4.

See attached TableS4.xlsx file.

Table S5. Gene enrichment during late hESC-RPE differentiation (D38, D45, D60) by cell type. Related to Figure 6.

See attached TableS5.xlsx file.

Table S6. Gene enrichment scores for adult human and rabbit eyes by cell type. Related to Figure S7.

See attached TableS6.xlsx file.

EXPERIMENTAL PROCEDURES, Related to Experimental Procedures.

RESOURCE AVAILABILITY

Lead Contact

Further information and requests for resources and reagents should be directed to and will be fulfilled by the Lead Contact, Fredrik Lanner (fredrik.lanner@ki.se).

MATERIALS AVAILABILITY

This study did not generate new unique reagents.

DATA AND CODE AVAILABILITY

All scRNA-seq datasets generated for this study are available as raw feature-count matrices, as generated by CellRanger, at the accession number GSE164092. Raw FASTQ files for all samples are also available, with exception of those from E1C3 cell line, for which specific approval will be needed from Novo Nordisk. Processed loom/h5ad files containing cell type annotations, UMAP embedding coordinates, and other metadata as well as the custom reference genome used for human and rabbit pooled analysis can be downloaded at the GEO accession above. Enriched gene lists, correlation scores, and other metadata are provided as Supplemental Tables. Jupyter notebooks (in both ipynb and html formats) to reproduce the single cell analyses performed in this study, as well as all custom companion source code, are shared at https://github.com/lamanno-epfl/rpe_differentiation_profiling_code. Datasets are available for interactive visualization and analysis using the ASAP web resource (https://asap.epfl.ch/) under public keys ASAP 75-90 (David et al., 2020; Gardeux et al., 2017). Human embryonic reference datasets of optic vesicle/cup from Carnegie Stages 12, 13, 14, and 15 can be downloaded at https://data.nemoarchive.org/biccn/lab/kriegstein/transcriptome/. The external scRNA-seq reference datasets used in this study can be found at the original accession numbers: GSE135922 (Voigt et al., 2019) GSE107618 (Hu et al., 2019), GSE116106 (Lu et al., 2020) or on ArrayExpress at E-MTAB-8511 (Lidgerwood et al., 2021).

hESC CELL CULTURE

HS980 and KARO1 were previously derived and cultured under xeno-free and defined conditions (Rodin et al., 2014) following informed consent by donors (Swedish Ethical Review Authority: 2011/745:31/3). E1C3 (NN GMP0050E1C3) was provided as a research cell bank of the clinical GMP cell line by NovoNordisk (UCSF IRB: 1518222, for RPE differentiation Projekt-ID: H-18016740, Anmeldelsesnr.: 73105). All hESCs maintain a normal karyotype and trilineage differentiation potential, and are routinely tested for mycoplasma. Cells were maintained on hrLN-521 (10µg/mL, Biolamina) or iMatrix-511 (0.25 µg/cm^2, Nippi, T303) in NutriStem hPSC XF medium (Biological Industries) and a 5% CO_2/5% O_2 incubator.

Cells were passaged enzymatically at a 1:10 ratio every 5-6 days. Confluent cultures were washed twice with PBS without Ca^{2+} and Mg^{2+} and incubated for 5 min at 37°C, 5% CO_2/5% O_2 with TrypLE Select (ThermoFisher Scientific, 12563011). The enzyme was then carefully removed and cells were collected in fresh pre-warmed NutriStem hPSC XF medium by gentle pipetting to obtain a single cell suspension. Cells were centrifuged at 300 g for 4 min, the pellet resuspended in fresh prewarmed NutriStem hPSC XF medium, and plated on a freshly hrLN-521 coated dish. Two days after passage, the medium was replaced with fresh pre-warmed NutriStem hPSC XF medium and changed daily.

2D and 3D hESC-RPE *in vitro* DIFFERENTIATION

A step-by-step protocol describing the differentiation procedure has been reported previously (Plaza Reyes et al., 2020a, 2020b). hESCs were plated at a cell density of 2.4x10^4 cells/cm^2 on hrLN-521 (20 µg/mL) using NutriStem hPSC XF medium. A Rho-kinase inhibitor (Millipore, Y-27632) at a concentration of 10 µM was added during the first 24h, while cells were kept at 37°C, 5% CO_2/5% O_2. After 24h, hPSC medium was replaced with differentiation medium NutriStem hPSC XF without bFGF and TGFβ and cells were placed at 37°C, 5% CO_2/21%O_2. From day 6 after plating, 100 ng/mL of Activin A (R&D Systems, 338-AC-050) was added to the media. Cells were fed three times a week and kept for 30 days. Cells that did not undergo replating after 30 days were kept in these conditions for 60 days, but without the addition of Activin A from day 30 until day

of analysis. During standard differentiations, with replating, monolayers were then trypsinized using TrypLE Select (ThermoFisher Scientific, 12563011) for 10 min at 37°C, 5% CO_2. The enzyme was carefully removed and the cells were collected in fresh pre-warmed NutriStem hPSC XF medium without bFGF and TGFβ by gentle pipetting to obtain a single cell suspension. Cells were centrifuged at 300 g for 4 min, the pellet was resuspended, passed through a cell strainer (ø 40 µm, VWR, 732-2757) and seeded on laminin coated dishes (hrLN-521 at 20µg/mL) at $6.8x10^4$ cells/cm^2. Replated cells were fed three times a week during the subsequent 30 days with NutriStem hPSC XF medium without bFGF and TGFβ. Brightfield images were acquired with a Nikon Eclipse TE2000-S microscope and a Canon SX170 IS camera was used to capture pigmentation from the top of the wells.

3D hESC-RPE Embryoid Body (EB) differentiations were cultured following the differentiation procedure reported previously (Plaza Reyes et al., 2016). Pluripotent stem cells (HS980 line) were cultured to confluence on hrLN-521-coated plates and manually scraped to produce EBs using a 1000 µl pipette tip. The EBs were then cultured in suspension in low attachment plates at a density of $5\text{-}7x10^4$ cells/cm^2. Differentiation was performed in NutriStem hPSC XF medium without bFGF and TGFβ with media changed twice a week for 28-30 days. 10 µM Rho-kinase inhibitor (Millipore Y-27632) was added to the suspension cultures only during the first 24h.

EVALUATION OF COBBLESTONE MORPHOLOGY

Brightfield images acquired with a 10x objective were analyzed to quantify the cobblestone morphology of RPE monolayers generated in culture. To this aim, we made use of the Steerable Wavelet Filters algorithm (Joshi et al., 2016). In particular, we used two MATLAB packages: “rpe”, a dedicated package to estimate morphology (https://git-bioimage.coe.drexel.edu/opensource/rpe) and “circular-wavelets”, its dependency, and the Steerable Wavelet Filters implementation from the package. We processed the raw images by applying the function “convolve_image_open” and extracted the unnormalized SWF filter response. We divided the images into small tiles of 128x128 pixels and, for each of the tiles, computed the cobblestone junction score of each tile as the total SWF

response per µm². Distributions were then displayed using violin plots to highlight eventual variability of the score in different regions of the image.

FLOW CYTOMETRY and CELL SORTING

hPSC-RPE growing on the tested substrates were dissociated into single cells using TrypLE Select (ThermoFisher Scientific, 12563011). Samples were stained with BV421 Mouse Anti-Human CD140b (BD Biosciences 564124, clone [28D4], 10 µg/mL), BB515 Mouse Anti-Human CD56 (BD Biosciences 564489, clone [B159], 2.5 µg/mL), PE Mouse anti-human Ki-67 (Biolegend 350504, clone [Ki-67], 50 µg/mL) conjugated antibodies, diluted in 2% FBS and 1 mM EDTA (ThermoFisher Scientific, 10082147). Cells were incubated with the conjugated antibodies on ice for 30 min. Fluorescence minus one (FMO) controls were included for each condition to identify and gate negative and positive cells. Stained cells were analyzed using a CytoFLEX flow cytometer equipped with 488 nm, 561 nm, 405 nm and 640 nm lasers (Beckman Coulter). Analysis of the data was carried out using FlowJo v.10 software (Tree Star).

Cell sorting was performed on hPSC-RPE cultures after 30 days of differentiation. Cells were incubated with the mentioned conjugated antibodies on ice for 30 min. Fluorescence minus one (FMO) controls were included for each condition to identify and gate negative and positive cells. Stained cells were then sorted using a BD FACS Aria Fusion Cell Sorter (BD Bioscience) using FACSDiva Software v8.0.1. After sorting, specific cell populations (NCAM1-High, CD140b-High and unsorted counterparts) were cultured under RPE conditions (see replated cells in 2D and 3D hESC-RPE *In Vitro* Differentiation) or under Neuroretinal progenitor conditions (see Neuroretinal Progenitor *In Vitro* Differentiation).

NEURORETINAL PROGENITOR *in vitro* DIFFERENTIATION

Directly after sorting, 68,420 cells/cm^2 were plated on matrigel coated plates (HS980 line). Cells were cultured until confluency in P1 media with DMEM/F12 (ThermoFisher Scientific, 11320033) as basal media containing B27 (ThermoFisher Scientific, 17504044), N2 (ThermoFisher Scientific, 17502048), 10ng/mL hDKK1 (R&D Systems, 5439-DK-010), 10ng/mL mouse Noggin (R&D Systems, 1967-NG-025), 10ng/mL hIGF-1

(R&D Systems, 291-G1-200), 5ng/mL bFGF (ThermoFisher Scientific, 13256029) and 50 U/mL of Penicillin-Streptomycin (ThermoFisher Scientific, 15140122). Thereafter and until day 40, cells were continued cultured in P1 with the addition of 40ng/mL 3,3′,5-Triiodo-L-thyronine T3 (Sigma, T-074-1ML) and 100uM Taurine (Sigma, T8691-25G). This protocol was based on previous work (Shao et al., 2017).

qPCR

Total RNA was isolated using the RNeasy Plus Mini Kit and treated with RNase-free DNase (both from Qiagen, 74106 and 79254, respectively). cDNA was synthesized using 1 µg of total RNA in a 20 µL reaction mixture, containing random hexamers and Superscript III reverse transcriptase (ThermoFisher Scientific, 18080085), according to the manufacturer's instructions. Taq-polymerase together with Taqman probes (ThermoFisher Scientific) for *MITF* (Hs01117294_m1), *BEST-1* (Hs00188249_m1), *RPE65* (Hs01071462_m1), *TYR* (Hs00165976_m1), *SIX6* (HS00201310_m1), *PAX6* (HS01088114_m1), *VSX2* (HS01584046_m1), *RAX* (HS00429459_m1), and *GAPDH* (4333764F) were used. Samples were subjected to the real-time PCR amplification protocol on StepOne™ real-time PCR System (Applied Biosystems). Three independent experiments were performed for every condition and technical duplicates were carried for each reaction. Results are presented as mean ± SEM (standard error of the mean).

ENZYME-LINKED IMMUNOSORBENT ASSAY (ELISA)

Day 30 unsorted, CD140b-High and NCAM1-High sorted hESC-RPE cells were cultured for extra 30 days on Transwell membranes (0.33 cm^2, Merck Millipore, PTHT24H48) coated with hrLN-521 (20 µg/mL). Supernatants from both the apical and basal sides (meaning upper and lower compartments of the transwell, respectively) were collected 60h after the last medium change. PEDF secretion levels were measured in triplicates for each condition with commercially available human PEDF ELISA Kit (BioVendor, RD191114200R), in accordance with manufacturer's instructions. The optical density readings were measured using SpectraMax 250 Microplate Reader (Molecular Devices). Results are presented as mean ± SEM.

TRANSEPITHELIAL ELECTRICAL RESISTANCE (TEER)

Day 30 unsorted, CD140b-High and NCAM1-High sorted hESC-RPE cells were cultured for extra 30 days on Transwell membranes (0.33 cm^2, Merck Millipore, PTHT24H48) coated with hrLN-521 (20 μg/mL). TEER readings were measured using the Millicell Electrical Resistance System volt-ohm meter (Millicell ERS-2 Voltohmmeter, MERS00002, Millipore), in accordance with manufacturer's instructions. Cultures were equilibrated outside the incubator at room temperature for 15–20 min before the start of the readings. Measurements were performed in unchanged culture media in triplicates for each condition and well, at three different well positions. Respective averages were used for further analysis. The background resistance was determined from a blank culture insert in the same media coated with substrate but without cells (also in triplicates). Average blank was subtracted from the averaged triplicates per well. Measurements are reported as resistance in ohms times the surface area in square centimeters ($\Omega \times cm^2$). Results are presented as mean ± SEM.

IMMUNOCYTOFLUORESCENCE

Protein expression of day 60 hPSC-RPE monolayers was assessed with immunofluorescence. Cells were fixed with 4% methanol-free formaldehyde formaldehyde (VWR, FFCHFF22023000) at room temperature for 10 min, followed by permeabilization with 0.3% Triton X-100 (Sigma, T9284) in Dulbecco's phosphate-buffered saline (D-PBS, ThermoFisher Scientific, 14190094) for 10 min and blocking with 4% fetal bovine serum (FBS, ThermoFisher Scientific, 10082147) and 0.1% Tween-20 (Sigma, P9416) in DPBS for 1 hour. Primary antibodies were diluted to the specified concentrations in 4% FBS, 0.1% Tween-20, DPBS solution: VSX2/Chx10 (1:50, Santa Cruz Biotechnology sc-365519, clone [E-12]), Ki67 (1:400, Cell Signaling Technology 9027, clone [D2H10]), Bestrophin 1 (BEST-1) (1:100, Millipore MAB5466), Zonula occludens-1 (ZO-1) (1:100, Invitrogen, 40-2200), RAX (10 ug/mL, Novusbio H00030062-M02, clone [4F4]), PDGFRB (CD140b) (1:100, BD Biosciences, 558820, clone [28D4]) and NCAM1 (CD56) (1:100, BD Biosciences, 555513, clone [B159]). The primary antibodies were incubated overnight at 4°C followed by 2 hours incubation at room temperature with secondary antibodies: donkey anti-mouse IgG (H+L) Alexa Fluor 488,

donkey anti-mouse IgG Alexa Fluor (H+L) 555, donkey anti-mouse IgG Alexa Fluor (H+L) 647, donkey anti-rabbit IgG (H+L) Alexa Fluor 647 (all of them from ThermoFisher Scientific, A21202, A31570, A31571, A31573, respectively) diluted 1:1,000 in 4% FBS, 0.1% Tween-20, D-PBS solution. Nuclei were stained with Hoechst 33342 (1:1,000, Invitrogen H3570). Images were acquired with Zeiss LSM710-NLO point scanning confocal microscope. Post-acquisition analysis of the pictures was performed using ImageJ v2.0 software.

HISTOLOGY and TISSUE IMMUNOSTAINING

Immediately after euthanasia by intravenous injection of 100 mg/kg pentobarbital (Allfatal vet. 100 mg/mL, Omnidea), the eyes were enucleated and the bleb injection area marked with green Tissue Marking Dye (TMD; Histolab Products AB, 02199). An intravitreal injection of 100 µL fixing solution (FS) consisting of 4% buffered formaldehyde (Histolab Products AB, 02175) was performed before fixation in FS for 24-48 hours and embedding in paraffin. Four-µm serial sections were produced through the TMD-labeled area. For immunostaining, slides were deparaffinized in xylene, dehydrated in graded alcohols, and rinsed with ddH_2O and Tris Buffered Saline (TBS, Sigma, 93352, pH 7.6). Antigen retrieval was achieved in 10 mM citrate buffer (trisodium citrate dihydrate, Sigma, S1804, pH 6.0) with 1:2000 Tween-20 (Sigma, P9416) at 96°C for 30 min, followed by 30 min cooling at room temperature. Slides were washed with TBS and blocked for 30 min with 10% Normal Donkey Serum (Abcam, ab138579) diluted in TBS containing 5% (w/v) IgG and protease-free bovine serum albumin (Jackson Immunoresearch, 001-000-162) in a humidified chamber. Primary antibodies diluted in the blocking buffer were incubated overnight at 4°C: human nuclear mitotic apparatus protein (NuMA) (1:200, Abcam ab84680), BEST-1 (1:200, Millipore MAB5466). Secondary antibodies (donkey anti-rabbit IgG (H+L) Alexa Fluor 555 A31572 and donkey anti-mouse IgG (H+L) Alexa Fluor 647 A31571, both from ThermoFisher Scientific) diluted 1:200 in blocking buffer, were incubated 1 hour at room temperature. Sections were mounted with vector vectashield with DAPI mounting medium (Vector Laboratories, H-1200-10) under a 24x50 mm coverslip. Images were taken with an Olympus IX81 fluorescence inverted microscope or

Zeiss LSM710-NLO point scanning confocal microscope. Post-acquisition analysis was performed using ImageJ v2.0 software.

SUBRETINAL INJECTIONS

hESC-RPE monolayers (HS980 line) were washed with DPBS (ThermoFisher Scientific, 14190-094), incubated with TrypLE (ThermoFisher Scientific, 12563-011) and dissociated to single cell suspension as described above. Cells were counted in a Neubauer hemocytometer (VWR, 631-0925) chamber using 0.4% trypan blue (ThermoFisher Scientific, 15250061), centrifuged at 300g for 4 min, and the cell pellet was resuspended in freshly filter-sterilized DPBS (ThermoFisher Scientific, 14190-094) to a final concentration of 1000 cells/µL. The cell suspension was then aseptically aliquoted into 600 µL units and kept on ice until surgery.

After approval by the Northern Stockholm Animal Experimental Ethics Committee (DNR N56/15), two female New Zealand white albino rabbits (provided by the Lidköpings rabbit farm, Lidköping, Sweden) aged 5 months and weighing 3.5 to 4.0 kg were used in this study. All experiments were conducted in accordance with the Statement for the Use of Animals in Ophthalmic and Vision Research.

As previously described (Bartuma et al., 2015; Petrus-Reurer et al., 2017, 2018; Plaza Reyes et al., 2016), animals were put under general anesthesia by intramuscular administration of 35 mg/kg ketamine (Ketaminol 100 mg/mL, Intervet, 511519) and 5 mg/kg xylazine (Rompun vet. 20 mg/mL, Bayer Animal Health, 22545), and the pupils were dilated with a mix of 0.75% cyclopentolate / 2.5% phenylephrine (APL, 321968). Microsurgeries were performed on both eyes using a 2-port 25G transvitreal pars plana technique (Alcon Nordic A/S, 8065751448). 25G trocars were inserted 1 mm from the limbus and an infusion cannula was connected to the lower temporal trocar. The cell suspension was drawn into a 1 mL syringe connected to an extension tube and a 38G polytip cannula (MedOne Surgical Inc, 3219 and 3223). Without infusion or prior vitrectomy the cannula was inserted through the upper temporal trocar. After proper tip positioning, ascertained by a focal whitening of the retina, 50 µL of cell suspension (equivalent to 50,000 cells) was injected slowly subretinally, approximately 6 mm below the inferior margin of the optic nerve head, forming a uniform bleb that was clearly visible

under the operating microscope. To minimize reflux, the tip was maintained within the bleb during the injection. After instrument removal light pressure was applied to the self-sealing suture-less sclerotomies. 2 mg (100 µL) of intravitreal triamcinolone (Triescence 40 mg/mL, Alcon Nordic A/S, 412915) was administered a day prior to the surgery, and no post-surgical antibiotics were given.

GRAFTED HESC-RPE QUANTIFICATION ANALYSIS

Human cells grafted in the RPE layer of the rabbit were recognized by the presence of nuclear NuMA staining and were segmented manually using the label paintbrush tool in *napari* (Sofroniew et al., 2021). The segmentations were used to quantify the total signal for BEST1 and the total level of pigmentation (using the brightfield channel) for each cell. Ten sections were analyzed from three different rabbits, and a total of 227 cells were segmented. An equal number of internal negative control cells were extracted from each image by segmenting cells of the sclera or neural retina. Each image was analyzed independently and then results pooled; the signal for the control cells was used to determine a minimal threshold to consider the cell positive. Specifically, for each image, we used the 97.5th percentile of the signal observed in negative control cells as a threshold for both BEST1 and pigmentation. For combined plotting, the intensity values were renormalized by first computing the square root of the signal and then dividing by the threshold intensity (so that the threshold sits at 1).

RETINAL TISSUE DISSOCIATION of HUMAN ADULT, EMBRYONIC and TRANSPLANTED RABBIT EYES for SINGLE-CELL RNA SEQUENCING

Human post-mortem research-consented donor eyes were obtained from the cornea bank at St. Erik Eye Hospital, Stockholm, Sweden. The use of human tissue was in accordance with the tenets of the Declaration of Helsinki and was approved by the Swedish legislative and ethical committee (#2019-02032) for the use of human donor material for research. Donors did not present any clinical diagnosis of ocular disease, and samples were anonymized and processed under the general data protection regulation. Two human eyes from the same donor were used (45-year-old male, 32 hours post-mortem). The lens was dissected out and the rest of the retina, except the sclera, was

chopped in several small pieces mixed together in 500 µL of digestion buffer (described below). Two embryonic eyes from the same embryo were used from a 7.5 post-conception week embryo. The optic cups were dissected out and chopped in several small pieces to facilitate dissociation in 500 µL of digestion buffer (described below). Donors (deceased, family for adult human eyes, or couples for the embryos) gave their informed consent for the donation and subsequent use for research purposes. The embryonic eyes were acquired from a clinical routine abortion after informed consent by the pregnant woman, in accordance with permissions from the regional ethical review board and the Swedish National Board of Health and Welfare ("Socialstyrelsen" #8.1-11692/2019) and the Swedish Ethical Review Authority ("EPN" #2007/1477-31/3. Two rabbit eyes (from different animals) with 30-day integrated hESC-RPE were enucleated and pigmented areas including the neuroretina, choroid and RPE layer, were dissected out, trimmed and mixed together in 500 µL of digestion buffer. Digestion buffer consisted of: 2mg/ml collagenase IV (ThermoFisher Scientific, 17104019), 120 U/µl DNase I (NEB, Sigma, 4536282001), and 1mg/ml papain (Sigma, 10108014001) in PBS. Eppendorfs containing the samples were rotated and incubated at 37°C on a thermocycler at 300g for 25 min until samples were homogenized. Samples were pipetted every 5 min to digest the tissue sample into single cells. Digestion was stopped by adding equivalent volume of 10% fetal bovine serum (ThermoFisher Scientific, 10082147) in PBS, the samples were filtered using a 30µm MACS Smart Strainer (Miltenyi, 130-098-458) followed by Dead Cell Removal kit (Miltenyi, 130-090-101) to remove dead cells and debris. At this stage, one of the rabbit eye cell samples was stained with mouse anti-human HLA-ABC-FITC (1:20, BD Biosciences, 555552, clone [G46-2.6]), and anti-human HLA-ABC-positive cells were FACS-sorted as specified above, collected and resuspended to 1000 cells/µL in 1% BSA (Sigma, A1470-10g) in PBS for further scRNA-seq. The rest of the samples were also resuspended to 1000 cells/µL in 1% BSA in PBS prior to scRNA-seq.

Acquisition of all primary human tissue samples from two pooled embryonic eyes at Carnegie Stages 12, 13, 14, and 15 (5 post-conception week embryo) was approved by the UCSF Human Gamete, Embryo and Stem Cell Research Committee (GESCR, approval 10-03379 and 10-05113). All experiments were performed in accordance with protocol guidelines. Informed consent was obtained before sample collection and use for

this study. First-trimester human samples were collected from elective pregnancy terminations through the Human Developmental Biology Resource (HDBR), staged using crown-rump length (CRL) and shipped overnight on ice in Rosewell Park Memorial Institute (RPMI) media. Dissections were based upon anatomical landmarks, and dissociations were performed using papain (Worthington). Samples were incubated in papain for 20-30 minutes and triturated manually into a single cell suspension. The samples were filtered for remaining debris and moved to PBS with 0.05% BSA to be captured by 10X Genomics Chromium RNA capture version 2. Library preparation was performed based upon manufacturer's instructions, and sequencing was performed on a NovaSeq S4 lane.

SINGLE-CELL RNA SEQUENCING

Specific stage hESC-RPE cells were trypsinized in TrypLE (10 min, 37°C, 5% CO_2), resuspended to 1000 cells/µL in 0.04% BSA in PBS, and transported at 4°C to the Eukaryotic Single Cell Genomics Facility (ESCG, SciLifeLab, Stockholm, Sweden) where a 3' cDNA library was prepared for scRNA-seq using the 10X Genomics platform and NovaSeq 6000 software. For in house scRNA-seq preparations, cells were collected with 0.04% BSA and counted with the automatized cell count Nucleocounter NC-200 (Chemometec) using the "Cell count and viability assay" and kept at 4°C for less than 1h until loaded onto the Chromium Next GEM Chip G. To process single samples, the Chromium Next GEM Single Cell 3' Reagent Kit v3.1 (Dual Index) was used (10x Genomics, CG000315). Some samples were multiplexed with CellPlex technology, in which cells first underwent the Cell Multiplexing Oligo Labeling Protocol (10x Genomics, CG000391) followed by protocol CB000388, Dual Index to integrate Cell Multiplexing samples. cDNA libraries were sequenced using Illumina Nextseq 2000 Platform 100 cycles P2 and Illumina Nextseq 550 High Output kit v2.5 (150 Cycles).

Cell Ranger 3.1.0 was used to convert Illumina base call files to FASTQ format and to map sequencing reads to the human GRCh38 reference transcriptome with the STAR aligner and generate feature-barcode count matrices. For the E1C3 cell line sequenced at Novo, Cellranger version 3.0.2 was used to demultiplex Illumina base calls and the resulting reads (FASTQ files) were mapped to the Human GRCh38 reference

transcriptome, Ensembl release 90 limited to protein coding genes and lincRNAs. For some older samples, Illumina base call files were converted to FASTQ format using Cell Ranger 2.1.1. Feature-barcode count matrices for all scRNA-seq data are available on GEO under accession number GSE164092. For samples on which RNA velocity was performed, the *velocyto run10x* command was used to produce loom files containing spliced and unspliced RNA information. Dataset quality control, normalization, dimensionality reduction, and visualization were performed using the *scanpy* and *velocyto* modules (La Manno et al., 2018; Wolf et al., 2018). Processed metadata files (h5ad/loom formats) are available on GEO. Jupyter notebooks to reproduce the analysis described below (both ipynb and html formats) are available for download, along with custom, documented source code, at https://github.com/lamanno-epfl. An overview of all scRNA-seq samples obtained in this study can be found in **Table S1**.

scRNA-seq PROCESSING for hESC-RPE *in vitro* DIFFERENTIATION

The initial number of barcoded cells across three cell lines (HS980, KARO1, and E1C3) was 31,879 cells from 19 individual samples (1,790 hESCs, 3,923 cells at D7, 3,453 cells at D14, 4,733 cells at D30, 5,826 cells at D38, 5,587 cells at D45, and 6,567 cells at D60). As a quality-control, cells were filtered based on the UMI count, number of uniquely expressed genes, and percentage of mitochondrial reads according to sample-specific criteria determined by the sequencing depth and coverage, outlined for all samples in **Table S1**. QC filtering resulted in 26,615 single cells: 11,791 HS980 cells (1,016 hESCs, 1,811 at D7, 1,872 at D14, 1,852 at D30, 1,784 at D38, 1,707 at D45, and 1,749 at D60), 6,983 KARO1 cells (885 at D7, 463 at D14, 841 at D30, 1,920 at D38, 1,638 at D45, and 1,236 at D60), and 7,841 E1C3 cells (321 at D7, 180 at D14, 1,153 at D30, 1,602 at D38, 1,793 at D45, and 2,792 at D60). Additional replicates of HS980 (3 total replicates) and E1C3 cell lines (4 total replicates) at D60 yielded 60,593 additional single cells, for a total of 87,208 single cells along the standard 2D monolayer differentiation protocol.

All scRNA-seq samples were first analyzed individually to obtain a UMAP representation and clustered using the standard parameters for normalization, PCA, UMAP, and Louvain clustering from the *scanpy* and *velocyto* packages (La Manno et al., 2018; Wolf et al., 2018). For global analysis of all three lines and seven time points, CCA

integration using a Python implementation of Seurat's integration method was applied to remove batch effects between lines (Butler et al., 2018). Source code and documentation for this custom Python CCA implementation is available at https://github.com/lamanno-epfl. Cell cycle phase assignments were inferred using the function *scanpy.score_genes_cell_cycle* on normalized counts and with published signature genes (Satija et al., 2015; Wolf et al., 2018). Global dimensionality reduction of the entire dataset using 2,000 cv-mean genes was performed using PCA, and the first two principal components derived were visualized. Signature score analysis was performed using a profile of well-studied pluripotency (6 genes: *SOX2, POU5F1, NANOG, ZFP42, LIN28A, SALL4*), retinal progenitor (10 genes: *RAX, OTX2, ZIC2, PAX6, SIX3, SIX6, LHX2, SFRP2, CRABP1, VSX2*) and RPE (15 genes: *TMEFF2, SERPINF1, MITF, PMEL, DCT, ELN, TYRP1, TYR, RLBP1, BEST1, RPE65, TTR, RGR, SFRP5, SLC6A13*) marker genes (Bosze et al., 2020; Fuhrmann, 2010; Schmitt et al., 2009). Global scores for these three cell type identities were computed across all cells by scaling each gene (above) between 0 and 1 and summing total scaled expression for included markers. Pseudotime trajectory inference of differentiating retinal progenitors and RPE at D30 across three cell lines was computed using the *dpt* diffusion pseudotime function in *scanpy*. For the HS980 cell line, SCENIC transcription factor regulon analysis was performed using *pyscenic* and default parameters as described in the original study and subsequent protocol (Aibar et al., 2017; Van de Sande et al., 2020).

Cluster annotation was performed starting with an explorative step followed by an iterative literature check process. The explorative step consisted of extracting the most enriched genes in one cluster as opposed to the others; this is achieved by ranking genes per each cell type by their enrichment score, obtained using the *enrichment.py* code available https://github.com/lamanno-epfl. The iterative literature check process involved using this list to perform literature searches on the basis of which the identity of the cluster is evaluated. More specifically, starting from the top of the list, we searched the literature (using PubMed) for pairs or triplets of enriched genes, and the literature inspection led to cell type hypotheses. Enriched gene lists were also queried using EnrichR (Chen et al., 2013) and, for relevant cell types, benchmarked against existing single cell atlases (La Manno et al., 2021; Lukowski et al., 2019). We then proceeded by attempting to falsify

each of those hypotheses by querying for known markers of the specific cell type and by querying well known markers of related but different cell types. The final cluster annotation was assigned to the cell type hypothesis left standing after this process. The specificity of the gene expression sets that lead to the assignment with respect to the other clusters was evaluated globally by plotting a heatmap displaying all the marker genes by cluster, cell type, or batch (Bosze et al., 2020; Cajal et al, 2012; Chen et al, 2017; Cohen-Salmon et al, 1997, Crespo-Enriquez et al, 2012; Gitton et al, 2011; Kasberg et al, 2013; Kumamoto and Hanashima et al, 2017; Kwon et al, 2010; Lu et al., 2019; McLarren et al., 2003; Pan and Thomson, 2007; Qu et al, 2008; Seo et al, 2017; Soldatov et al, 2019; Tahayato et al, 2003; Yamada et al, 2003).

To evaluate the pluripotency status of all *in vitro* single cells along the standard monolayer differentiation protocol, we trained a random forest classifier (*sklearn.ensemble.RandomForestClassifier)* to assign cells to one of two classes: one "pluripotent" class based on our undifferentiated hESCs and an additional 9,661 stem cells from a recent study (Cuomo et al., 2020) and one "other" class based on our CS12, CS13, CS14, CS15, and W7.5 references as well as external adult RPE references (Quake and Sapiens Consortium, 2021; Voigt et al., 2019). 1,000 cv-mean-enriched genes were used for the classifier; 70% of cells were used for training and 30% for testing. We then scored all *in vitro* cells using this classifier and visualized, by cell line, differentiation day, and replicate, each cell's probability (from 0 to 1) of belonging to the "pluripotent" class.

scRNA-seq PROCESSING for EB 3D DIFFERENTIATION

The initial number of barcoded cells at D7, D14, and D28 of EB differentiation were 205, 1,437, and 1,401 single cells, respectively. As a quality-control, cells were filtered based on the UMI count, number of uniquely expressed genes, and percentage of mitochondrial reads according to sample-specific criteria determined by the sequencing depth and coverage, outlined for all samples in **Table S1**. QC filtering resulted in 181, 1,382, and 1,288 single cells at D7, D14, and D28, respectively. Samples were analyzed using standard procedures for PCA, UMAP, and Louvain clustering using *scanpy*. Signature z-scores for forebrain, midbrain, hindbrain, and rostral neural tube tissues were obtained

using *signatures.py* (available on https://github.com/lamanno-epfl) as well as the following signature genes obtained from and supported by comprehensive single cell atlases: Forebrain (*PAX6, SIX3, FEZF1, RAX, HESX1, LHX2, LHX5, FEZF2, EMX2, SHTN1, IRS4, ARX, OTX1, OTX2, DMRT3, DMRTA2, WNT8B, ZIC4, SFRP1, FOXD4, EMX1, NR2E1, SIX6, VAX2*), Midbrain (*EN1, EN2, PAX5, PAX8, HES3, FAM181B, WNT1, LMX1A, LMX1B, IRX1, FGF18, FLRT1, FGF17, SOX21, ASCL1, TAL2*), Hindbrain (*HOXB2, VGLL3, HOXA2, HOXB1, OLIG3, MSX1, MSX2, CASZ1, FST, TSHZ1*), RetProg (*RAX, OTX2, ZIC2, PAX6, SIX3, LHX2, SFRP2, CRABP1, VSX2*), CrNeCr (*FOXC1, FOXC2, TFAP2A, PITX1, PITX2, ALX1, OTX1, GATA3, FZD10, WNT1*), PrePlac (*SIX1, SIX4, EYA1, EYA2, IRX1, ITX2, ITX3, SOX11, PAX6, OTX2, SIX3, GATA2, GATA3*), and LatNeEp (*DLX5, DLX6, SP8, SP9, DLK1, DLX3, DLX4, SEMA3E*) (Hu et al., 2019; La Manno et al., 2021; Lukowski et al., 2019; Pijuan-Sala et al., 2019). Mapping of 2D monolayer D30 cells (transferred data) onto the 3D EB D28 space (reference data) was performed as follows: correlations were computed between all transferred and reference data points (cells) using the *cdist* function from *scipy.spatial.distance* (Virtanen et al., 2020). Then, the median position of each transferred data point on reference UMAP space was calculated using its five nearest neighbors, by correlation coefficient, in the reference data. Lastly, the function *np.random.normal* was used with mean noise of 0.1 to facilitate easier visualization of multiple transferred cells mapping close to one another on the reference space.

scRNA-seq PROCESSING for D30 SORTED, D60 SORTED, and NON-REPLATING EXPERIMENTS

The initial number of barcoded cells in HS980 NCAM1-High and CD140b-High populations post-sorting was the following: D30 CD140b-high-sorted (1,703 cells), D30 NCAM1-high-sorted (865 cells), D60 CD140b-high-sorted (1,068 cells), D60 NCAM1-high-sorted (1,224 cells), and D60 unsorted (additional replicate; 1,103 cells). As a quality-control, cells were filtered based on the UMI count, number of uniquely expressed genes, and percentage of mitochondrial reads according to sample-specific criteria determined by the sequencing depth and coverage, outlined for all samples in **Table S1**. QC filtering resulted in: D30 CD140b-high-sorted (1,486 cells), D30 NCAM1-high-sorted

(734 cells), D60 CD140b-high-sorted (987 cells), D60 NCAM1-high-sorted (1,106 cells), and D60 unsorted (additional replicate of 975 cells). Samples were analyzed using standard procedures for dimensionality reduction, gene enrichment, UMAP, and clustering using *scanpy*. At D30, CCA integration with *integration.py* (available on https://github.com/lamanno-epfl) was performed between the D30 NCAM1-high-sorted and CD140b-high-sorted samples and the original HS980 D30 unsorted sample, whereas at D60 an additional unsorted sample was obtained using CellPlex with the sorted populations and used in downstream analyses without batch effect correction. Dot plots were created using the *sc.pl.dotplot* function in *scanpy*. Similarly, the initial number of barcoded cells in HS980 D38 Non-Replated and D60 Non-Replated samples were 1,983 and 1,647 cells, respectively. After QC, there were approximately 1,432 D38 and 793 D60 cells used for downstream PCA, UMAP, and clustering with *scanpy*.

scRNA-seq PROCESSING for HUMAN REFERENCE TISSUES

From the embryonic optic vesicles/cups at Carnegie Stage (CS) 12, 13, 14, 15, a total of 9,409 single cells were analyzed (excluding blood cell contaminants) using standard parameters in *scanpy* (CS12: 3,922 cells; CS13: 2,535 cells; CS14: 1,188 cells; CS15: 1,764 cells). Gene enrichment scores were computed and applied to annotate the following cell clusters: RetProg (4,121 cells), MesCh (1,750 cells), RPE (1,512 cells), Other Neural (936 cells), OcSurEct (775 cells), ANR (119 cells), Lens (72 cells), CornEp (54 cells), Endo (40 cells), NrlRet (17 cells), and Immune (13 cells). Visualization of all four samples on a single UMAP was obtained by regressing out batch information in *scanpy* using the *sc.pp.regress_out* command.

2,742 single cells from two human embryonic eyes at week 7.5 (1,439 from eye 1 and 1,303 from eye 2) were analyzed using *velocyto* and *scanpy* after QC. Due to minimal batch effects between fetal eyes, *cv_vs_mean* selection, dimensionality reduction, and other downstream analyses were jointly performed without batch effect correction (3,000 CV-mean genes, 25 PCs, 50 nearest neighbors). Cells were assigned to clusters annotated using gene enrichment as follows: Neural Crest Derived (1,078 cells), Neural Progenitors (706 cells), Retinal Pigment Epithelium (258 cells), Early Ganglion (165 cells), Lens Progenitors (150 cells), Mid Ganglion (104 cells), Late Ganglion (88 cells), Surface

Epithelium (53 cells), Horizontal Cells (36 cells), Lens (35 cells), and Early Photoreceptors (22 cells). Blood cell contaminants were excluded (47 cells).

5,538 single cells from two human adult eyes (4,420 cells from eye 1 and 1,792 from eye 2) were analyzed using *velocyto* and *scanpy* after QC. Due to the limited batch effect between eyes, downstream analyses were performed on the joined expression matrix. To strengthen our reference and ensure accurate capture of all RPE cells, a subset of RPE and melanocyte cells from scRNA-seq in a prior study were included in downstream analysis (reprocessed and filtered according to identical parameters) (Voigt et al., 2019). These 263 additional cells were obtained from 8 samples by performing a clustering of the published, reprocessed counts and selecting cells expressing melanocyte marker *MLANA* or RPE marker *RPE65* (GEO accession number: GSE135922). Adult retinal cell types were annotated using known marker genes and genes with high enrichment scores as follows: Muller Glia (1,646 cells), Rod Photoreceptors (MALAT1-lo) (1,323 cells), Rod Photoreceptors (MALAT1-hi) (747 cells), Melanocytes (364 cells), Fibroblast (267 cells), Cone Photoreceptors (218 cells), Cone Bipolar (195 cells), Amacrine Ganglion (181 cells), Rod Bipolar-ON (169 cells), Corneal Epithelium (161 cells), Immune (136 cells), Retinal Pigment Epithelium (71 cells), and Smooth Muscle (60 cells).

scRNA-seq PROCESSING for NCAM1-High-derived *in vitro* NEURORETINAL PROGENITOR DIFFERENTIATION

NCAM1-High-derived cells from our alternative neuronal differentiation protocol were analyzed using *velocyto* and *scanpy* on 2,638 unfiltered single cells. Cells with UMIs (>6,000 and <100,000) and a low percentage of mitochondrial reads (<10%) were retained during filtering. After dimensionality reduction and UMAP analysis (3,000 CV-mean genes, 20 PCs, 20 nearest neighbors), as well as the removal of a cluster cell contaminant, we obtained 980 single cells of the following clusters, upon which we performed gene enrichment: Mesenchyme (447 cells), Retinal Progenitors (134 cells), Retinal Pigment Epithelium (121 cells), Early Neuroblasts (102 cells), Neuroblasts (77 cells), Glutamatergic Neurons (46 cells), Gaba Neurons (25 cells), Corneal Epithelium (23 cells), and Lens (5 cells).

An integrated subspace was found between NCAM1-High-derived cells (980 cells) and human fetal eyes at week 7.5 (2,742 cells, see above) using CCA integration and 2,000 CV-mean genes for each dataset (3,063 unique genes in total). Gene enrichment scores were computed to identify unique and shared markers between the various populations, including overlapping lens and surface epithelium clusters. RNA velocity was performed on NCAM1-High-derived neurons (Early Neruoblast, Neuroblast, and Glutamatergic Neuron clusters) and fetal retinal ganglion cells (Early, Mid, and Late Ganglion clusters) with *velocyto*.

To compare the differentiation programs of NCAM1-High-derived *in vitro* neurons and fetal retinal ganglion neurons, we fit a principal curve to the two-dimensional UMAP embedding and identified a pseudotemporal cell ordering with which we could perform pseudotime gene expression alignment analysis. Principal curve code is adapted from the original publication and can be found at https://github.com/lamanno-epfl (Hastie and Stuetzle, 1989). Along each pseudotime, a set of five normally distributed and spaced curves were generated using scipy.stats.norm.pdf to mimic different possible expression patterns along the differentiation trajectory (i.e. early downregulation, upregulation midway through the pseudotime followed by downregulation, upregulation at the end of the pseudotime, etc.). Pearson's correlation coefficients were computed between genes and each normally distributed peak in order to identify genes with a distinct temporal upregulation and/or downregulation. For *in vitro* neurons and fetal neurons, the top 50 genes for each of five peaks were combined and compared, enabling us to visualize genes with a similar expression behavior along both pseudotimes as well as genes with a distinct expression behavior unique to either *in vitro* neurons or fetal neurons alone.

scRNA-seq PROCESSING for hESC-RPE *in vivo* RABBIT SUBRETINAL INJECTION

Unlike the other scRNA-seq data in this study, feature-barcode count matrices for hESCs isolated from rabbit retina were generated using a custom-built hybrid reference transcriptome combining both the human GRCh38 reference and the rabbit reference (Oryctolagus cuniculus 2.0.99, EMBL-EBI). FASTQ files were mapped to this hybrid reference with Cell Ranger 3.1.0 to generate count matrices. Reads were exclusively assigned either to a human or rabbit transcript. The percentage of UMIs per cell assigned

to the human or rabbit reference was then computed. Human cells were retained if >80% reads mapped to the human genome. Rabbit cells were retained if >95% reads mapped to the rabbit genome. The resulting number of cells were 24 human cells from the unsorted approach (0.8% of total, Rabbit1, unsorted) and 61 human cells by sorted approach (4.3% of total, Rabbit 2, sorted). As a control, feature-barcode count matrices were also created by aligning FASTQ files solely to the human or rabbit genome with Cell Ranger.

For analysis of human cells, cells with >30% reads mapped to mitochondrial genes were removed, resulting in a total of 65 single cells. Counts were size normalized in scanpy and gene feature selection was performed to select 100 variable genes using cv_vs_mean. Following log-transformation of counts, expression of RPE marker genes and other highly enriched genes was assessed. Differentiation expression analysis was performed by computing fold change on log2 normalized counts.

For analysis of rabbit retina, only cells obtained from the unsorted approach were used in order to avoid any potential cell-type sorting bias. Cells were filtered to retain those with a certain number of UMIs (>2,000 to <50,000), uniquely expressed genes (>600 to <6,000) and mitochondrial reads (<10%). The total number of cells after filtering was 1,965 cells. Dimensionality reduction and clustering analysis was performed using 3,000 cv_vs_mean selected genes and the workflow described above. Cell types were annotated using known marker genes and genes with high enrichment scores as follows: Fibroblast (408 cells), Rod Photoreceptors (338 cells), Smooth Muscles (200 cells), Muller Glia (182 cells), Amacrine Ganglion (156 cells), Rod Bipolar-ON (139 cells), Corneal Epithelium (125 cells), Cone Bipolar-OFF (119 cells), Cone Bipolar-ON (105 cells), Cone Photoreceptors (89 cells), Immune (54 cells), Melanocytes (41 cells), and Retinal Pigment Epithelium (9 cells). For integration of the human adult eye and rabbit eye, anchors were found only between genes with shared nomenclature between the two species' genomes (9,889 genes) and the top enriched 2,000 genes (2,979 genes in total).

GENE ENRICHMENT and SIGNATURE SCORE ANALYSIS

Throughout this study, all marker genes for visualized cell type clusters were selected from among the top 50 ranked genes calculated with a gene enrichment score from

enrichment.py. For each gene, the mean value per cluster was obtained and scaled by the number of cells per cluster with non-zero counts of the given gene. A gene-wise enrichment score was then computed by comparing the means and the fraction of non-zero values among all clusters. Likewise, signature z-scores were computed using a selection of top ranked enriched genes against a randomized set of background gene expression using *signatures.py*. Both custom source codes can be downloaded at https://github.com/lamanno-epfl/rpe_differentiation_profiling_code along with example usage in our Jupyter notebook references (available in both ipynb and html formats).

CELL HETEROGENEITY ESTIMATION

When decomposing the covariance of a heterogeneous cell population, the largest axes of variation typically explain cell type variability, whereas the smaller axes describe subtle phenotypic variation and uncorrelated noise accumulating on the remaining principal components. We deduced that the area under the curve (AUC) of cumulative principal component variance would serve as an insightful metric to assess the overall heterogeneity at each differentiation day. For each time point, *cv_vs_mean* feature selected genes with a score greater than 0.30 were collected, and a union of genes from all time points were retained and size normalized. 800 cells and half the total number of genes were randomly sub-sampled from each time point, without replacement, and PCA analysis was performed. AUC of the cumulative explained variance ratio for all principal components was calculated using *numpy.trapz*. Cells and genes were randomly subsampled for 1000 iterations to obtain a distribution of AUCs at each differentiation day. As a greater deviation from the variance due to biological noise results in a greater AUC of cumulative explained variance, the AUC can therefore be used as a proxy for assessing the overall dataset heterogeneity.

CANONICAL CORRELATION ANALYSIS and DATA INTEGRATION

Canonical correlation analysis (CCA) was performed in all cases using a Python adaptation of the original Seurat method and is available to download at our GitHub repository: https://github.com/lamanno-epfl/ (Butler et al., 2018). For integration of the *in vitro* hESC-RPE D7 and D14 time points, both with and without the cell cycle regressed

out, anchors were found using the top enriched 1,000 genes at each time point obtained with cv_vs_mean in velocyto. For integration of the human fetal week 7.5 and NCAM1-High-derived neuronal hESCs, anchors were found using the top enriched 1,000 genes.

RPE CORRELATION ANALYSIS

We reasoned that reliable retinal progenitor markers would be inversely correlated to genes characterizing more mature cells, such as RPE, and to progenitors for other neuroepithelium tissues. To identify genes strongly linked to a progenitor fate among *in vitro* hESC-RPE D30 cells, Pearson's correlation coefficients were calculated between log-normalized gene counts and either an RPE signature score (16 genes, described above) or a neural tube signature (17 genes: *WNT6*, *COL17A1*, *CDH1*, *TP63*, *KRT19*, *KRT17*, *CRABP2*, *COL3A1*, *CYP26A1*, *FOXC1*, *HAND1*, *SEMA3D*, *SOX17*, *DLX2*, *DLX3*, *DLX4*, *PDGFRA*). For the RPE correlation score, D30 cells from RetProg and RPE secondary clusters were used and for the neural tube correlation score, D30 cells from RPE and CrNeCr secondary clusters were used. Genes with an average spliced expression <0.5 as well as genes included in the signature scores themselves were excluded, resulting in the calculation of RPE signature correlation coefficients for 5,214 genes and of neural signature correlation coefficients for 4,664 genes. Both correlation coefficients were then averaged to obtain the top genes anticorrelated to both signatures and therefore most indicative of a cell progenitor status. Some of these genes possessed known links to anterior placode and epithelium development (Imuta et al, 2009; Yamada et al, 2003; Zhou et al, 2010). The computed correlation coefficients are available in **Table S4**.

RNA VELOCITY ANALYSIS

RNA velocity analysis was performed using *velocyto* and *scvelo,* for dimensionality reduction, KNN smoothing, and gamma fitting we used default parameters unless specified (Bergen et al., 2019; La Manno et al., 2018). For fetal retinal ganglion neurons and NCAM1-High-derived neuronal hESCs, the steady-state implementation of RNA velocity was used (2,000 enriched genes, 20 PCs, 20 nearest neighbors). For estimating RNA velocity of selected *in vitro* hESC-RPE D60 cells, 1,000 CV-mean genes were

selected for velocity estimation. To ensure capture of the appropriate genes, we selected genes with a highly coordinated velocity using a velocity coordination function adapted from velocyto and available at: https://github.com/lamanno-epfl/r. Phase portraits were computed on a gene-wise basis using normalized spliced and unspliced counts, represented on the x-axis and y-axis, respectively. A steady-state fit line was obtained by inferring the degradation rate (γ) according to the standard parameters in *velocyto* (La Manno et al., 2018).

ORDINAL CLASSIFIER

In order to assign *in vitro* RPE cells to an embryonic reference, we constructed an ordinal classifier function in Python and trained it using scRNA-seq data of 783 embryonic RPE and retinal progenitor cells from weeks 5 to 24 of development (Hu et al., 2019) as well as 176 adult RPE reference cells (Voigt et al., 2019). This classifier is based on prior work and relies on training a series of sequential classifiers representing temporally ordered stages of development (Frank and Hall, 2001). Sequential classes were designed by grouping RPE scRNA-seq gene expression training data by similar stages as follows: weeks 5-6 (both retinal progenitors and early RPE), weeks 7-9, weeks 11-13, weeks 17-24, and adult RPE). Classification was performed using log-transformed, size-normalized counts on 2,000 cv-mean enriched genes, excluding mitochondrial and blood-enriched contaminant markers. Results of the classifier were either visualized as scatter plots of each individual cell's predicted ordinal class using *matplotlib.pyplot.scatter* or as kernel density plots averaging a trend for all single classifier cells in a particular sample using the *sklearn.neighbors.KernelDensity* function. To verify the robustness of the classifier, retinal progenitor and RPE cells from our CS13 (W5) and CS20 (W7.5) datasets were classified, as well as 49 additional adults RPE cells from an additional study not used in training of the classifier (Quake and Sapiens Consortium, 2021). The implementation is available as *ordinal_classifier.py* at our GitHub page: https://github.com/lamanno-epfl/.

BIOINFORMATICS SOFTWARE

All analysis was performed using Python 3.7.4. The following modules were used: jupyterlab 1.1.4, loompy 3.0.6, louvain 0.6.1, matplotlib 3.3.1, numpy 1.19.1, pandas

0.25.1, pyscenic 0.10.4, python-igraph 0.7.1, python-louvain 0.13, scanpy 1.4.5, scikit-learn 0.23.2, scipy 1.5.2, scvelo 0.1.25, seaborn 0.9.0, umap-learn 0.4.0, velocyto 0.17.17.

STATISTICAL ANALYSIS

For statistical analysis, one-way ANOVA and posthoc multiple comparisons using Tukey's test correction were performed to assess the *in vitro* differences of the sorted (NCAM1-High, CD140b-High) and unsorted D30 cells in TEER and PEDF secretion assays. Standard error of the mean and standard deviation calculations for all single cell analyses were performed using the *numpy* package in Python across replicates from three cell lines.

REFERENCES

Aibar, S., González-Blas, C.B., Moerman, T., Huynh-Thu, V.A., Imrichova, H., Hulselmans, G., Rambow, F., Marine, J.-C., Geurts, P., Aerts, J., et al. (2017). SCENIC: single-cell regulatory network inference and clustering. Nat. Methods 14, 1083–1086.

Bartuma, H., Petrus-Reurer, S., Aronsson, M., Westman, S., André, H., and Kvanta, A. (2015). In Vivo Imaging of Subretinal Bleb-Induced Outer Retinal Degeneration in the Rabbit. Invest. Ophthalmol. Vis. Sci. 56, 2423–2430.

Bergen, V., Lange, M., Peidli, S., Alexander Wolf, F., and Theis, F.J. (2019). Generalizing RNA velocity to transient cell states through dynamical modeling.

Bosze, B., Hufnagel, R.B., and Brown, N.L. (2020). Chapter 21 - Specification of retinal cell types. In Patterning and Cell Type Specification in the Developing CNS and PNS (Second Edition), J. Rubenstein, P. Rakic, B. Chen, and K.Y. Kwan, eds. (Academic Press), pp. 481–504.

Butler, A., Hoffman, P., Smibert, P., Papalexi, E., and Satija, R. (2018). Integrating single-cell transcriptomic data across different conditions, technologies, and species. Nat. Biotechnol. 36, 411–420.

Cajal, M., Lawson, K.A., Hill, B., Moreau, A., Rao, J., Ross, A., Collignon, J., and Camus, A. (2012). Clonal and molecular analysis of the prospective anterior neural boundary in the mouse embryo. Development 139, 423–436.

Chen, E.Y., Tan, C.M., Kou, Y., Duan, Q., Wang, Z., Meirelles, G.V., Clark, N.R., and Ma'ayan, A. (2013). Enrichr: interactive and collaborative HTML5 gene list enrichment analysis tool. BMC Bioinformatics 14, 128.

Chen, J., Tambalo, M., Barembaum, M., Ranganathan, R., Simões-Costa, M., Bronner, M.E., and Streit, A. (2017). A systems-level approach reveals new gene regulatory modules in the developing ear. Development 144, 1531–1543.

Cohen-Salmon, M., El-Amraoui, A., Leibovici, M., and Petit, C. (1997). Otogelin: a glycoprotein specific to the acellular membranes of the inner ear. Proc. Natl. Acad. Sci. U. S. A. 94, 14450–14455.

Crespo-Enriquez, I., Partanen, J., Martinez, S., and Echevarria, D. (2012). Fgf8-Related Secondary Organizers Exert Different Polarizing Planar Instructions along the Mouse Anterior Neural Tube. PLoS One 7, e39977.

Cuomo, A.S.E., Seaton, D.D., McCarthy, D.J., Martinez, I., Bonder, M.J., Garcia-Bernardo, J., Amatya, S., Madrigal, P., Isaacson, A., Buettner, F., et al. (2020). Single-cell RNA-sequencing of differentiating iPS cells reveals dynamic genetic effects on gene expression. Nat. Commun. 11, 810.

David, F.P.A., Litovchenko, M., Deplancke, B., and Gardeux, V. (2020). ASAP 2020 update: an open, scalable and interactive web-based portal for (single-cell) omics analyses. Nucleic Acids Res. 48, W403–W414.

Frank, E., and Hall, M. (2001). A Simple Approach to Ordinal Classification. In Machine Learning: ECML 2001, (Springer Berlin Heidelberg), pp. 145–156.

Fuhrmann, S. (2010). Eye morphogenesis and patterning of the optic vesicle. Curr. Top. Dev. Biol. 93, 61–84.

Gardeux, V., David, F.P.A., Shajkofci, A., Schwalie, P.C., and Deplancke, B. (2017). ASAP: a web-based platform for the analysis and interactive visualization of single-cell RNA-seq data. Bioinformatics 33, 3123–3125.

Gitton, Y., Benouaiche, L., Vincent, C., Heude, E., Soulika, M., Bouhali, K., Couly, G., and Levi, G. (2011). Dlx5 and Dlx6 expression in the anterior neural fold is essential for patterning the dorsal nasal capsule. Development 138, 897–903.

Hastie, T., and Stuetzle, W. (1989). Principal Curves. J. Am. Stat. Assoc. 84, 502–516.

Hu, Y., Wang, X., Hu, B., Mao, Y., Chen, Y., Yan, L., Yong, J., Dong, J., Wei, Y., Wang, W., et al. (2019). Dissecting the transcriptome landscape of the human fetal neural retina and retinal pigment epithelium by single-cell RNA-seq analysis. PLoS Biol. 17, e3000365.

Imuta, Y., Nishioka, N., Kiyonari, H., and Sasaki, H. (2009). Short limbs, cleft palate, and delayed formation of flat proliferative chondrocytes in mice with targeted disruption of a putative protein kinase gene, Pkdcc (AW548124). Dev. Dyn. 238, 210–222.

Joshi, R., Mankowski, W., Winter, M., Saini, J.S., Blenkinsop, T.A., Stern, J.H., Temple, S., and Cohen, A.R. (2016). Automated Measurement of Cobblestone Morphology for Characterizing Stem Cell Derived Retinal Pigment Epithelial Cell Cultures. J. Ocul. Pharmacol. Ther. 32, 331–339.

Kasberg, A.D., Brunskill, E.W., and Steven Potter, S. (2013). SP8 regulates signaling centers during craniofacial development. Dev. Biol. 381, 312–323.

Kumamoto, T., and Hanashima, C. (2017). Evolutionary conservation and conversion of Foxg1 function in brain development. Dev. Growth Differ. 59, 258–269.

Kwon, H.-J., Bhat, N., Sweet, E.M., Cornell, R.A., and Riley, B.B. (2010). Identification of early requirements for preplacodal ectoderm and sensory organ development. PLoS Genet. 6, e1001133.

La Manno, G., Soldatov, R., Zeisel, A., Braun, E., Hochgerner, H., Petukhov, V., Lidschreiber, K., Kastriti, M.E., Lönnerberg, P., Furlan, A., et al. (2018). RNA velocity of single cells. Nature 560, 494–498.

La Manno, G., Siletti, K., Furlan, A., Gyllborg, D., Vinsland, E., Mossi Albiach, A., Mattsson Langseth, C., Khven, I., Lederer, A.R., Dratva, L.M., et al. (2021). Molecular architecture of the developing mouse brain. Nature 596, 92–96.

Lidgerwood, G.E., Senabouth, A., Smith-Anttila, C.J.A., Gnanasambandapillai, V., Kaczorowski, D.C., Amann-Zalcenstein, D., Fletcher, E.L., Naik, S.H., Hewitt, A.W., Powell, J.E., et al. (2021). Transcriptomic Profiling of Human Pluripotent Stem Cell-derived Retinal Pigment Epithelium over Time. Genomics Proteomics Bioinformatics 19, 223–242.

Lu, Y., Shiau, F., Yi, W., Lu, S., Wu, Q., Pearson, J.D., Kallman, A., Zhong, S., Hoang, T., Zuo, Z., et al. (2020). Single-Cell Analysis of Human Retina Identifies Evolutionarily Conserved and Species-Specific Mechanisms Controlling Development. Dev. Cell 53, 473–491.e9.

Lukowski, S.W., Lo, C.Y., Sharov, A.A., Nguyen, Q., Fang, L., Hung, S.S., Zhu, L., Zhang, T., Grünert, U., Nguyen, T., et al. (2019). A single-cell transcriptome atlas of the adult human retina. EMBO J. 38, e100811.

McLarren, K.W., Litsiou, A., and Streit, A. (2003). DLX5 positions the neural crest and preplacode region at the border of the neural plate. Dev. Biol. 259, 34–47.

Pan, G., and Thomson, J.A. (2007). Nanog and transcriptional networks in embryonic stem cell pluripotency. Cell Res. 17, 42–49.

Petrus-Reurer, S., Bartuma, H., Aronsson, M., Westman, S., Lanner, F., André, H., and Kvanta, A. (2017). Integration of Subretinal Suspension Transplants of Human Embryonic Stem Cell-Derived Retinal Pigment Epithelial Cells in a Large-Eyed Model of Geographic Atrophy. Investigative Opthalmology & Visual Science 58, 1314.

Petrus-Reurer, S., Bartuma, H., Aronsson, M., Westman, S., Lanner, F., and Kvanta, A. (2018). Subretinal Transplantation of Human Embryonic Stem Cell Derived-retinal Pigment Epithelial Cells into a Large-eyed Model of Geographic Atrophy. J. Vis. Exp. I 131, 56702.

Pijuan-Sala, B., Griffiths, J.A., Guibentif, C., Hiscock, T.W., Jawaid, W., Calero-Nieto, F.J., Mulas, C., Ibarra-Soria, X., Tyser, R.C.V., Ho, D.L.L., et al. (2019). A single-cell molecular map of mouse gastrulation and early organogenesis. Nature 566, 490–495.

Plaza Reyes, A., Petrus-Reurer, S., Antonsson, L., Stenfelt, S., Bartuma, H., Panula, S., Mader, T., Douagi, I., André, H., Hovatta, O., et al. (2016). Xeno-Free and Defined Human Embryonic Stem Cell-Derived Retinal Pigment Epithelial Cells Functionally Integrate in a Large-Eyed Preclinical Model. Stem Cell Reports 6, 9–17.

Plaza Reyes, A., Petrus-Reurer, S., Padrell Sanchez, S., Kumar, P., Douagi, I., Bartuma, H., Aronsson, M., Westman, S., Lardner, E., Andre, H., et al. (2020a). Identification of cell

surface markers and establishment of monolayer differentiation to retinal pigment epithelial cells. Nat. Commun. 11, 1609.

Plaza Reyes, A., Petrus-Reurer, S., Sánchez, S.P., Kumar, P., Douagi, I., Bartuma, H., Aronsson, M., Westman, S., Lardner, E., Falk, A., et al. (2020b). Xeno-free, chemically defined and scalable monolayer differentiation protocol for retinal pigment epithelial cells. PROTOCOL (Version 1) available at Protocol Exchange [https://doi.org/10.21203/rs.3.pex-635/v1].

Qu, X.-B., Pan, J., Zhang, C., and Huang, S.-Y. (2008). Sox17 facilitates the differentiation of mouse embryonic stem cells into primitive and definitive endoderm in vitro. Dev. Growth Differ. 50, 585–593.

Quake, S.R., and Sapiens Consortium, T. (2021). The Tabula Sapiens: a single cell transcriptomic atlas of multiple organs from individual human donors. Biorxiv.

Rodin, S., Antonsson, L., Niaudet, C., Simonson, O.E., Salmela, E., Hansson, E.M., Domogatskaya, A., Xiao, Z., Damdimopoulou, P., Sheikhi, M., et al. (2014). Clonal culturing of human embryonic stem cells on laminin-521/E-cadherin matrix in defined and xeno-free environment. Nat. Commun. 5, 3195.

Satija, R., Farrell, J.A., Gennert, D., Schier, A.F., and Regev, A. (2015). Spatial reconstruction of single-cell gene expression data. Nat. Biotechnol. 33, 495–502.

Schmitt, S., Aftab, U., Jiang, C., Redenti, S., Klassen, H., Miljan, E., Sinden, J., and Young, M. (2009). Molecular characterization of human retinal progenitor cells. Invest. Ophthalmol. Vis. Sci. 50, 5901–5908.

Sofroniew, N., Lambert, T., Evans, K., Nunez-Iglesias, J., Bokota, G., Peña-Castellanos, G., Winston, P., Yamauchi, K., Bussonnier, M., Pop, D.D., et al. (2021). napari/napari: 0.4.12rc2.

Soldatov, R., Kaucka, M., Kastriti, M.E., and Petersen, J. (2019). Spatiotemporal structure of cell fate decisions in murine neural crest. Science. 2019 Jun 7;364(6444):eaas9536.

Seo, S., Chen, L., Liu, W., Zhao, D., Schultz, K.M., Sasman, A., Liu, T., Zhang, H.F., Gage, P.J., and Kume, T. (2017). Foxc1 and Foxc2 in the Neural Crest Are Required for Ocular Anterior Segment Development. Invest. Ophthalmol. Vis. Sci. 58, 1368–1377.

Tahayato, A., Dollé, P., and Petkovich, M. (2003). Cyp26C1 encodes a novel retinoic acid-metabolizing enzyme expressed in the hindbrain, inner ear, first branchial arch and tooth buds during murine development. Gene Expr. Patterns 3, 449–454.

Van de Sande, B., Flerin, C., Davie, K., De Waegeneer, M., Hulselmans, G., Aibar, S., Seurinck, R., Saelens, W., Cannoodt, R., Rouchon, Q., et al. (2020). A scalable SCENIC workflow for single-cell gene regulatory network analysis. Nat. Protoc. 15, 2247–2276.

Virtanen, P., Gommers, R., Oliphant, T.E., Haberland, M., Reddy, T., Cournapeau, D., Burovski, E., Peterson, P., Weckesser, W., Bright, J., et al. (2020). SciPy 1.0: fundamental algorithms for scientific computing in Python. Nat. Methods 17, 261–272.

Voigt, A.P., Mulfaul, K., Mullin, N.K., Flamme-Wiese, M.J., Giacalone, J.C., Stone, E.M., Tucker, B.A., Scheetz, T.E., and Mullins, R.F. (2019). Single-cell transcriptomics of the human retinal pigment epithelium and choroid in health and macular degeneration. Proc. Natl. Acad. Sci. U. S. A. 116, 24100–24107.

Wolf, F.A., Angerer, P., and Theis, F.J. (2018). SCANPY: large-scale single-cell gene expression data analysis. Genome Biol. 19, 15.

Yamada, R., Mizutani-Koseki, Y., Hasegawa, T., Osumi, N., Koseki, H., and Takahashi, N. (2003). Cell-autonomous involvement of Mab21l1 is essential for lens placode development. Development 130, 1759–1770.

Zhou, J., Wang, C., Wang, Z., Dampier, W., Wu, K., Casimiro, M.C., Chepelev, I., Popov, V.M., Quong, A., Tozeren, A., et al. (2010). Attenuation of Forkhead signaling by the retinal determination factor DACH1. Proc. Natl. Acad. Sci. U. S. A. 107, 6864–6869.
